# LINC00478-derived novel cytoplasmic lncRNA LacRNA stabilizes PHB2 and suppresses breast cancer metastasis via repressing MYC targets

**DOI:** 10.1186/s12967-023-03967-1

**Published:** 2023-02-13

**Authors:** Rong Guo, Yonghui Su, Qi Zhang, Bingqiu Xiu, Sheng Huang, Weiru Chi, Liyi Zhang, Lun Li, Jianjing Hou, Jia Wang, Jiajian Chen, Yayun Chi, Jingyan Xue, Jiong Wu

**Affiliations:** 1 Department of Breast Surgery, Fudan University Shanghai Cancer Center, Shanghai, 200032 People’s Republic of China; 2 Department of Breast Surgery, The Third Affiliated Hospital of Kunming Medical University, Yunnan Cancer Hospital, Kunming, 650000 People’s Republic of China; 3 Department of Oncology, Shanghai Medical College, Fudan University, Shanghai, 200032 People’s Republic of China

**Keywords:** Breast cancer, LacRNA, Metastasis, PHB2, c-Myc

## Abstract

**Background:**

Metastasis is the predominant cause of mortality in patients with breast cancer. Long noncoding RNAs (lncRNAs) have been shown to drive important phenotypes in tumors, including invasion and metastasis. However, the lncRNAs involved in metastasis and their molecular and cellular mechanisms are still largely unknown.

**Methods:**

The transcriptional and posttranscriptional processing of LINC00478-associated cytoplasmic RNA (LacRNA) was determined by RT-qPCR, semiquantitative PCR and 5′/3′ RACE. Paired-guide CRISPR/cas9 and CRISPR/dead-Cas9 systems was used to knock out or activate the expression of LacRNA. Cell migration and invasion assay was performed to confirm the phenotype of LacRNA. Tail vein model and mammary fat pad model were used for in vivo study. The LacRNA-PHB2-cMyc axis were screened and validated by RNA pulldown, mass spectrometry, RNA immunoprecipitation and RNA-seq assays.

**Results:**

Here, we identified a novel cytoplasmic lncRNA, LacRNA (LINC00478-associated cytoplasmic RNA), derived from nucleus-located lncRNA LINC00478. The nascent transcript of LINC00478 full-length (LINC00478_FL) was cleaved and polyadenylated, simultaneously yielding 5′ ends stable expressing LacRNA, which is released into the cytoplasm, and long 3′ ends of nuclear-retained lncRNA. LINC00478_3′RNA was rapidly degraded. LacRNA significantly inhibited breast cancer invasion and metastasis in vitro and in vivo. Mechanistically, LacRNA physically interacted with the PHB domain of PHB2 through its 61–140-nt region. This specific binding affected the formation of the autophagy degradation complex of PHB2 and LC3, delaying the degradation of the PHB2 protein. Unexpectedly, LacRNA specifically interacted with PHB2, recruited c-Myc and promoted c-Myc ubiquitination and degradation. The negatively regulation of Myc signaling ultimately inhibited breast cancer metastasis. Furthermore, LacRNA and LacRNA-mediated c-Myc signaling downregulation are significantly associated with good clinical outcomes, take advantage of these factors we constructed a prognostic predict model.

**Conclusion:**

Therefore, our findings propose LacRNA as a potential prognostic biomarker and a new therapeutic strategy.

**Supplementary Information:**

The online version contains supplementary material available at 10.1186/s12967-023-03967-1.

## Introduction

Breast cancer is the most frequently diagnosed cancer in females and the leading cause of cancer-related death worldwide [1]. Although metastasis is the predominant cause of mortality in patients with solid tumors, the molecular and cellular mechanisms underlying cancer metastasis remain largely unknown [2, 3]. Thus, it is essential to identify molecular mechanisms underlying breast cancer metastasis and develop new metastatic biomarkers and therapeutic strategies.

Most of the cancer genome is transcribed yielding a complex network of overlapping transcripts that includes tens of thousands of long noncoding RNAs (lncRNAs) with no protein-coding capacity [4–6]. lncRNAs have recently emerged as critical mediators of tumor progression through their regulation of both oncogenic and tumor suppressive pathways [7, 8]. lncRNAs have been shown to drive important phenotypes in tumors, including tumor invasion and metastasis [9–12]. However, despite these recent findings, the regulatory role of lncRNAs in mediating cellular processes in cancer development remains an area of active investigation and the subject of controversy [4].

A significant amount of lncRNAs do not function in the original transcriptional form, but can serve as precursors for shorter and mature lncRNAs, and gain biological functions during this process [13, 14]. LINC00478-associated cytoplasmic RNA (LacRNA) is one of that type of lncRNA which is derived from 5′-end of lncRNA LINC00478. LacRNA is an 817-nt novel mature cytoplasmic noncoding RNA. We demonstrated the critical role of LacRNA in breast cancer metastasis. We revealed the interaction between LacRNA and a known tumor suppressor, PHB2, which mediates the stability of the PHB2 protein. We reveal a critical role of the LacRNA-PHB2 complex in inhibiting c-Myc, which downregulated Myc target genes (e.g., CDC20 and CDC45), ultimately suppressing breast cancer metastasis. Extensive analyses of clinical data indicated that LacRNA was significantly associated with good clinical outcomes and represented an independent prognostic predictor. Collectively, LacRNA functions as a tumor suppressor lncRNA that inhibits the breast cancer invasion–metastasis cascade. LacRNA might be a promising prognostic predictor and a new cancer therapeutic strategy.

## Results

### LacRNA is a small cytoplasm RNA derived from LINC00478 in breast cancer cells.

To investigate the role of lncRNAs in breast cancer metastasis, we used microarray analysis to establish lncRNA expression profiles in primary breast tumors and paired metastatic lymph nodes. After screening lncRNAs that were annotated in the RefSeq and ENSEMBL databases and excluded pseudogenes, small nuclear RNAs (snRNAs), and small nucleolar RNAs (Additional file 1: Fig. S1a, b), we found that six lncRNAs that were downregulated and four lncRNAs that were upregulated by more than 1.5-fold in metastatic tissues compared to primary tumor tissues (Additional file 1: Fig. S1c, Table S1).

The six lncRNAs that were downregulated in metastatic tissues were considered as tumor suppressor genes and were investigated further. To evaluate the clinical significance of the six lncRNAs, we first queried The Cancer Genome Atlas (TCGA) breast cancer dataset to systemically assess the 6 candidate lncRNAs (Additional file 1: Fig. S1a). A total of 105 paired primary breast cancer and adjacent normal tissues were used for analysis, and LINC01614, VCAN-AS1 and LINC00478 were found to be differentially expressed (Additional file 1: Fig. S1d). However, only LINC00478 was downregulated in breast cancer tissues compared to adjacent normal tissues (*P* < 0.0001), which was in accordance with previous results (Additional file 1: Fig. S1d). Furthermore, RNA-seq and overall survival (OS) data for 837 breast cancer patients in TCGA database were analyzed to identify lncRNAs that may play a role in cancer progression. A higher expression level of LINC00478 in TCGA breast cancer samples was associated with better OS (hazard ratio (HR) = 0.535, 95% confidence interval (CI): 0.308–0.930, *P* = 0.027) (Additional file 1: Fig. S1e). Thus, we focused on LINC00478 as a metastasis-associated lncRNA in further studies.

**Fig. 1:**
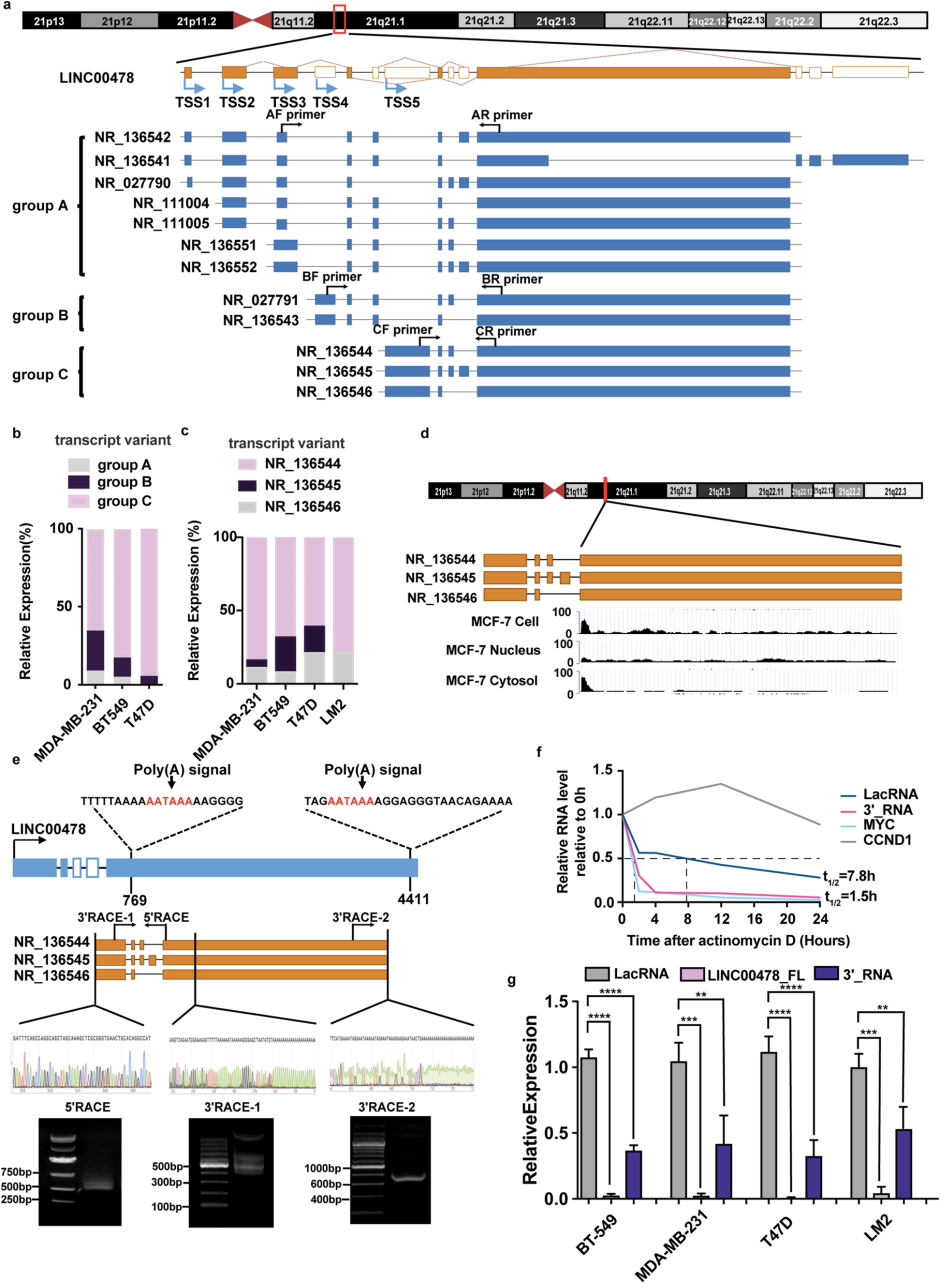
LacRNA is a small cytoplasm RNA derived from LINC00478 in breast cancer cells. **a** Schematic view of chromosomal location of LINC00478 and the 14 exons, 12 main alternative splices in UCSC browser. The alternative splices were divided into three groups, group A, group B and group C for further RT-qPCR analysis. RT-qPCR primers for each group were marked at the corresponding position of exons. **b** Relative RNA expression of 3 groups of LINC00478 isoforms assayed by RT-qPCR in MDA-MB-231, BT549 and T47D cells. Normalized to GAPDH. The data shown represent three independent experiments. **c** Relative RNA expression of 3 isoforms included in group C assayed by RT-qPCR in MDA-MB-231, BT549, T47D and LM2 cells. Normalized to GAPDH. The data shown represent three independent experiments. **d** RNA-seq data from UCSC browser of MCF-7 cell, showed the reads differently distributed in the last exon both in MCF-7 total cell and cytosol. **e** The 5′- and 3′- rapid amplification (5′/3′ RACE) and Sanger sequencing of cDNA ends of NR_136544, NR_136546 and NR_136545. Representative images of PCR products from the 5′/3′ RACE. Sequencing of RACE products are shown. **f** Half-life of LacRNA and LINC00478_3′RNA in MDA-MB-231 cells treated with 5 μg/ml actinomycin D for the indicated times. Myc served as the positive control. CCND1 served as the negative control. RNA was extracted and assayed by RT-qPCR. The data shown represent three independent experiments. **g** The relative expression of LacRNA, LINC00478_3′RNA and LINC00478_FL (LINC00478 full-length) in BT-549, MDA-MB-231, T47D and LM2 cells according to RT-qPCR. Mean ± SEM; n = 3 independent experiments

Human LINC00478 is encoded by the gene on chromosome 21q21.1. It is a lncRNA of ~ 4548 nucleotides (nt), with different isoforms generated by alternative splicing (Fig. 1a). The existing annotations of the LINC00478 gene in the University of California, Santa Cruz (UCSC) Genome Browser predicted the presence of 12 main transcript isoforms that differ in 14 exons and 5 transcriptional start sites (TSS) (Fig. 1a). We divided the 12 main transcript variants into three groups for RT-qPCR analysis, AF/AR, BF/BR and CF/CR primers for groups A, B, and C, respectively (Fig. 1a). Group C included the most expressed isoforms in the MDA-MB-231, BT549, and T47D cell lines (Fig. 1b). Group C included three isoforms, NR_136544, NR_136546, and NR_136545 (Fig. 1a). RT-qPCR (Fig. 1c) and semi-quantitative PCR (Additional file 1: Fig. S2a) confirmed that NR_136544 was mainly expressed variant in breast cancer cells.

Interestingly, we browsed UCSC and found that the last exon was differentially expressed in MCF-7 cells (Fig. 1d). The 5′ end of the last exon showed significant increased expression compared to the 3′ end in the total cells and cytosol (Fig. 1d). Therefore, we used 5′- and 3′-rapid amplification of cDNA ends (5′/3′ RACE) to determine the exact structure in breast cancer cells. The 5′ RACE demonstrated again that isoforms of group C were mainly expressed, because 5′ RACE could not successfully amplify the isoforms of groups A and group B, but only the isoforms of group C (Fig. 1e). 3′ RACE with two different primers successfully demonstrated the existence of two different 3′ ends (Fig. 1e). Sanger sequencing of the products of RACE showed that both 3′ ends had poly A tails (Fig. 1e). In conclusion, the last exon of NR_136544 may be cleaved by RNase to simultaneously generate the mature 5′ end of cytoplasmic RNA and 3′ end of nuclear RNA by polyadenylation. We named the 5′ end RNA as LINC00478-associated cytoplasmic RNA (LacRNA) and the 3′ end of nuclear RNA as LINC00478_3′RNA. RT-qPCR experiments using oligo dT or random primers to reverse-transcribe total cell RNA confirmed that LacRNA had a poly A tail (Additional file 1: Fig. S2b). The full sequence of LacRNA was showed in Additional file 1: Fig. S2c.

We also observed that LacRNA was expressed at higher levels in Luminal- and HER2-type breast cancer cell lines (e.g., BT-474, SK-BR-3, T47D, MCF-7) than in TNBC cell lines (e.g., MDA-MB-231 and MDA-MB-436), which were more aggressive and metastatic (Additional file 1: Fig. S2d). Nuclear/cytoplasmic fractionation again demonstrated that LacRNA was predominately located in the cytoplasm of MDA-MB-231, BT-549, and T47D cells (Additional file 1: Fig. S2e), which was confirmed by RNA-scope (Additional file 1: Fig. S2f). This suggests that LacRNA may exert its biological function in the cytoplasm. The experiments indicated that LINC00478_3′RNA was located in the nuclear compartment (Additional file 1: Fig. S2e). The half-life of LINC00478_3′RNA (~ 1.5 h) was much shorter than that of LacRNA (~ 7.8 h) in MDA-MB-231 cells after transcriptional inhibition by actinomycin (Fig. 1f). Different qPCR primers were used to detect the relative expression levels of LacRNA, LINC00478_3′RNA, and full-length LINC00478 (LINC00478_FL) (Fig. 1g). We found that almost all LINC00478_FL was cleaved, and the LacRNA level was more than two times higher than the LINC00478_3′RNA level (Fig. 1g). Consequently, LacRNA may be a fundamental transcript variant of LINC00478 in breast cancer cells. Neither LINC00478_FL nor LacRNA had coding potential, according to predictions by the coding potential assessment tool (CPAT) and coding potential calculator (CPC) [15, 16] (Additional file 1: Fig. S2g).

### LacRNA inhibits breast cancer metastasis

**Fig. 2:**
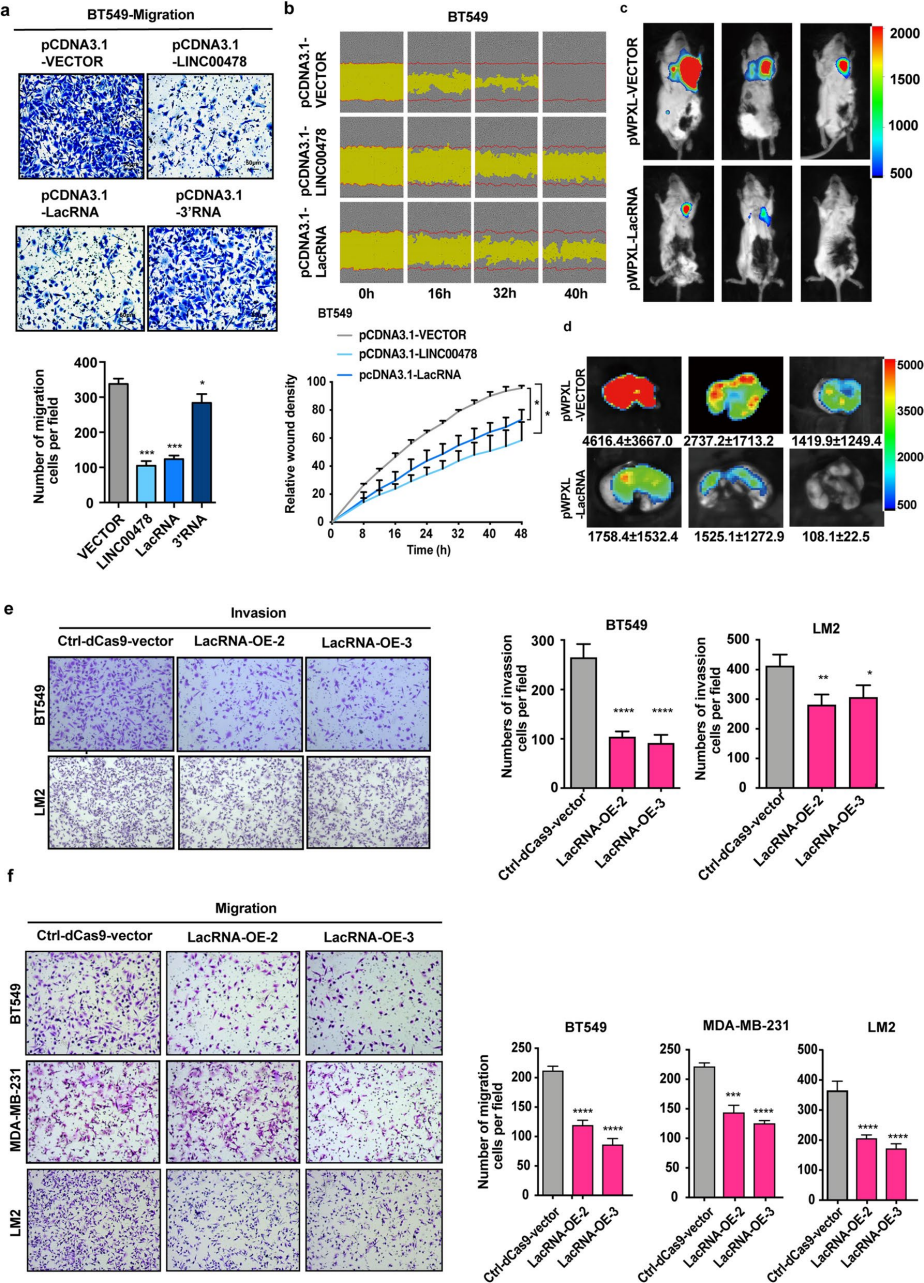
Gain-of-function assays showed that LacRNA inhibits breast cancer metastasis both in vitro and in vivo. **a** Representative images of transwell migration assays for BT-549 cells overexpressing LINC00478_FL, LacRNA and LINC00478_3′RNA (scale bars, 50 μm). **b** Wound healing assay in BT549 cells overexpressing LINC00478_FL and LacRNA. **c**, **d** Representative images of bioluminescence imaging (BLI) of the lungs of NOD/SCID mice 7 weeks after orthotopic injection with vector pWPXL-LacRNA and control pWPXL-vector LM2 cells, n = 6. **e**, **f** Transwell migration and invasion assays of BT-549, MDA-MB-231 and T47D cells with LacRNA activation induced by CRISPR/dead-Cas9 technology. Values are expressed as the Mean ± SEM in a, b, e and f. Statistical significance was determined by one-way ANOVA. *p < 0.05, **p < 0.01, ***p < 0.001, **** *p* < 0.0001. The stable cells established via the CRISPR/dead-Cas9 activation technology were designated as OE

Overexpression of LacRNA and LINC00478_FL inhibited the migration of BT549 cells (Fig. 2a, b). LINC00478_3′ RNA did not affect cell function (Fig. 2a). Ectopic overexpression of LINC00478_FL caused a significant increase in LacRNA expression in breast cancer, suggesting that most ectopic LINC00478_FL can be cleaved into LacRNA and 3′RNA (Additional file 1: Fig. S3a). The function of LacRNA was also confirmed in MDA-MB-231 and MDA-MB-231/LM2 cells when LacRNA was overexpressed using the pWPXL vector (Additional file 1: Fig. S3b–f). It is apparent to us that LacRNA is the functional isoform of LINC00478. At the same time, the significant metastasis inhibition function of exogenous overexpression of LacRNA indicated that the positive function was not attributed to the microRNAs in its introns, which include let-7c, miR-125b, and miR-99a. Therefore, we used LacRNA for further studies. To further characterize the impact of LacRNA on cancer metastasis in vivo, orthotopic xenograft models of NOD/SCID mice were used. MDA-MB-231/LM2 LacRNA-overexpressing cells (pWPXL-LacRNA) were inoculated into the mammary fatty pads of mice. We found that the growth rate of xenograft tumors was substantially inhibited, and the tumor volumes were significantly reduced when LacRNA was overexpressed without influencing the body weight of the mice (Additional file 1: Fig. S3g, h). In addition to reduced tumor growth, overexpression of LacRNA inhibited breast cancer metastasis to the lungs (Additional file 1: Fig. S3i, Fig. 2c, d). The orthotopic tumors were removed and the expression of LacRNA in the orthotopic tumors was verified by qPCR (Additional file 1: Fig. S3j). It was confirmed that the expression level of LacRNA in the pWPXL-LacRNA group was also significantly higher than that in the control group, demonstrating that expression level of LacRNA in orthotopic tumors was not lost in vivo (Additional file 1: Fig. S3j).

The CRISPR/dead-Cas9 system was used to activate endogenous RNA levels of LacRNA [17] (Additional file 1: Fig. S4a, b). Inhibition of cell migration and invasion was also observed in BT-549, MDA-MB-231, and MDA-MB-231/LM2 cells when endogenous LacRNA was accumulated with the CRISPR/dead-Cas9 system (Fig. 2e, f, Additional file 1: Fig. S4c). The expression of microRNAs in its introns did not increase significantly during the activation process (Additional file 1: Fig. S4d). To further investigate the effect of LacRNA on tumor invasion in vivo, we developed a metastasis model by injecting cancer cell suspension into the caudal vein of female athymic BALB/c nude mice. Accordingly, LacRNA activated via the CRISPR/Cas9 system in MDA-MB-231/LM2 cells (LacRNA-OE3), significantly reduced lung metastasis as well (Additional file 1: Fig. S4e, f). These results support the notion that LacRNA plays an important role in breast cancer metastasis.

**Fig. 3:**
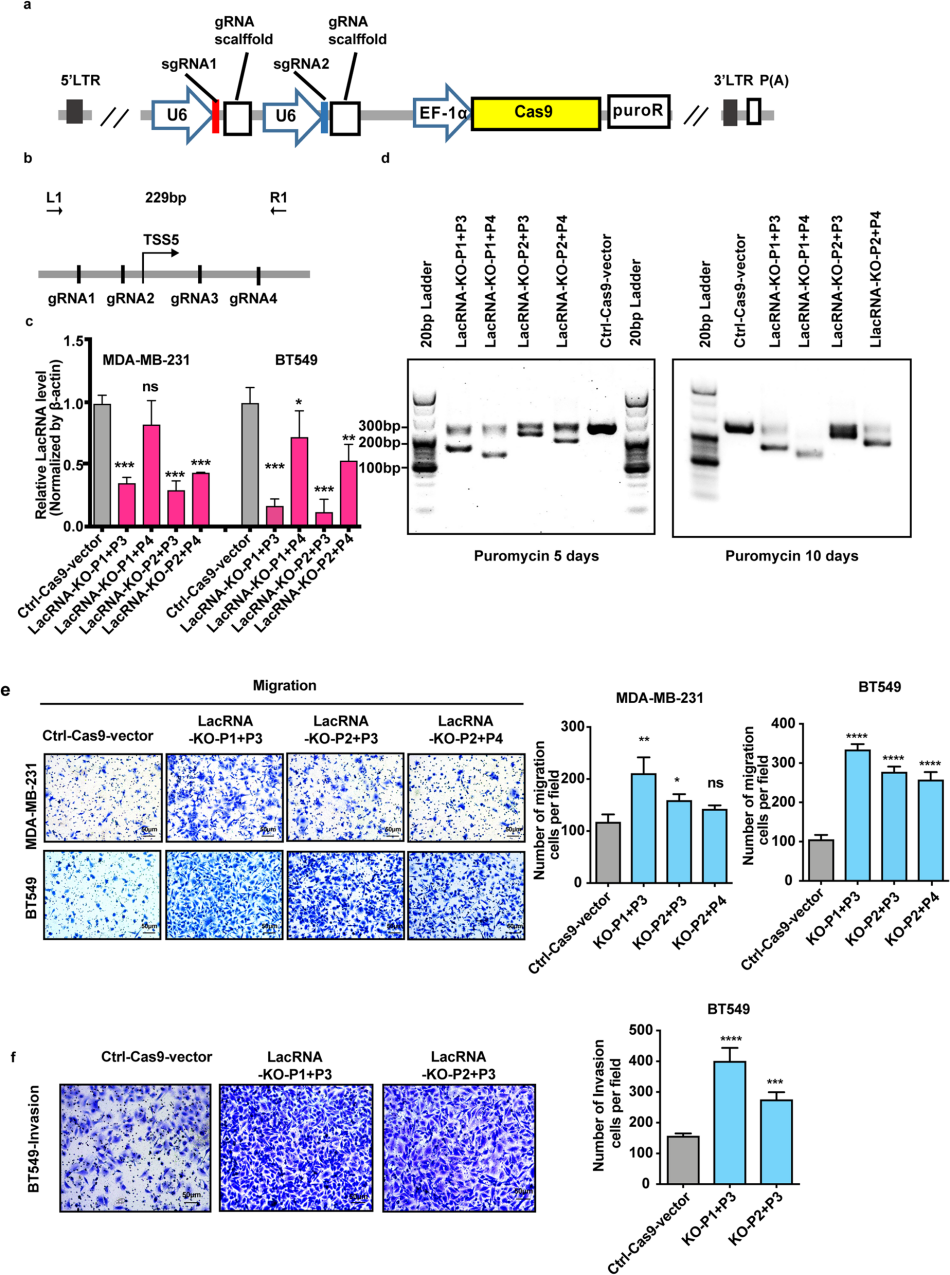
Knock out of LacRNA promotes breast cancer metastasis.** a** Schematic diagram of paired-gRNA plasmid construction. Paired gRNAs (pgRNAs) in one lentiviral backbone with two U6 promoters separately driving the two gRNAs. **b** Schematic diagram of the position of the four candidate sgRNA#1-#4 before and after LacRNA genome transcriptional start site (tss5) and the position of genomic PCR validation primers (L1 and R1 primers). **c** The knockout efficiency of LacRNA by CRISPR-Cas9 technology knockout in the MDA-MB-231 and BT549 cells. Values are expressed as the Mean ± SEM; n = 3 independent experiments. *P* values were determined by one way analysis of variance (ANOVA). ns, not significant, **P < 0.01, ***P < 0.001. **d** After infection with paired-gRNA, puromycin was screened for 5 and 10 days, and the deletion of genomic DNA was verified by PCR. **e**, **f** Transwell migration and invasion assays of BT-549 and MDA-MB-231 cells with LacRNA knockout by CRISPR/Cas9 technology. The stable cells established via the CRISPR/Cas9 knockout technology were designated as KO. Values are expressed as the Mean ± SEM in c, e and f. n = 3 independent experiments. *P* values were determined by one-way analysis of variance (ANOVA). ns, not significant, *P < 0.05, **P < 0.01, ***P < 0.001, ****P < 0.0001

A paired-guide CRISPR/Cas9 system was used to knock out endogenous LacRNA, which yielded satisfactory results (Fig. 3a–c). Four guide RNAs were designed to target LacRNA (Additional file 1: Table S2). We expressed the paired gRNAs (pgRNAs) in one lentiviral backbone with two U6 promoters separately driving the two gRNAs [18] (Fig. 3a). The pgRNAs generated large genomic deletions of LacRNA with the correct sizes and high efficiency [19] (Fig. 3b–d). Conversely, knockout of LacRNA using the CRISPR/Cas9 system in BT549 and MDA-MB-231 cells enhanced invasiveness (Fig. 3e, f). Colony formation ability was also increased in MDA-MB-231 cells when LacRNA was knocked out (Additional file 1: Fig. S4h). We confirmed that the positive function of LacRNA was not attributed to microRNAs in its introns (Additional file 1: Fig. S4g).

### LacRNA physically interacts with PHB2 protein and regulates its stability

To explore the molecular mechanisms underlying the biological activity of LacRNA in breast cancer, we used an RNA pulldown assay followed by mass spectrometry (MS) to identify the protein interactome of LacRNA. Briefly, biotinylated LacRNA sense and antisense transcripts (negative control), and co-precipitating proteins were isolated with streptavidin-agarose beads. Interestingly, the sense streptavidin labeled LacRNA, but not the antisense or beads only control, specifically precipitated with PHB2, a 32 kD protein^22^ (Additional file 1: Fig. S5a, d).

**Fig. 4:**
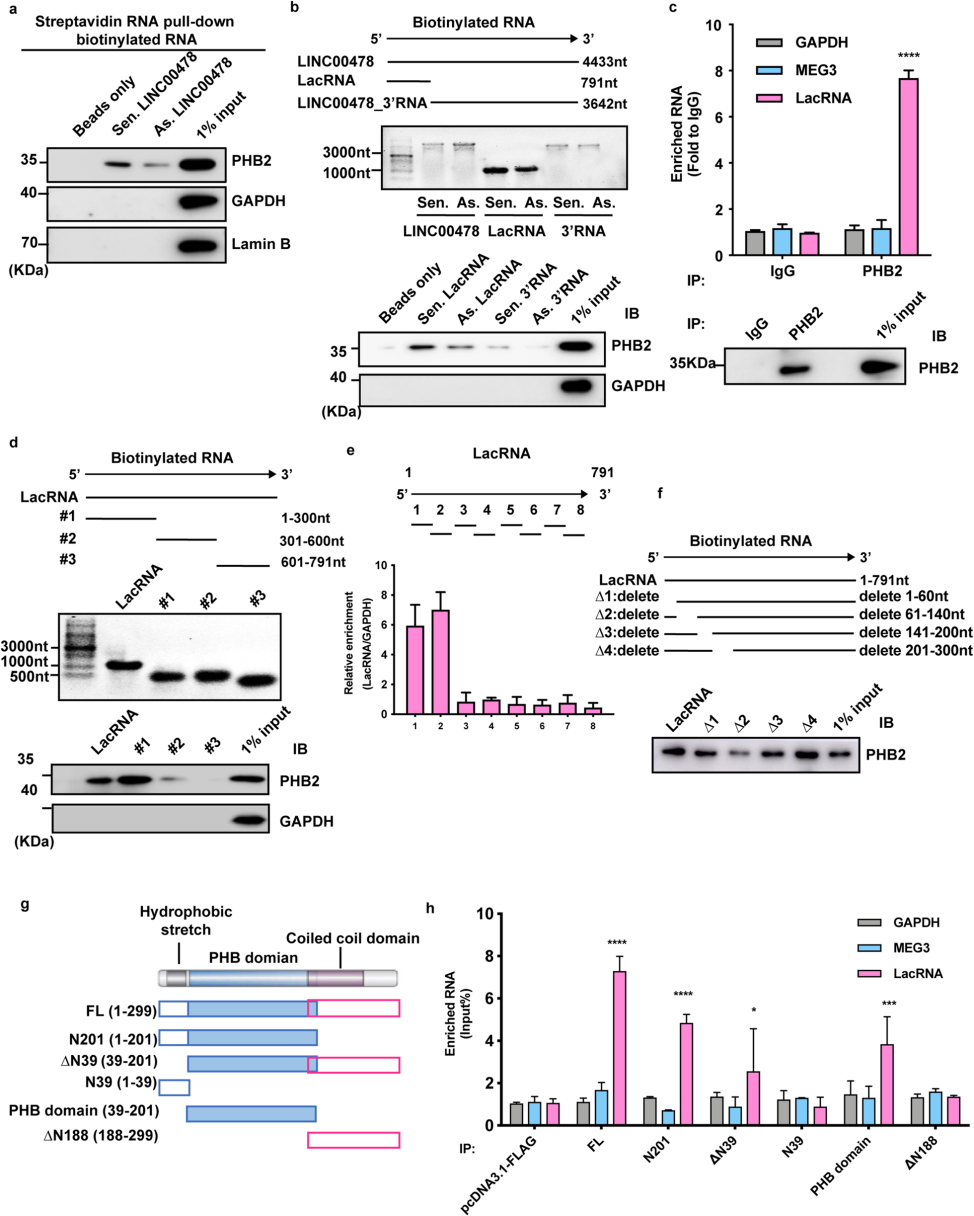
LacRNA physically interacts with PHB2 in breast cancer cells. **a** Full-length sense and antisense LINC00478 with a streptavidin RNA-pulldown assay and PHB2 immunoblotting. **b** Immunoblotting for PHB2 with biotinylated LacRNA and LINC00478_3′RNA using streptavidin RNA-pulldown assays. **c** Binding of LacRNA to the PHB2 complex shown by RNA-immunoprecipitation and qRT-PCR. MEG3 was used as a negative control. **d** Immunoblotting of PHB2 in pulldown samples by full-length biotinylated-LacRNA or truncated biotinylated- LacRNA motifs (#1: 1–300 nt; #2: 301–600 nt; #3: 601–791 nt;), with GAPDH as the negative control. **e** CLIP-qPCR analysis to map interaction between LacRNA and PHB2. Following the initial PAR-CLIP steps, partial digestion aimed at generating RNA fragments ranging 100–300 nt was performed using RNase T1. After IP using PHB2 antibody and extraction of RNA, PCR amplification was used to quantify the relative abundance of overlapping segments spanning LacRNA. **f** Immunoblotting of PHB2 in pulldown samples by full-length biotinylated LacRNA or biotinylated LacRNA with deleted motifs (△1: 1–60 nt deleted; △2: 61–140 nt deleted; △3: 141–200 nt deleted; △4: 201–300 nt deleted). **g**, **h** Truncation domains of PHB2 that bind to LacRNA according to RNA-immunoprecipitation and qRT-PCR. Values are expressed as the mean ± SEM, in c and h., *P* values were determined by Student's t-test. *P < 0.05, ***P < 0.001, ****P < 0.0001 versus IgG. IP, immunoprecipitation; IB, Immunoblotting. Uncropped images of the blots are shown in Additional file 1: Fig. S9

To verify the association between PHB2 and LacRNA, we analyzed lncRNA-pulldown protein samples by immunoblotting with a PHB2 antibody. Strong signals for PHB2 were observed in proteins pulled down with LINC00478_FL but not in proteins associated with either antisense LINC00478_FL or beads only (Fig. 4a), confirming that PHB2 is indeed specifically present in the LINC00478-associated protein complex. Then, we used biotinylated LacRNA and LINC00478_3′RNA to pull down proteins, and the results suggested that LacRNA was specifically associated with PHB2 (Fig. 4b).

To further confirm the interaction between LacRNA and PHB2, we performed an RNA-immunoprecipitation (RIP) assay in which the RNA-PHB2 complex was immunoprecipitated using a PHB2 specific antibody (Fig. 4c). Compared to the IgG-bound sample, the PHB2 antibody-bound complex had a significantly higher level of LacRNA. As a negative control GAPDH RNA and the unrelated lncRNA MEG3 were also quantified, and no significant enrichment of GAPDH and MEG3 was observed in the complex immunoprecipitated by the PHB2-specific antibody (Fig. 4c).

Using an RNA truncation assay based on the secondary structure of the LacRNA (Additional file 1: Fig. S5b), we found that the 1–300 nt motif of LacRNA is responsible for its association with PHB2 (Fig. 4d). We used cross-Linking Immunoprecipitation and qPCR (CLIP-qPCR) assay to map the interaction between LacRNA and PHB2. The results identified significant binding to fragments 1–2 of LacRNA (Fig. 4e). Furthermore, using a series of deletion mapping analyses, we identified the hairpin at nt 61–140 of the LacRNA mimic as the PHB2 binding motif that bonds to PHB2 (Fig. 4f, Additional file 1: Fig. S5c). Taken together, these results demonstrate that PHB2 is a LacRNA-associated protein.

**Fig. 5:**
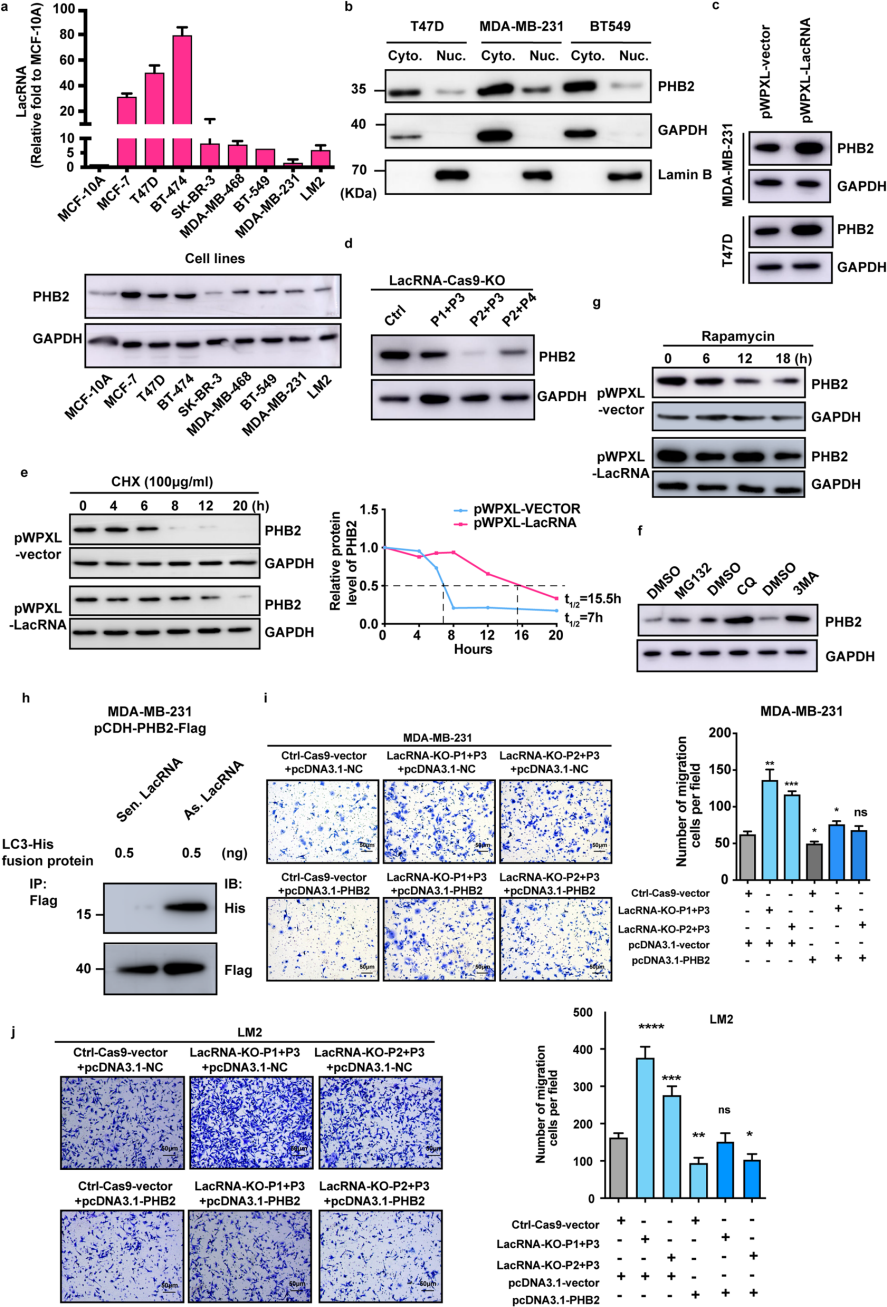
LacRNA regulates the stability of PHB2 protein. **a** RT-qPCR analysis of LacRNA expression and immunoblotting of PHB2 expression in 8 breast cancer cells and a normal breast cell line correspondently. The data are shown as mean ± SEM; n = 3 independent experiments. **b** The expression of PHB2 protein in the cytoplasmic and nuclear fractionations according to immunoblotting. **c** Immunoblotting of the expression of PHB2 in MDA-MB-231 and T47D cells transfected with empty vector (pWPXL-vector) or pWPXL-LacRNA. **d** Immunoblotting of the expression of PHB2 in MDA-MB-231 cells with LacRNA knockout by CRISPR/Cas9 technology. **e** Immunoblotting of the expression of PHB2 in MDA-MB-231 cells treated with the transcription inhibitor cycloheximide (CHX,100 μg/ml) for the indicated times and transfected with empty vector (pWPXL-vector) or pWPXL-LacRNA. **f** Immunoblotting for the protein levels of PHB2 in MDA-MB-231 cells with the proteasome inhibitor MG132 and autophagy inhibitors chloroquine (CQ) and 3-methyladenine (3MA) treated. **g** PHB2 autophagic degradation time in pWPXL-LacRNA and empty vector (pWPXL-vector) cells treated with the autophagy activator rapamycin for the indicated times. **h** Exogenous synthetic LC3-His protein was added to cell lysates of PHB2-FLAG protein-overexpressing cells (pCDH-PHB2-FLAG), and an immunoprecipitation assay with flag tag antibody was performed in the presence of sense or antisense LacRNA. Immunoblotting with a His tag antibody was used to test the immunoprecipitated LC3. **i** PHB2 rescue was performed after knockout of LacRNA in MDA-MB-231 cells, and transwell assays were used to assess cell migration. **j** Representative images of the transwell migration rescue assays in LacRNA knockout LM2 cells with additional PHB2 overexpressed. Mean ± SEM in **i** and **j**; n = 3 independent experiments; statistical significance was determined by one-way ANOVA. ns: not significant, *P < 0.05, **P < 0.01, ***P < 0.001. Uncropped images of the blots are shown in Additional file 1: Fig. S9

To define the domains in PHB2 that are responsible for its interaction with LacRNA, we generated a series of PHB2 truncated proteins (Fig. 4g). Based on the three domains of PHB2, including the hydrophobic domain, PHB, and coiled coil domains, we constructed a truncated PHB2 protein (Fig. 4g) [20]. An RNA-IP assay was used to further confirm the binding domain of PHB2 with LacRNA. As a result, the PHB domain was found to be the critical region for LacRNA binding (Fig. 4h). Surprisingly, PHB2 expression was positively correlated with LacRNA levels in breast cancer cells (Fig. 5a). LacRNA and PHB2 expression was much higher in three luminal type breast cancer lines with low metastatic potential (MCF7, T47D, and BT-474) than in five highly metastatic HER2-enriched and triple-negative breast cancer cell lines (SK-BR-3, MDA-MB-468, BT-549, MDA-MB-231, and LM2) (Fig. 5a).

The cytoplasmic localization of PHB2 implies that the protein may function by interacting with cytoplasmic LacRNA (Fig. 5b). The PHB2 mRNA level was not significantly affected when LacRNA was knocked out or overexpressed in MDA-MB-231 and T47D cells (Additional file 1: Fig. S6a, b). PHB2 protein levels were increased in MDA-MB-231 and T47D cells with ectopic LacRNA expression and decreased in MDA-MB-231 cells with LacRNA knock out (Fig. 5c, d). However, knockdown of PHB2 by siRNA did not influence the expression level of LacRNA (Additional file 1: Fig. S6c, d). The overexpressed PHB2-Flag fusion protein could be precipitated by streptavidin-labeled LacRNA sense strands in a dose-dependent manner (Additional file 1: Fig. S6e). Collectively, these data suggest that LacRNA physically interacts with the PHB2 PHB domain in the cytoplasm and enhances the expression of PHB2.

Thus, LacRNA could change the protein level of PHB2 in the cytoplasm of breast cancer cells, and we explored whether LacRNA could influence the protein stability of PHB2. We used cycloheximide (CHX) to inhibit protein synthesis, and found that overexpression of LacRNA could significantly prolong the half-life of the PHB2 protein, so LacRNA may play a role by stabilizing PHB2 (Fig. 5e). To further explore the specific mechanism by which LacRNA affects the stability of the PHB2 protein, breast cancer cells were treated with the proteasome inhibitor MG132 and the autophagy inhibitors chloroquine (CQ) and 3-methyladenine (3MA) (Fig. 5f). PHB2 was degraded mainly through the autophagy-lysosomal pathway, as CQ and 3MA significantly increased the level of the PHB2 protein (Fig. 5f). Moreover, when CQ was applied to block the autophagy pathway, the increase in PHB2 was time dependent (Additional file 1: Fig. S6f).

We used the autophagy activator rapamycin to treat cells, and overexpression of LacRNA was found to inhibit the rapamycin-induced autophagic degradation of PHB2 (Fig. 5g). Exogenous synthetic LC3-His protein was added to the cell lysates of PHB2-FLAG protein-overexpressing cells (pCDH-PHB2-FLAG), and an immunoprecipitation assay with a flag tag was performed in the presence of sense or antisense LacRNA. The data showed that the presence of LacRNA significantly affected the binding of PHB2 and LC3 and influenced the autophagy-mediated degradation process of PHB2 (Additional file 1: Fig. S6g, Fig. 5h). Therefore, LacRNA may inhibit the autophagic degradation of PHB2 protein by binding to its PHB domain.

We then clarified the function of PHB2 and the functional relationship between LacRNA and PHB2. The overexpression of PHB2 inhibited the migration of BT549 and MDA-MB-231 cells (Additional file 1: Fig. S6h, i). We knocked out LacRNA in MDA-MB-231 and LM2 cells and found that it significantly promoted cell migration. Moreover, when PHB2 was rescue overexpressed, reverses the effect of cell migration promotion (Fig. 5i, j). We rescue overexpress PHB2 in cells with LacRNA knocked out, and found the LacRNA in MDA-MB-231 and LM2 cells and found that it significantly promoted cell migration. Moreover, when PHB2 was rescue overexpressed, a decrease of cell migration promotion was observed (Fig. 5i, j).

All data above suggested that the cancer suppressive function of LacRNA is exerted through PHB2. LacRNA interacts with the PHB domain of the PHB2 protein through its 1–300 nt functional motif, influences the function of the autophagy degradation complex of PHB2 and LC3, and delays the degradation of the PHB2 protein.

### LacRNA-PHB2 complex is a negative regulator of MYC signaling

**Fig. 6:**
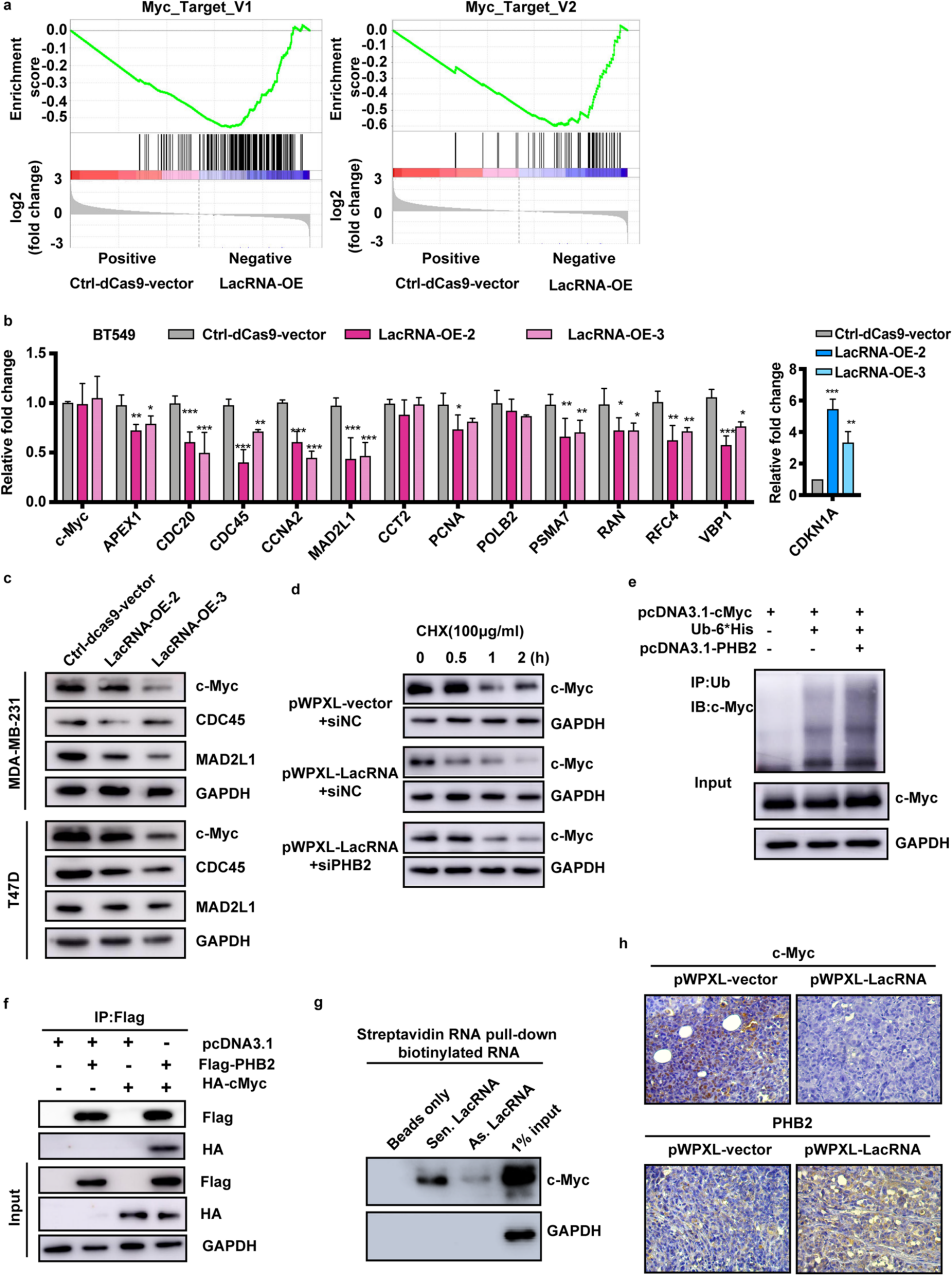
The LacRNA-PHB2 complex is a negative regulator of Myc signaling. **a** GSEA results of the Myc targets set with RNA-sequencing data after LacRNA activation. **b** RT-qPCR analyses of c-Myc and c-Myc target genes in BT549 cells with LacRNA activation by the CRISPR/dead-Cas9 technology. Mean ± SEM; n = 3 independent experiments. *P* values were determined by one-way ANOVA. *P < 0.05, **P < 0.01, ***P < 0.001. **c** Immunoblotting of the expression of c-Myc and c-Myc downstream genes (CDC45 and MAD2L1) in MDA-MB-231 and T47D cells with LacRNA activation by the CRISPR/dead-Cas9 technology. **d** Rescue CHX chase assays were conducted using MDA-MB-231 cells transfected with pWPXL-vector, pWPXL-LacRNA, and siPHB2 to measure c-Myc stability. Representative images of immunoblotting analyses examining c-Myc levels in MDA-MB-231 cells at various treatment time points are shown. **e** Cells were transfected with pcDNA3.1-PHB2 and His-tagged Ub plasmids for 48 h. The cell lysates were immunoprecipitated (IP) with either control IgG or His antibody and immunoblotted with c-Myc-specific antibody. c-Myc and GAPDH served as the loading controls. **f** Immunoprecipitation to detect the association between PHB2 and c-Myc. The pcDNA3.1-Flag-PHB2 plasmid and pcDNA3.1-HA-c-Myc plasmid were cotransfected into MDA-MB-231 cells. Immunoblotting of the Flag and HA tags. **g** Immunoblotting for c-Myc with biotinylated sense LacRNA and anti-sense-LacRNA using streptavidin RNA pulldown assays. **h** Representative data from immunohistochemistry staining of c-Myc and PHB2 in orthotopic transplanted tumors obtained from NOD/SCID mice after injection with vector pWPXL-LacRNA and control pWPXL-vector LM2 cells. Uncropped images of the blots are shown in Additional file 1: Fig. S9

Moreover, unbiased transcriptome profiling was performed using RNA-sequencing in LacRNA-activated cell lines using two independent gRNAs to explore which signaling pathways were regulated by LacRNA. The two LacRNA-activated cell lines showed similar expression trends for 1,002 genes, among which 200 genes were downregulated and 802 genes were upregulated (fold-change > 2, Additional file 1: Table S4). Gene set enrichment analysis (GSEA) analysis indicated that “MYC pathway/targets” were the prominent gene sets enriched by LacRNA (Fig. 6a). The two LacRNA-activated cell lines showed strong consistency (Additional file 1: Fig. S7a). RT-qPCR verified the results that activating LacRNA could lead to extensive inhibition of c-Myc downstream gene expression, but not *c-Myc* mRNA expression (Fig. 6b). We further investigated the changes in c-Myc protein levels after LacRNA activation. Immunoblotting assays showed that activation of LacRNA downregulated the expression of c-Myc protein, as well as its downstream targets CDC45 and MAD2L1 (Fig. 6c).

Deregulated expression of c-Myc has been observed in a wide variety of human cancers [21, 22]. c-Myc is a highly unstable protein that is usually degraded in less than 30 min in cells [23]. Reduction in c-Myc protein degradation results in the accumulation of c-Myc in many cancers, which may contribute to cancer development [24]. Most reported pathways that control c-Myc protein degradation are related to the ubiquitin-dependent proteasome pathway [23].

To determine whether LacRNA-PHB2 influences the stability of c-Myc, we measured the half-life of c-Myc in overexpressed LacRNA and wild-type (WT) MDA-MB-231 cells using a cycloheximide (CHX) chase assay (Fig. 6d). Overexpression of LacRNA decreased the half-life of c-Myc, as expected, compared with that in WT cells (Fig. 6d). Importantly, silencing of PHB2 in cells overexpressing LacRNA abolished the effect of LacRNA on c-Myc (Fig. 6d). These data suggest that a LacRNA-PHB2-independent pathway may exist to promote c-Myc degradation.

We validated whether alterations in the c-Myc expression level could affect LacRNA and PHB2. We overexpressed c-Myc in MDA-MB-231 cells, and immunoblotting and qRT-PCR showed that c-Myc overexpression did not change the expression of PHB2 and LacRNA (Additional file 1: Fig. S7b, c). Furthermore, after treating the cells with CQ, 3MA and MG132 (carbobenzoxy-l-leucyl-l-leucyl-l-leucinal), we found that MG132, a potent proteasome inhibitor, markedly increased the level of c-Myc levels (Additional file 1: Fig. S7d), indicating that c-Myc degradation is dependent on proteasomal activity.

To further validate the role of PHB2 in c-Myc degradation, we overexpressed PHB2 and demonstrated the promotion of c-Myc ubiquitination (Fig. 6e). We confirmed that c-Myc stability was regulated by PHB2. The physical interaction between c-Myc and PHB2 was determined using a co-immunoprecipitation (CO-IP) assay. We found that c-Myc was readily detected in PHB2 immunoprecipitants in breast cancer cells (Fig. 6f). The LacRNA RNA-pulldown assay confirmed that c-Myc could be enriched by sense LacRNA (Fig. 6g). To further explore the role of LacRNA in the interaction between PHB2 and c-Myc, cells overexpressing LacRNA were used for protein immunoprecipitation. It was preliminarily found that overexpression of LacRNA enhanced the interaction between PHB2 and c-Myc **(**Additional file 1: Fig. S7e). Therefore, it was speculated that LacRNA preferentially triggers PHB2:c-Myc heterodimer formation. These data indicate that the LacRNA-PHB2 complex interacts with c-Myc and promotes its ubiquitination and degradation.

*MYC*, an oncogene, is significantly amplified and highly expressed in breast cancer [25]. With an extremely high baseline expression level of c-Myc, we found that exogenous ectopic overexpression of c-Myc had no significant effect on breast cancer cell migration (Additional file 1: Fig. S7f). In contrast, LacRNA may significantly reduce the level of c-Myc protein through PHB2 and inhibit the migration and invasion of breast cancer cells (Additional file 1: Fig. S7g). In BT549 cells with overexpressed LacRNA, rescue over expression of c-Myc restored its migration function (Additional file 1: Fig. S7g). Taken together, these findings demonstrate that LacRNA forms a complex with PHB2 and directly acts with c-Myc, affecting the expression level of extensive Myc target genes. Thus, LacRNA exerts its tumor suppressor function by inhibiting c-Myc by stabilizing PHB2 in breast cancer cells.

We also assessed the levels of LacRNA, PHB2, and c-Myc, and their correlations in orthotopic transplanted tumors in mice. Tumors overexpressing LacRNA showed a higher level of PHB2 and a lower level of c-Myc than control tumors (Fig. 6h, Additional file 1: Fig. S7h). Immunofluorescence validated the colocalization of PHB2 and c-Myc (Additional file 1: Fig. S7i). In conclusion, the LacRNA-PHB2 complex could bind to c-Myc and induce its degradation which significantly attenuates the activation of c-Myc-specific target genes and then inhibits breast cancer metastasis.

### LacRNA-dependent c-Myc signaling downregulation correlates with improved survival in breast cancer

**Fig. 7:**
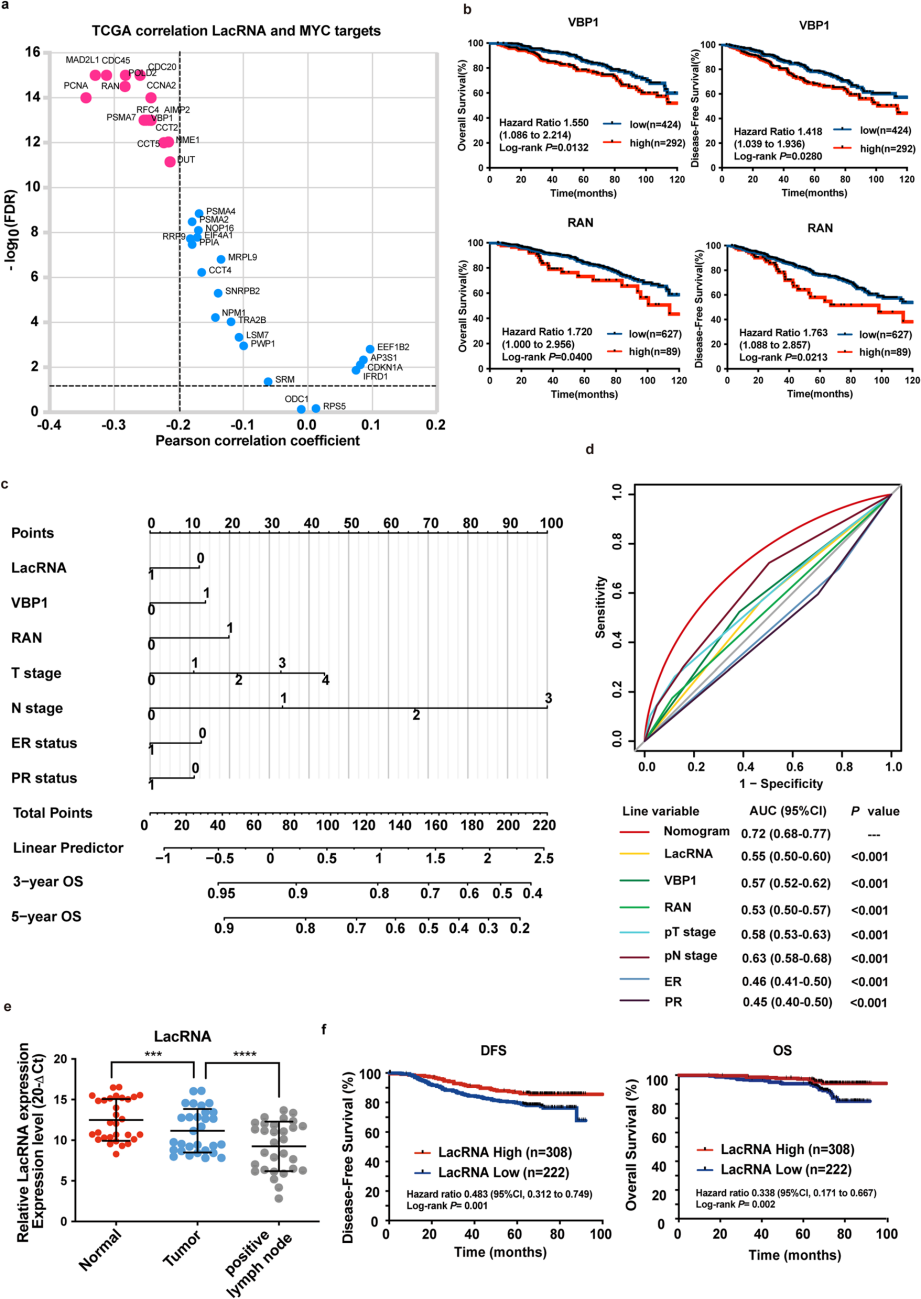
LacRNA-dependent c-Myc signaling downregulation correlates with improved survival in breast cancer. **a** Gene expression correlation analysis of c-Myc downstream pathways using through RNA-seq data of 1,094 breast cancer samples from the TCGA database. The correlations between LacRNA and Myc target expression levels were evaluated using the Pearson correlation coefficient test. Source data are available in Additional file 1: Table S5. **b** Kaplan–Meier analyses of the correlation between VBP1 and RAN levels and the disease-free survival and overall survival in 716 patients with breast cancer from TCGA database. **c** Constructed LacRNA and LacRNA-mediated c-Myc signaling downregulation nomogram to predict 3-year, 5-year overall survival for patients, with LacRNA, VBP1, RAN, T stage, N stage, ER status and PR status. **d** Comparisons of the prognostic accuracy by the nomogram, LacRNA and clinicopathological factors. **e **Scatter plots comparing LacRNA expression in paired adjacent normal breast tissues, breast cancer tissues and positive lymph node tissues (n = 30) by RT-qPCR. Statistical significance was determined by Kruskal–Wallis test., ***P < 0.001, ****P < 0.0001 **f **Kaplan–Meier analyses of the correlation between LacRNA levels and the disease-free survival and overall survival in 530 patients with breast cancer from FUSCC

Moreover, we validated the correlation between LacRNA and PHB2, c-Myc and its downstream pathways by analyzing RNA-seq data from 1094 patients with breast cancer from TCGA database. The results showed that there was no significant correlation between the expression levels of LacRNA and the mRNA levels of c-Myc and PHB2. While the mRNA levels of downstream target genes that were dependent on c-Myc activation were negatively correlated with LacRNA (Fig. 7a, Additional file 1: Fig. S8a, Additional file 1: Table S5).

To explore the clinical value of LacRNA and the LacRNA-mediated downregulation of c-Myc signaling in breast cancer we used data from 716 patients with breast cancer TCGA database, which excluded patients with stage IV disease and those censored within 12 months. Kaplan–Meier survival analyses showed that the high expression of VBP1 and RAN was associated with poor overall survival (OS) and disease-free survival (DFS) (Fig. 7b). Then, a prognostic nomogram was constructed that integrated LacRNA, LacRNA-mediated c-Myc signaling, and clinicopathological risk factors (Fig. 7c). To further assess the cumulative effects of the nomogram on risk score prediction, we calculated the area under the receiver operating characteristic curve (AUC). Receiver operating characteristic (ROC) analysis showed that the AUC for OS of nomogram was 0.72 (95% CI 0.68–0.711), which is much higher when compared to any of the single gene and clinicopathological factors (Fig. 7d).

We collected paired normal breast, breast cancer, and positive lymph node tissues from 30 patients with breast cancer who underwent surgery at the FUDAN University Shanghai Cancer Center (FUSCC). LacRNA expression was significantly lower in breast cancer tissues than in normal tissues and was the lowest in positive lymph node tissues (Fig. 7e).

To further investigate the value of LacRNA in predicting the prognosis of breast cancer, we collected tumor tissues from 530 consecutive breast cancer patient’s cohort who underwent surgery at FUSCC. RNA was extracted from tissues, and RT-qPCR was used to determine the expression of LacRNA. Kaplan–Meier survival analyses showed that a high expression level of LacRNA was associated with better OS (HR = 0.338, 95% CI 0.171–0.667, *p* = 0.002) and DFS (HR = 0.483, 95% CI 0.312–0.749, *p* = 0.001) (Fig. 7f). The association between LacRNA and improved survival in patients with breast cancer remained significant after adjusting for cancer subtypes along with other prognostic factors including age, stage, grade, and molecular subtype (multivariate Cox regression model, DFS: HR = 0.483,95%CI 0.287–0.811, *p* = 0.006, OS: HR = 0.318, 95% CI 0.135–0.750, *p* = 0.009) (Additional file 1: Table S6).

**Fig. 8:**
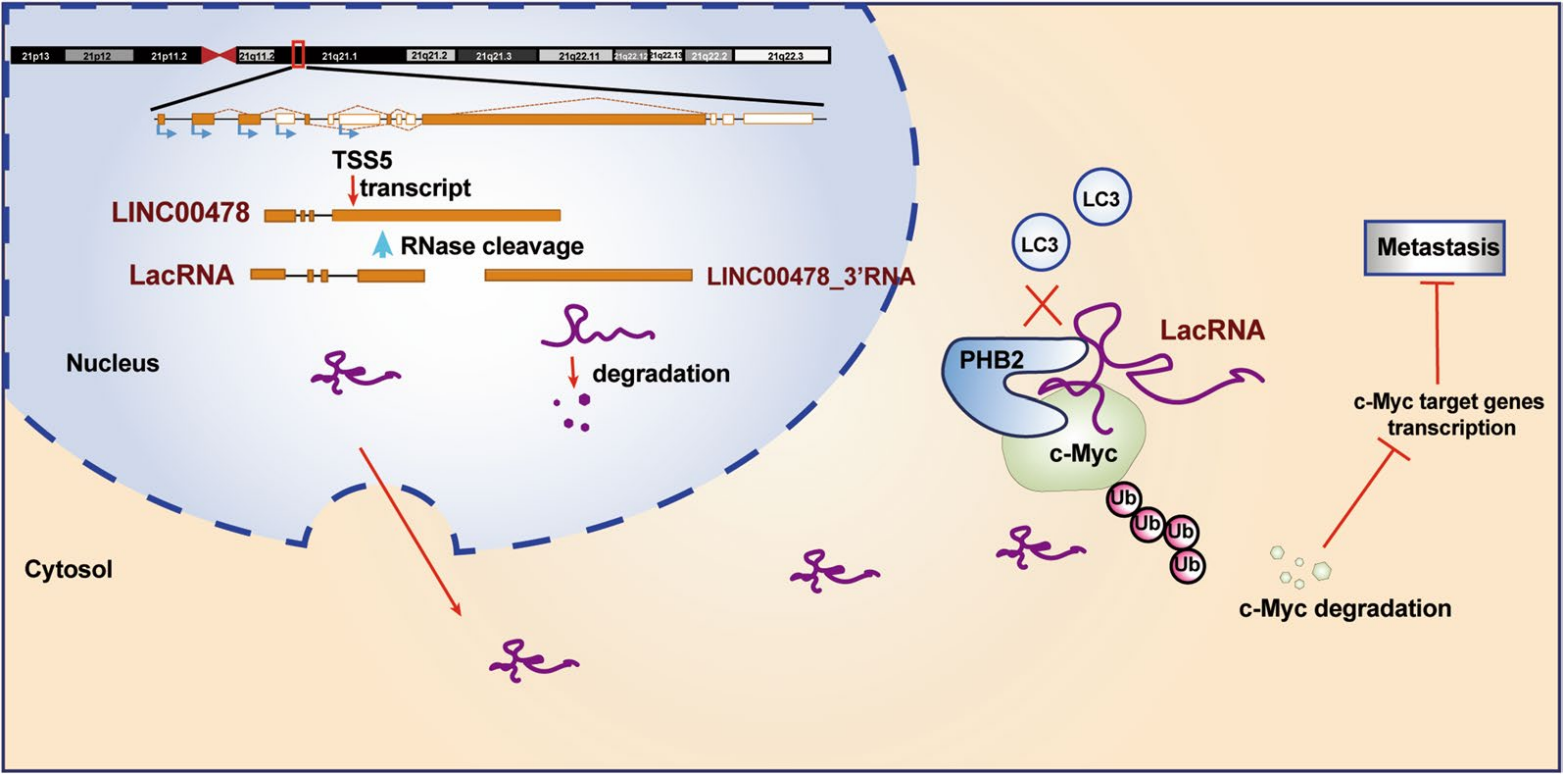
Schematic diagram. Schematic diagram of LacRNA-PHB2-c-Myc axis regulating breast cancer metastasis

Taken together, our results suggest that LacRNA plays an important role in breast cancer metastasis and may be a potential prognostic biomarker and a new therapeutic strategy for breast cancer (Fig. 8).

## Discussion

The development of precise and individualized treatment significantly improved the survival of patients with breast cancer. However, metastasis is still the primary cause of breast cancer-related death worldwide. Therefore, investigating the molecular mechanism regulating cancer metastasis and progression may provide helpful insights into the development of efficient diagnostic and therapeutic strategies. Recent studies have indicated that lncRNAs play critical roles in breast cancer metastasis [11, 26]. In this study, we identified six downregulated lncRNAs in metastatic lymph node tissues compared with primary tumor tissues from patients with breast cancer. Among them, MEG3, a well-known anticancer lncRNA, has been reported in multiple studies [27, 28]. Among the six lncRNAs, LINC00478 displayed a remarkable trend of downregulation in breast cancer tissues, whereas its high expression was associated with a better prognosis in patients with breast cancer. All these results preliminarily indicated that LINC00478 might play an important role in inhibiting breast cancer. Several high-throughput sequencing studies screened the differentially expressed lncRNAs between cancer and adjacent normal tissues. LINC00478 is reportedly significantly upregulated in normal tissues compared to cancer tissues and upregulated in early stages and better differentiation tumors [29–31]. However, none of these studies explored the molecular mechanism in depth.

To form mature and functional lncRNAs, extensive transcriptional and posttranscriptional processing take place, yielding a great number of isoforms [32, 33]. A recent study suggested that over 40% of long transcripts may function as precursors for small RNAs of less than 200 nt in length [14]. Similar to metastasis-associated lung adenocarcinoma transcript 1 (MALAT1) (~ 7-kb) [34, 35], LINC00478 is a long lncRNA with complex transcripts. The full-length LINC00478 is ~ 4548 nucleotides with 12 main alternative splicing variants. In this study, we innovatively identified the mature and functional form of LINC00478. The isoform NR_136544 was verified to be mainly expressed in breast cancer cells. Then we demonstrated that processing LINC00478 (NR_136544) nascent transcript yields mature 5′ ends of cytoplasmic RNA and 3′ ends of nuclear-retained RNA. We named the 5′ end 817-nt RNA as LacRNA and the 3′ end of nuclear RNA LINC00478_3′RNA. The post-transcriptional processing of LINC00478 is similar to that of MALAT1 [36]. MALAT1 is reportedly to be cleaved to simultaneously generate the 3′ end of MALAT1-associated small cytoplasmic RNA (mascRNA) and the 5′ end of the nuclear-retained RNA [36]. Unlike MALAT1, both MALAT1 and mascRNA are abundantly expressed and have cancer-promoting proportions; however, almost all LINC00478_FL RNA was cleaved after transcription. Of the cleaved products, LacRNA was more stable than LINC00478_3′RNA. Moreover, LacRNA but not LINC00478_FL and LINC00478_3′RNA showed strong activity inhibiting breast cancer metastasis in vitro and in vivo. Of note, we validated that the cleaved short LacRNA is the primary functional form of LINC00478 in breast cancer progression.

Although lncRNAs are more likely than mRNAs to be localized in the nucleus, studies have shown that the number of lncRNA transcripts in the cytoplasm exceeds that in the nucleus [37]. LacRNA belongs to the lncRNA type significantly enriched in the cytoplasm, where it exerts its functions. lncRNAs in the cytoplasm typically exert their biological functions mainly through physical interactions with regulatory proteins [11, 38, 39]. In this study, we identified PHB2 as the *bona fide* interacting partner of LacRNA, and the LacRNA-PHB2 complex was found to exert an inhibitory effect on breast cancer metastasis through c-Myc.

PHB, identified while screening for antiproliferation-related genes, was termed prohibitin (PHB) [40]. PHB2 exerts broad biological activities and has been reported as a critical mediator of cancer-associated signaling pathways in multiple cancers [41, 42]. PHB2 binds to the 61–140-nt region of the LacRNA 5′ terminus with the PHB domain. The PHB domain has been proven to be a key domain in protein–protein interactions [43], and we suggest that this domain might also be pivotal in interacting with RNA. PHB2 is degraded mainly through the autophagy-lysosomal pathway. A recent study found that the autophagy recognition protein LC3 on the surface of autophagic vesicles can directly bind to the PHB2 protein in the inner mitochondrial membrane and undergo autophagic degradation [44]. The binding of LC3 to the PHB2 protein is mediated by the PHB domain, which is consistent with the LacRNA binding region. We demonstrated that the exits of LacRNA could block PHB2-LC3 immunoprecipitation and influence the degradation of PHB2.

Furthermore, the LacRNA-PHB2 complex directly binds to c-Myc and promotes its degradation, ultimately triggers the inhibition of Myc signaling. LacRNA played a critical role in the LacRNA-PHB2-cMyc axis. *Myc* has been widely reported as an oncogene and is seemingly the core of the malignant tumor regulatory networks [45]. It was found that 15% of the gene promoter region was widely bound by Myc [46]. Thus, it is reasonable to explain the extensive inhibition of Myc target genes and the notable tumor-suppressive function of LacRNA in breast cancer.

Importantly, higher LacRNA levels predicted significantly better DFS and OS in patients with breast cancer, supporting that LacRNA may be a promising prognostic biomarker for breast cancer. Moreover, based on LacRNA and LacRNA-mediated c-Myc signaling, we constructed a model for predicting the survival of patients with breast cancer, which outperformed other clinicopathological risk factors. Overall, the above findings suggest that LacRNA could be a potential new therapeutic method and may facilitating the development of precise diagnosis and treatment strategies for breast cancer.

## Methods

### Patients and ethical statement

Paired primary breast tumor and metastasis lymph node were obtained from the department of breast surgery of Fudan University Shanghai Cancer Center (FUSCC), Shanghai, China. A total of 105 paired primary breast cancer and adjacent normal tissues from TCGA database (https://cancergenome.nih.gov/). The 827 cases of breast cancer patients' RNA-seq data and OS data were come from TCGA database. 530 cases of breast cancer tissues and 98 cases of patients' plasma were obtained from FUSCC. The use of human clinical specimens in the present study was approved by the Medical Ethics Committee of Fudan University Shanghai Cancer Center. The patients were informed, and they signed consent forms acknowledging the use of their resected tissues for research purposes.

### Microarray analysis

Five pairs of primary breast cancer tissues and axillary lymph node metastatic loci were dissected and verified by H&E staining, tumor percentage > 70%. RNAs were extraction with TRIzol reagent (Life technologies, Carlsbad, CA, USA) according to the manufacturer’s protocol. Samples were analyzed with Affymetrix HTA 2.0 by Shanghai Qiming Biotechnology Co. Ltd.

### Cell lines and cell culture conditions

Cell lines used were authenticated by short tandem repeats (STR) profiling. All cell lines were confirmed to be free of mycoplasma (Vazyme). All the cell lines were maintained in Dulbecco's modified Eagle's medium (DMEM) medium (Hyclone, Thermo Fisher Scientific, USA) containing 10% fetal bovine serum (FBS) (Gibco, Thermo Fisher Scientific, USA), 100 IU/ml penicillin and 100 μg/ml streptomycin (Invitrogen, Carlsbad, CA, USA) in a 37 °C incubator with 5% CO2. LM2 was a subline of MDA- MB-231 with high lung metastasis tendency was described previously [47]. Cycloheximide (CHX), proteasome inhibitor MG132, autophagy inhibitors chloroquine (CQ) and 3-methyladenine (3MA) were purchased from Sigma-Aldrich.

### Paired-guide RNA CRISPR–Cas9 KO and CRISPR/dead-Cas9 induction generation

The desired Cas9 cutting site in the LacRNA promoter genomic region were selected, and the target sequences in the region around 100~200 bp flanking the site were searched in http://www.addgene.org. The single guide RNA (sgRNA) was designed and the sequences of high-scored sgRNAs are presented in Additional file 1: Table S2. Using paired-gRNA CRISPR/Cas9 KO system to generate a large genomic deletion to investigate the function of LacRNA [18, 19]. The four candidate sequences (sgRNA#1-#4) were cloned into paired-gRNA-Cas9 vector (Additional file 1: Fig. S4a, b). For the CRISPR-Cas9 KO system, the lentivirus was collected 48 h after transfection of the paired-gRNA-Cas9 vector into HEK293T cells using the Lipofectamine 2000 transfection reagent (Invitrogen, Carlsbad, CA, USA). For the CRISPR/dCas9 activation system, the co-transfected plasmids were the lenti-gRNA, lenti-dCas9-vp64-blast and lenti-MS2- p65-HSF1 vector. The plasmids used in this study were purchased from Addgene (Cambridge, MA, USA). The target cells were infected with filtered lentivirus plus 6 μg/ml polybrene (Sigma-Aldrich) for 24 h and treated with 5 μg/ml Blasticidin (Solarbio), 600 μg/ml hygromycin (Solarbio) and 400 μg/ml zeocin (Thermo) for more than 7 days to obtain selective antibiotic markers before initiating the experiment. The stably transfected cells established via the CRISPR/Cas9 knockout and CRISPR/dead-Cas9 activation technology was nominated as KO and OE, respectively. These stable cell lines were all polled clones. All the cell lines were used within 20 passages and thawed fresh every 2 months.

### Stable and transient overexpression

The human LINC00478_FL, LacRNA and LINC00478 3′RNA were cloned into the transient expression vector pcDNA3.1 and lentivirus stable expression vector pWPXL to be overexpressed. The PHB2 and c-Myc expression vectors were constructed by inserting the respective open-reading frame sequences into the pcDNA3.1-FLAG vector to generate pcDNA3.1-PHB2-FLAG and pcDNA3.1-c-Myc-FLAG respectively. Additionally, we cloned the sequence of c-Myc into the pcDNA3.1-HA vector to generate pcDNA3.1-c-Myc-HA. The PCR primers are listed in Additional file 1: Table S2. The vector pcDNA3.1 with each sequence inserted was transfected into breast cancer cells using the Lipofectamine 3000 transfection reagent (Invitrogen, Carlsbad, CA, USA). The HEK293T cells were transfected with pWPXL-LINC00478_FL, pWPXL-LacRNA or pWPX-LINC00478 3′RNA, with the packaging and envelope plasmids psPAX2 and pMD2.G, respectively, according to the manufacturer's instructions. The virus particles were harvested by passing through a 0.45 mm filter 48 h after transfection. The breast cancer cells were infected with recombinant lentivirus transducing with the addition of 8 mg/ml polybrene (Sigma-Aldrich). siRNA transfection was performed using Lipofectamine RNAiMAX (Life technologies, Carlsbad, CA, USA). Sequences of PCR primers and siRNAs were listed in Additional file 1: Table S2.

### Reverse transcription PCR and quantitative real-time PCR

RNA samples from the clinical tissue specimens and cell lines used in this study were extracted with TRIzol® reagent (Life technologies, Carlsbad, CA, USA) according to the manufacturer’s protocol. First-strand cDNA was synthesized using the Hiscript III Reverse Transcriptase (Vazyme). Relative RNA levels determined by quantitative Real-Time PCR (qPCR) were measured on a 7900 Real-Time PCR System with the SDS 2.3 software sequence detection system (Applied Biosystems, USA) using the ChamQ SYBR qPCR Master Mix (Vazyme) method. The sequences for the gene-specific primers used are listed in Additional file 1: Table S2. GAPDH was employed as an internal control to quantify of LacRNA and the mRNA levels of other genes. The relative levels of RNA were calculated using the comparative CT (2^−ΔΔCT^) method.

### 5′ and 3′ RACE assay

We used′ 5′- and 3′-rapid amplification of cDNA ends (RACE) assay to determine the transcriptional initiation and termination sites of LacRNA with a SMARTer RACE cDNA Amplification kit (Clontech), following the manufacturer’s instruction. The sequences for the gene-specific PCR primers used for 5′ and 3′ RACE analysis are given in Additional file 1: Table S2.

### Subcellular fractionation

Cytoplasmic and nuclear RNA fractions of the MDA-MB-231, BT549 and T47D cells were prepared and collected according to the instructions of the PARIS™ RNA Nuclear/ Cytoplasmic Isolation kit (Life technologies, Carlsbad, CA, USA). Cytoplasmic and nuclear protein fractions of the MDA-MB-231, BT549 and T47D cells were prepared and collected according to the instructions of the NE-PERTM Nuclear Cytoplasmic Extraction Reagent (Life technologies, Carlsbad, CA, USA). β-actin was used as the cytoplasmic endogenous control. U6 small nuclear RNA was used as the nuclear endogenous control. GAPDH was used as the cytoplasmic protein control. Lamin B was used as the nuclear protein control.

### Transwell and wound healing assays

For transwell migration and invasion assays, cells in each group were digested, centrifuged and resuspended with serum-free medium, and the cells were counted. The cell density was adjusted to 2 × 10^5^/ml for migration assay and 4 × 10^5^/ml for invasion assay. The lower chambers of 24-well plate-sized transwell inserts (Corning Falcon) contained 600 μl DMEM with 20% FBS for migration assay and 30% FBS for invasion assay. Blow and mix the cell suspension and seeded 200 μl on the top chamber. Incubation for 8–12 h, different culture time was set according to the migration and invasion ability of different cell lines, and the invasion experiment time was appropriately prolonged. After incubating, the inserts were fixed and stained with crystal violet. Cells and dye on the bottom of upper chamber were gently wiped off with cotton swabs. The average confluence of migrated cells was analyzed by ImageJ according to three random fields captured by 100× microscope. Each experiment was conducted in triplicate.

For wound healing assay, cells were resuspended and adjusted to 1.8 × 10^5^/ml. 200 μl cell suspensions were seeded in 96-well IncuCyte® ImageLock Plates (Essen Bioscience). After 24 h, a wound was made in each well with WoundMaker™. Plates were imaged as described above with 4-h interval. Wound width was measured and analyzed.

### In vivo assays

Female NOD/SCID mice, aged 4–6 weeks old, purchased from the Shanghai Slack Laboratory Animal Co., LTD and housed under SPF conditions at the animal care facility of the Experimental Animal Center of Shanghai University of Traditional Chinese Medicine.

For xenograft models, 200 μl cell suspension containing 2 × 10^6^ cells (pWPXL-VECTOR and pWPXL-LacRNA stable MDA-MB-231-LM2 cell line) were orthotopically injected directly into the inguinal mammary fat pads of mice. Tumor growth rates and mice body weight were monitored. When a tumor was palpable, it was measured every other day, and its volume was calculated according to the formula volume = length × width × 0.5. After 7 weeks, the tumor in situ was removed and divided into 3 parts: 4% formaldehyde fixation, RNA later preservation and direct freezing in − 80 ℃. After resection of the tumor in situ, the prepared fluorescent substrate dissolved with DPBs to a concentration of 20 mg/ml was injected immediately, and each mouse was injected with 200 μl from the caudal vein. Imaging was performed immediately in a small animal in vivo imaging system. Then, the mouse lung tissue was removed, imaged again, and the lung tissue was preserved.

To further investigate the effect of LacRNA on tumor invasion in vivo, we developed the metastasis model in female athymic BALB/c nude mice. Using the LacRNA activated stable MDA-MB-LM2 cell lines, 200 μl cell suspension containing 2 × 10^5^ cells (Ctrl-dCas9-vector and LacRNA-OE3) were injected into the caudal vein, and the fluorescent imaging was performed after 5 weeks. Mice were killed and the number of metastatic foci in the lung were calculated. All experiments were performed in accordance with relevant institutional and national guidelines and regulations of Shanghai Medical Experimental Animal Care Commission.

### In vitro transcription, RNA-pulldown assay and mass spectrometry analysis

PCR products were verified by gel electrophoresis and purified by phenol: chloroform extraction. In vitro transcription of LINC00478_FL, LacRNA, LINC00478 3′RNA and different truncated LacRNA was conducted using a HiScribe™ T7 Quick High Yield RNA Synthesis Kit (NEB) with Biotin-16-UTP (Roche). Briefly, the HIScribe T7 Transcription system is completed in a single-tube and transcription reactions expression of genes cloned downstream from the T7 RNA polymerase. 1.0 μg of circular plasmid DNA is added to an aliquot of the Master Mix and incubated in a 40 μl reaction volume for 4 h at 37 °C. Transcription RNA was treated with RNase-free DNase I on column during RNA purification with RNA Clean & Concentrator-25 (Zymo Research). Transcribed and labeled RNA was purified with a Direct-zolTM RNA Miniprep Plus Kit (ZYMO). Next, 1 pmol biotinylated RNA was pretreated with RNA structure buffer (Beyotime Biotechnology, Shanghai, China) to obtain an appropriate secondary structure formation.

For each sample, 3 μg pretreated biotinylated RNAs were incubate with 2 mg protein and rotated overnight at 4 ℃. Then gently mixed with 50 μl washed Dynabeads™ M-280 Streptavidin (11206D) (Thermo Scientific) and incubated on a rotator at 4 ℃ for 2 h. The beads were washed briefly five times in RIPA buffer (10 mM Tris–HCl, 150 mM NaCl, 1% Nonidet P-40, 1 mM EDTA, 0.1% SDS and 1 mM DTT). The protein complexes were eluted, denatured, and digested with immobilized trypsin (Promega) for mass spectrometry analysis (Shanghai Applied Protein Technology, Shanghai, China). The retrieved protein was detected by immunoblotting. The primers for PCR and its deletion and mutation fragments for in vitro transcription are provided in Additional file 1: Table S2.

### RNA-immunoprecipitation (RIP) assay

Cells growing in 10 cm-dishes were lysed in 0.5 ml of lysis buffer containing protease inhibitors and RNase Inhibitor (Thermo Fisher Scientific Inc., Rockford, IL, USA) and centrifuged at 12,000 rpm for 5 min. Suspension 1 × 10^7^ cells in RIP lysis buffer. Ultrasonic at 4 ℃ for 3 × 10 s, intermittent for 1 min, then centrifuged at 15,000×*g*, 4 ℃ for 5 min. Leave 50 μL stored at − 80 ℃ as input. The supernatants were incubated with 15 μl Pierce™ Protein G Dynabeads (Thermo Fisher Scientific) for pre-clear. The precleared lysates were incubated with 5 μg indicated antibodies for 12 h at 4 °C with gentle rotation. Then 50 μl Dynabeads Protein G beads (blocked with 1% BSA and 20 mg/ml yeast tRNA for 1 h at 4 °C) were added to the mixture and incubated for another 2 h at 4 °C. The beads were washed thrice with wash buffer containing RNase inhibitor. The RNA–protein complex was eluted with 200 μl TRIzol at room temperature for 10 min. RNA was purified with Direct-zol RNA Microprep (Zymo Research). For qRT-PCR, reverse transcription was performed with Hiscript III Reverse Transcriptase (Vazyme) followed by qRT- PCR analysis. Information on the antibodies are listed in Additional file 1: Table S3.

### Immunoblotting analysis

Cells were lysed for 30 min in ice with lysis buffer (Beyotime Biotechnology) containing protease inhibitors and phosphatase inhibitors (Bimake). After centrifugation at 16,000×*g* for 15 min at 4 °C, the protein concentrations were determined by the BCA method (Pierce, Thermo Fisher Scientific Inc., Rockford, IL, USA). Add the proteins with 5 × loading buffer and use 1 × loading buffer to make sure all the samples the same concentration. Heating samples at 100 °C for 10 min for denaturation. The samples were resolved by SDS/PAGE, transferred to PVDF membranes (Millipore, Massachusetts, USA). The membranes were blocked with 5% (w/v) skimmed milk in TBS at room temperature for 1 h, then probing with antibodies at 4 °C overnight. Using immune blotting to analysis by HRP-conjugated secondary antibodies. Information on the antibodies is provided in Additional file 1: Table S3. A chemiluminescent (ECL) chromogenic substrate was used to visualize the bands (Solarbio). Visualization was performed using the hemic Doc™ XRS Imager (Bio-Rad, USA). The uncropped blots are shown in Additional file 1: Fig. S9.

### Cross-linking immunoprecipitation and qPCR (CLIP-qPCR)

CLIP-qPCR (cross-linking and immunoprecipitation followed by reverse transcription and quantitative PCR) was used to mapping and quantifying PHB2-lacRNA interactions. MDA-MB-231 cells were incubated with DMED culture medium eith 4-Thiouridine (4-SU) added (1 M stock solution in DMSO, to a final concentration of 100 μ min). Irradiate cells with 150 mJ/cm2 of UVA (365 nm), then scrape cells in 5 ml PBS, transfer to 50 ml conical tube, centrifuge at 2000× *g* at 4 ℃ for 5 min and aspirate the supernatant. Lysis cells in NP-40 buffer and incubate with RNase T. RNA was extracted and purified. Run the RNA samples in 1.5% formaldehyde agarose gel to verify that RNAs are digested in 100- to 300-nt range. The supernatants were incubated with 5 μg PHB2 antibody for 12 h at 4 °C with gentle rotation. Then 50 μl Dynabeads Protein G beads were added to the mixture and incubated for another 2 h at 4 °C. After centrifugation at 2000×*g* for 1 min at 4 °C, wash beads three times with NP-40 lysis buffer. Incubate the pellets with 20 units of RNase-free DNase I in 100 μl NP-40 lysis buffer for 15 min at 37 °C. Then extracted and purify RNA. For qRT-PCR, reverse transcription was performed with Hiscript III Reverse Transcriptase (Vazyme) followed by qRT- PCR analysis.

### Statistical analysis

The experiment was set up to use 3–5 samples/repeats per experiment/group/condition to detect a difference at the significance level of 0.05 by a two-sided test for significant studies. For RNAscope, northern blotting, RACE analysis, transwell assay, wound healing assay, immunoblotting and immunohistochemical staining, representative images are shown. Data were presented as the mean ± standard error of the mean (SEM) or of at least three independent experiments. Each exact n values are indicated in the corresponding figure legend. Student's *t*-test or one way analysis of variance (ANOVA) were performed to evaluate the differences between two groups or more than two groups, as indicated in the individual Figures. Wilcoxon tests were used to analyze RNA levels in paired cancer and normal samples, and Mann–Whitney tests were used to analyze LacRNA levels in grouped human samples. The univariate and multifactorial Cox regression models were used to determine the independent clinical factors based on the investigated variables. The Kaplan–Meier survival analyses the correlation between the LacRNA levels and survival. Pearson's correlation was performed to analyze the correlation of LacRNA and PHB2, c-Myc and Myc target genes expression. All statistical analyses were performed using SPSS v.23.0 (Armonk, NY, USA) and GraphPad Prism 8.0.

## Supplementary Information

**Additional file 1.** Additional Tables and Figures.

## Data Availability

The datasets used and/or analyzed during the current study are available from the corresponding author on reasonable request.
