# Supplementary material for: LINC00478-derived novel cytoplasmic lncRNA LacRNA stabilizes PHB2 and suppresses breast cancer metastasis via repressing MYC targets
Source: J Transl Med. 2023 Feb 13;21:120. doi: 10.1186/s12967-023-03967-1 (PMC9926633; doi:10.1186/s12967-023-03967-1)
Supplement: Supplementary file 1 — Additional file 1. Additional Tables and Figures. [file 12967_2023_3967_MOESM1_ESM.pdf]

Additional Figures

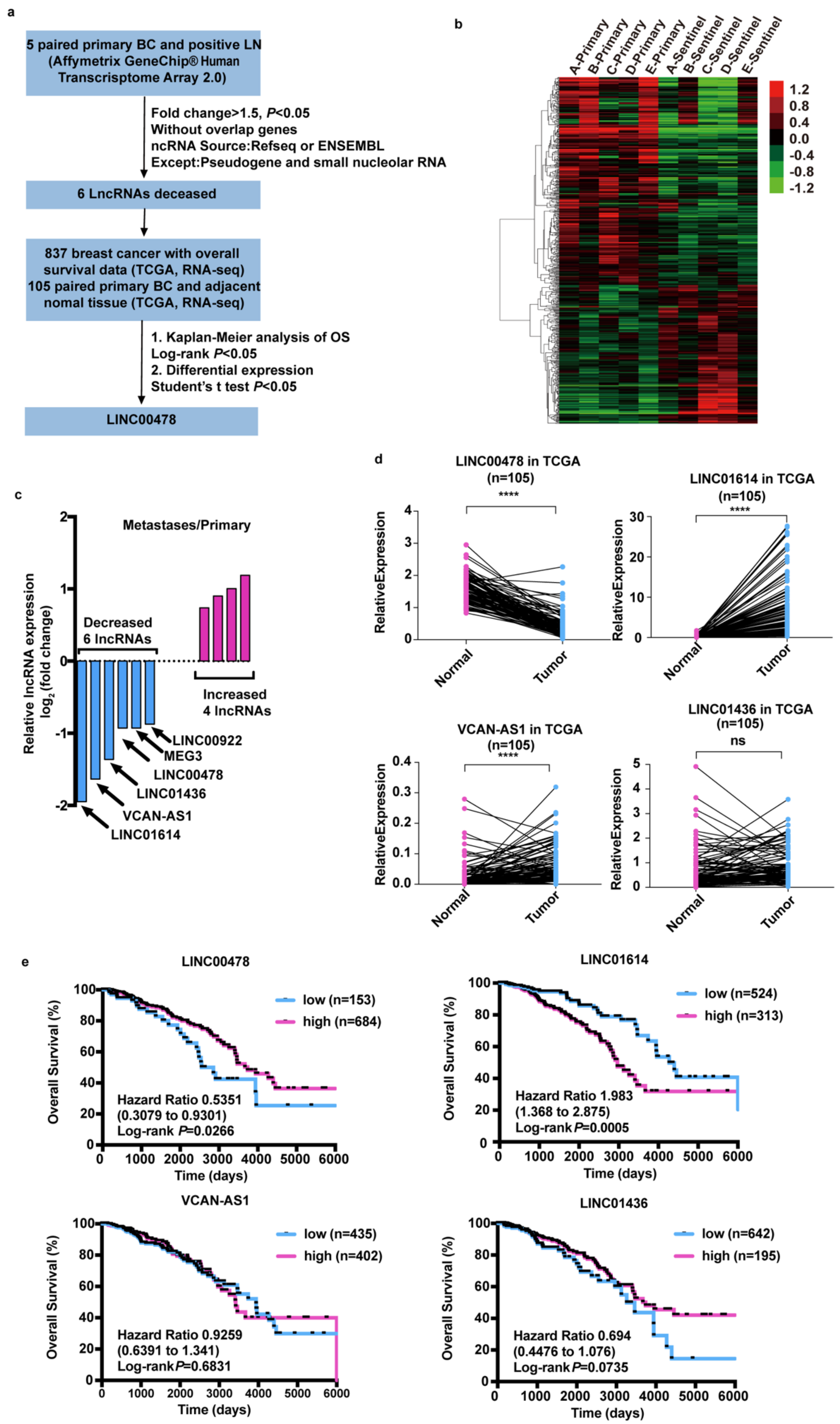

Figure S1. The screening of lncRNAs associated with breast cancer progress.

(a) Schematic flow chart for screening lncRNAs associated with breast cancer progression. **(b)** Heat map of RNA microarray data showing different expression RNA between primary breast tumors and paired axillary lymph node metastatic loci. **(c)** Microarray data showing the relative expression of candidate lncRNAs differentially expressed in metastatic tissues and primary tumor tissues. **(d)** TCGA database of LINC00478, LINC01614, VCAN-AS1 and LINC01436 relative expression in 105 paired primary breast cancer and adjacent normal tissues. *P* values were determined by Mann-Whitney test. ns, not significant, ****p<0.0001. **(e)** TCGA database of LINC01614, VCAN-AS1 and LINC01436 expression and overall survival in 837 breast cancer patients (Kaplan-Meier log rank test).

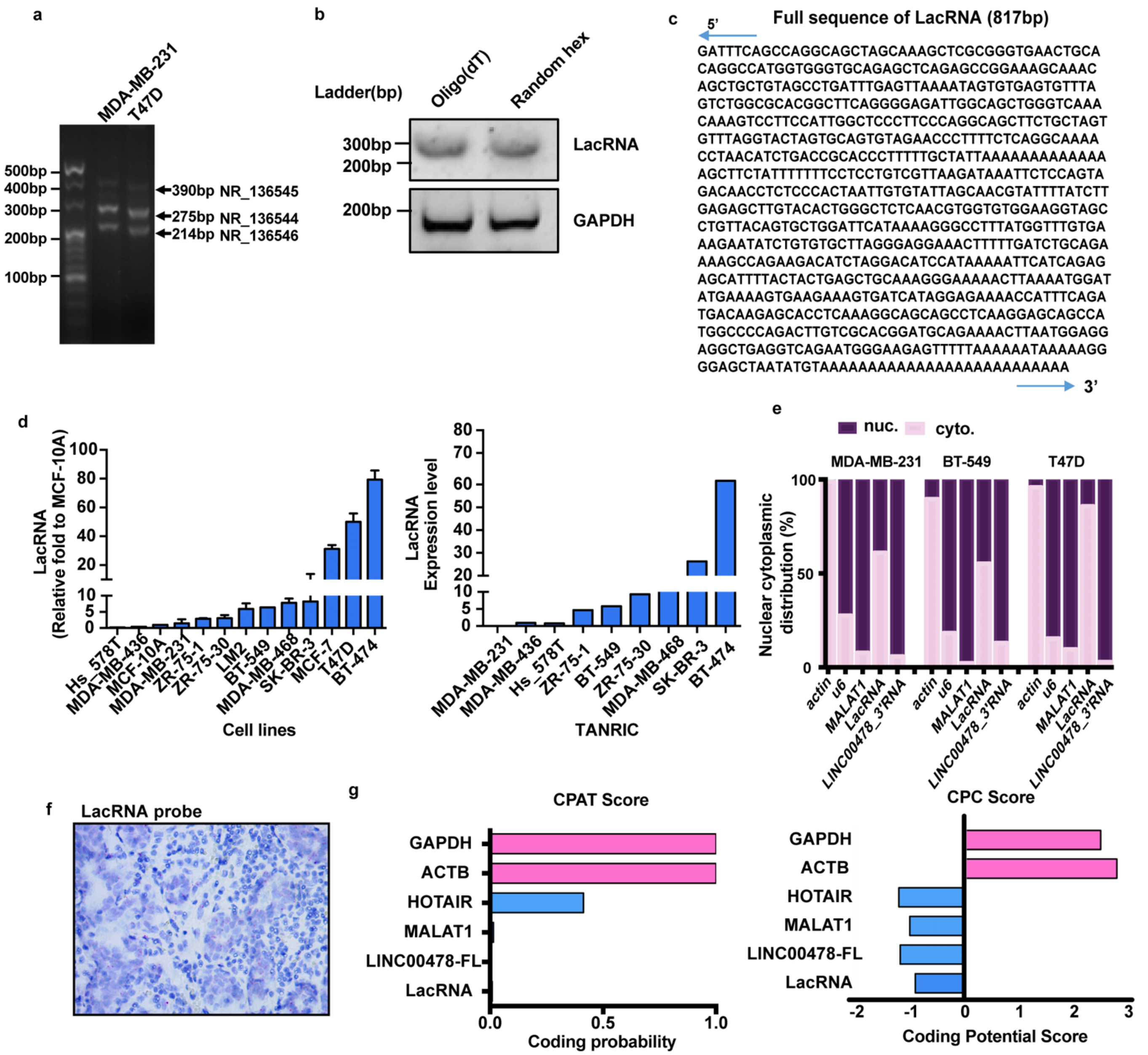

Figure S2. LacRNA is a small cytoplasm RNA derived from LINC00478 in breast cancer cells.

(a) The relative expression of isoforms NR_136544, NR_136545 and NR_136546 in MDA-MB-231 and T47D cells according to semi-quantitative PCR amplification of exon 7 and exon 11 junction. **(b)** RT-PCR experiment using oligo dT or Random primer to reverse-transcribe total cell RNA separately, to confirm the existence of the poly A tail. **(c)** Sequence of LacRNA cloned from MDA-MB-231 cell cDNA. The sequence identified by RACE assays. **(d)** The expression level of LacRNA in different breast cancer cell

lines measured by RT-qPCR and TANRIC database separately. **(e)** RT-qPCR of the nuclear and cytoplasmic fractionations of MDA-MB-231, BT549 and T47D cells. Compared with nuclear lncRNA MALAT, cytoplasmic internal reference actin and nuclear internal reference U6. The data shown represent three independent experiments. **(f)** Representative image of RNA scope assay to show the subcellular localization of LacRNA. red, LacRNA RNA. **(g)** The protein coding potential of LINC00478 full length and LacRNA, using Coding Potential Assessment Tool (CPAT, http://lilab.research.bcm.edu/cpat/) and Coding Potential Calculator (CPC, http://cpc.cbi.pku.edu.cn/). GAPDH and β-actin served as the positive controls of coding genes, and HOTAIR and MALAT1 served as the negative control of non-coding genes.

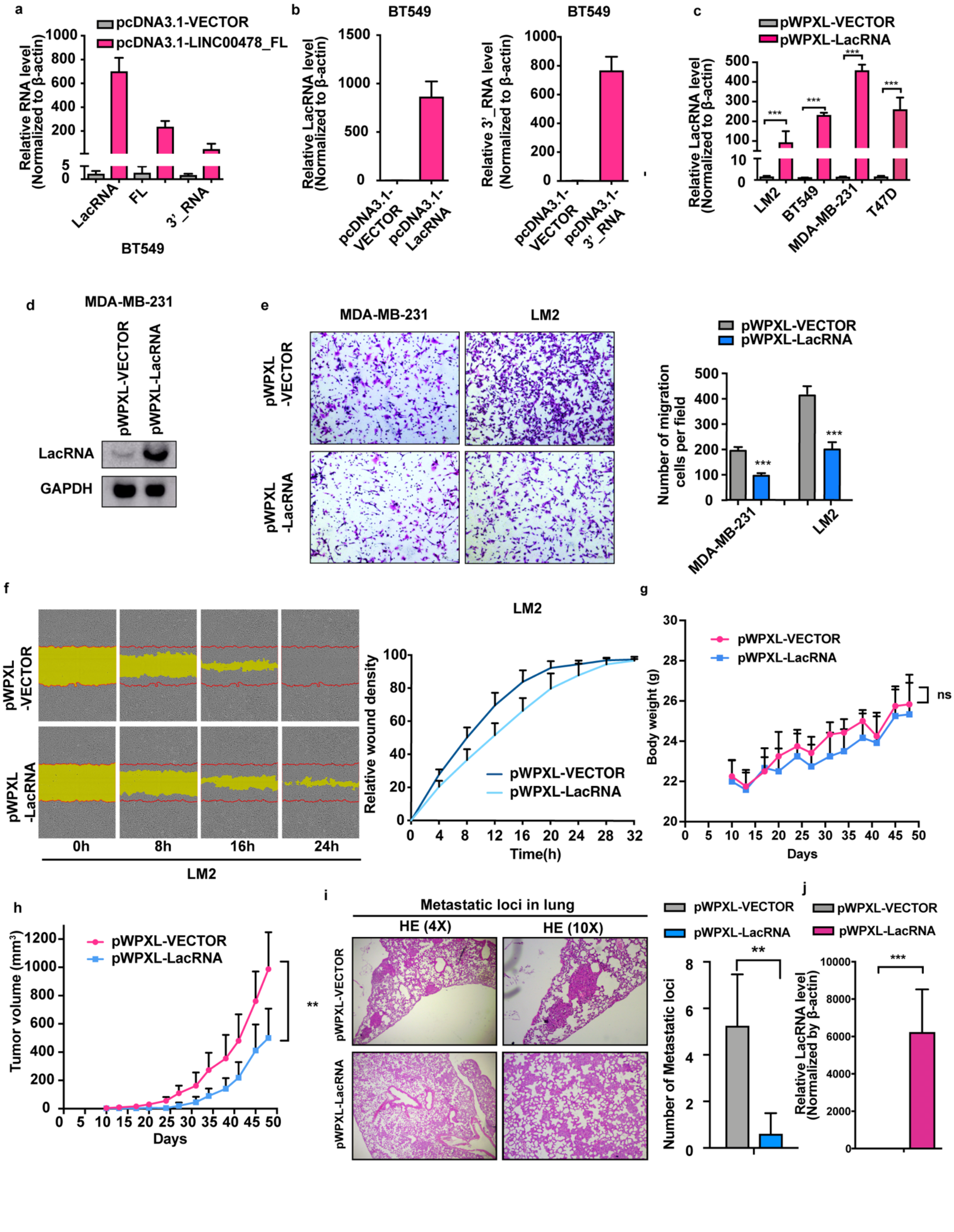

Figure S3. LacRNA inhibits breast cancer metastasis.

(a) The level of lacRNA, LINC00478 full length and LINC00478_3'RNA when LINC00478 full length was overexpressed by pcDNA3.1 in BT549 cells. **(b)** The overexpression of LacRNA and LINC00478_3'RNA by pcDNA3.1 in BT549 cells. **(c)** The overexpression of LacRNA by lentivirus in LM2, BT549, MDA-MB-231 and T47D cell lines. **(d)** Northern-blotting for LacRNA in the pWPXL overexpression MDA-MB-231 cells. **(e)** Transwell migration assay in the MDA-MB-231 and LM2 cells with LacRNA overexpressed by lentivirus. **(f)** Wound healing assay with representative images in stable pWPXL-LacRNA and pWPXL-vector control cells. **(g, h) Body weights and** tumor volumes were measured in the pWPXL-LacRNA and negative control groupsin the xenograft mouse models. **(i)** Representative data and statistical analysis of hematoxylin-eosin staining in the tumor foci of the lung samples obtained from NOD/SCID mice after injection with vector pWPXL-LacRNA and control pWPXL-vector LM2 cells, n=6. **(j)** Seven weeks after implantation of orthotopic transplanted tumors in mice, the orthotopic tumors were removed, and the expression level of LacRNA in orthotopic tumor was verified by qPCR. Values are expressed as the Mean ± SEM in a-c and e. n=3 independent experiments. *P* values were determined by unpaired Student's *t*-test. ns, not significant, **p<0.01, ***p<0.001.

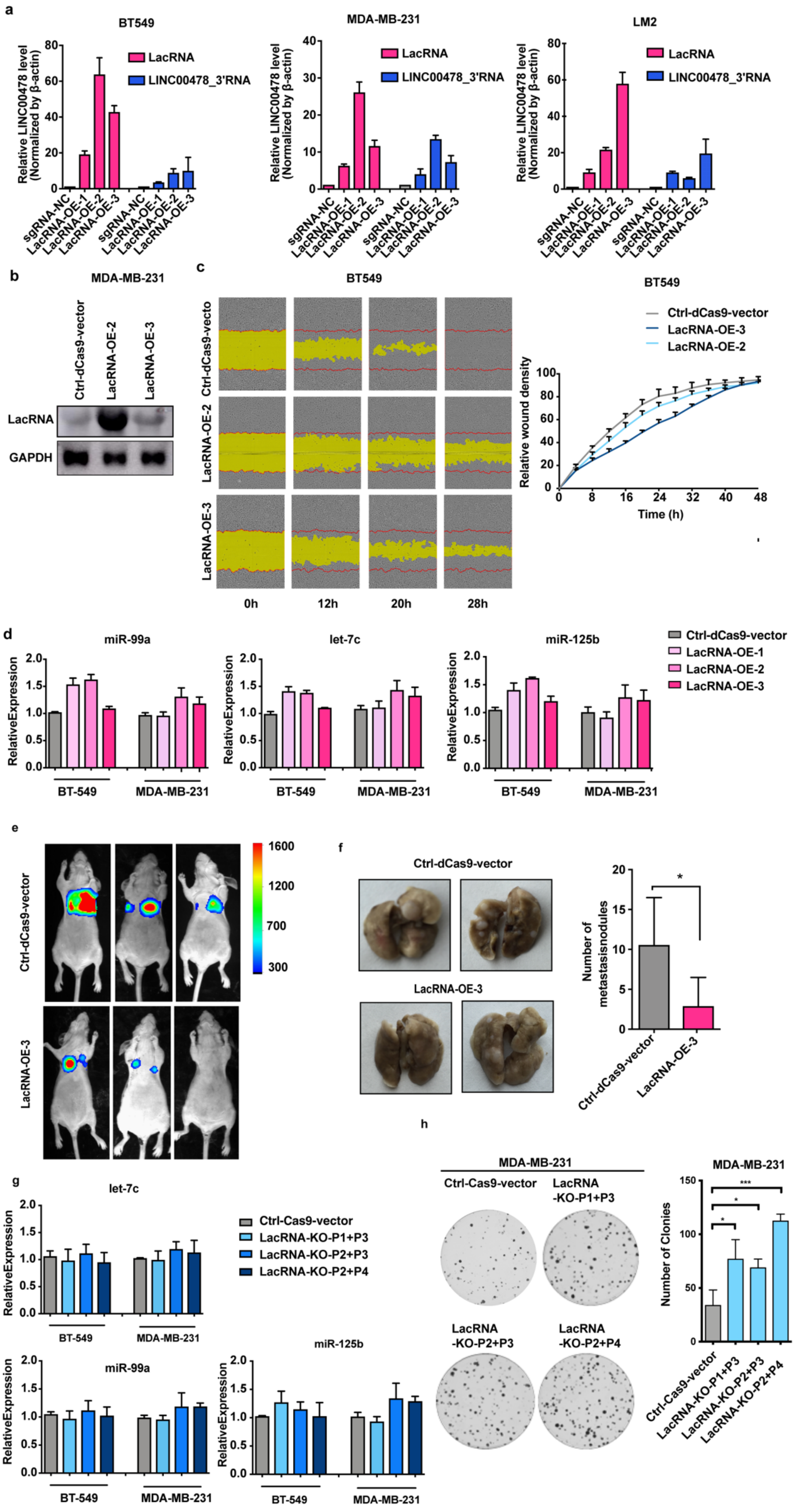
a
BT549
MDA-MB-231
LM2
Relative LINC00478 level (Normalized by β-actin)
LacRNA
LINC00478_3'RNA
sgRNA-NC
LacRNA-OE-1
LacRNA-OE-2
LacRNA-OE-3
b
MDA-MB-231
Ctrl-dCas9-vector
LacRNA-OE-2
LacRNA-OE-3
LacRNA
GAPDH
c
BT549
Ctrl-dCas9-vecto
LacRNA-OE-2
LacRNA-OE-3
0h
12h
20h
28h
BT549
Ctrl-dCas9-vector
LacRNA-OE-3
LacRNA-OE-2
Relative wound density
Time (h)
d
miR-99a
let-7c
miR-125b
RelativeExpression
BT-549
MDA-MB-231
Ctrl-dCas9-vector
LacRNA-OE-1
LacRNA-OE-2
LacRNA-OE-3
e
Ctrl-dCas9-vector
LacRNA-OE-3
1600
1200
700
300
f
Ctrl-dCas9-vector
LacRNA-OE-3
Number of metastasisnodules
Ctrl-dCas9-vector
LacRNA-OE-3
g
let-7c
miR-99a
miR-125b
RelativeExpression
BT-549
MDA-MB-231
Ctrl-Cas9-vector
LacRNA-KO-P1+P3
LacRNA-KO-P2+P3
LacRNA-KO-P2+P4
h
MDA-MB-231
Ctrl-Cas9-vector
LacRNA -KO-P1+P3
LacRNA -KO-P2+P3
LacRNA -KO-P2+P4
MDA-MB-231
Number of Clonies
Ctrl-Cas9-vector
LacRNA-KO-P1+P3
LacRNA-KO-P2+P3
LacRNA-KO-P2+P4

Figure S4. LacRNA inhibits breast cancer metastasis.

(a) The activation of LacRNA and 3′RNA by CRISPR-dCas9 technology activation in the BT549, MDA-MB-231 and LM2 cell lines, Mean ± SEM; n=3 independent experiments. **(b)** Northern-blotting for LacRNA in the CRISPR-OE-2 or CRISPR-OE-3 cells, derived from MDA-MB-231 cells. **(c)** Representative images of wound healing assay in the BT-549 cells with LacRNA activation by the CRISPR/dead-Cas9 technology. **(d)** The expression of the microRNAs (let-7c, miR125b and miR99a) existed in the introns of LacRNA by qRT-PCR in LacRNA-endogenous- activated BT549 and MDA-MB-231 cells. **(e)** Representative images from BLI of the lungs of athymic BALB/c nude mice 5 weeks after the indicated cells were injected into the caudal vein, n=6. **(f)** Representative data and statistical analysis of lung metastasis in the control groups and LacRNA-activated groups. **(g)** The expression of the microRNAs (let-7c, miR125b and miR99a) existed in the introns of LacRNA by qRT-PCR in LacRNA-endogenous- knockout BT549 and MDA-MB-231 cells. **(h)** Representative images of colony formation assays in LacRNA-endogenous-knockout MDA-MB-231 cells. Values are expressed as the Mean ± SEM; n=3 independent experiments. *P* values were determined by one way analysis of variance (ANOVA). *$p<0.05$, ***$p<0.001$.

a

LacRNA pulldown		Peptide number
**LacRNA sense**		
Prohibitin	PHB	18
Elongation factor 1-alpha	EEF1A1	6
ADP/ATP translocase 2	SLC25A5 ANT2	5
Heterogeneous nuclear ribonucleoprotein A1-like	HNRNPA1L2	3
**LacRNA anti-sense**		
Hornerin	HRNR	2
Heterogeneous nuclear ribonucleoprotein A1	HNRPA1	3
Tubulin	TUBA	2
**Beats only**		
Tubulin	TUBA	2
Serum albumin	ALB	3

b

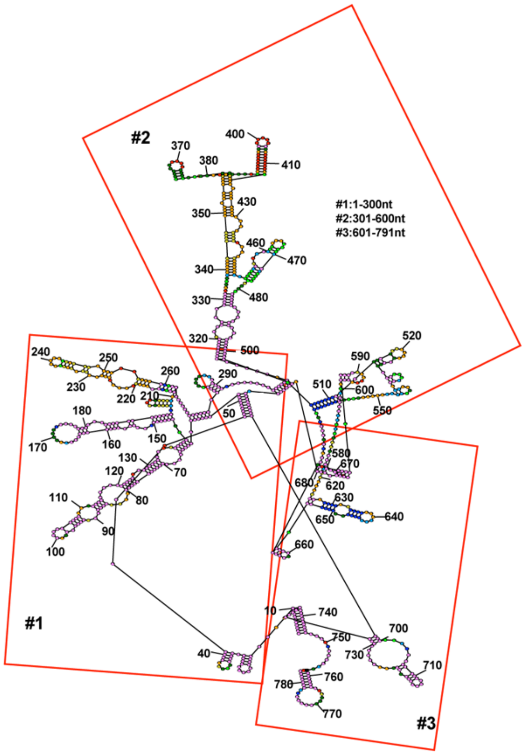

c

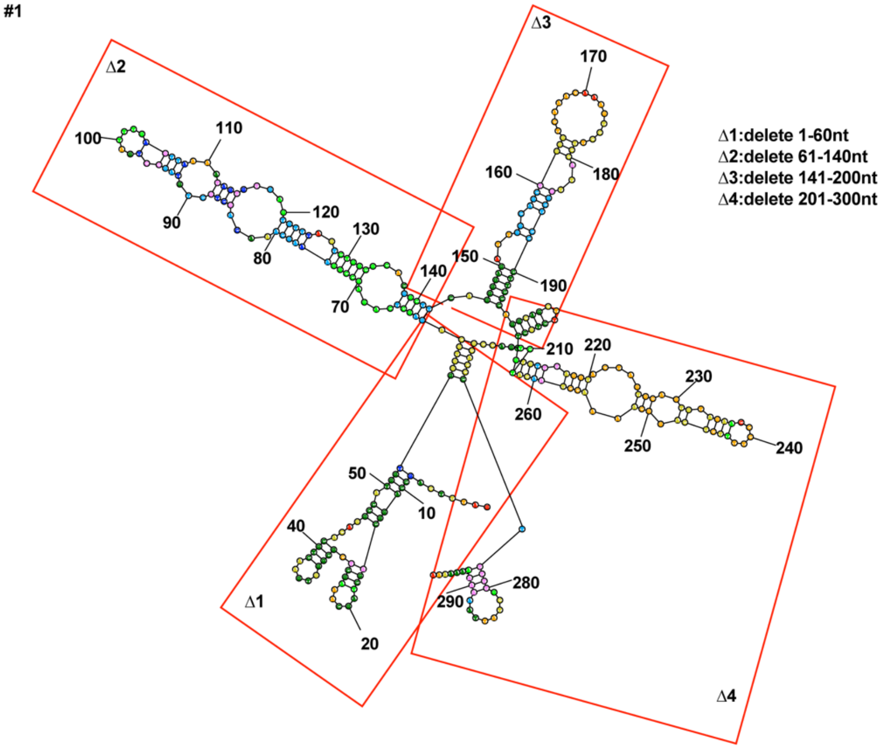

d

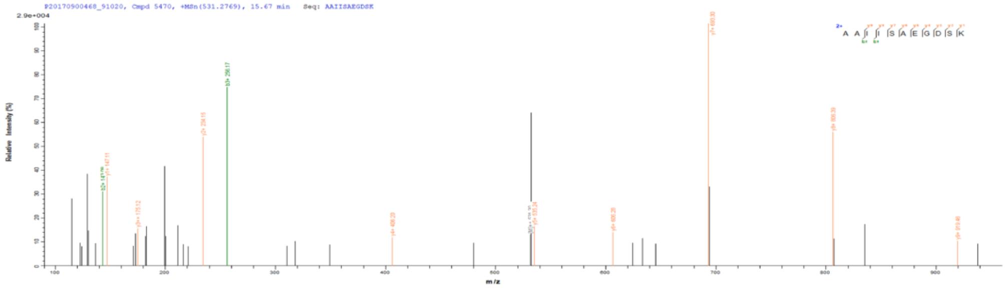

Figure S5. Streptavidin pulldown assay and graphic illustration of predicted LacRNA secondary structure.

(a) A list of the top LacRNA-associated proteins identified by RNA pulldown and MS analysis in MDA-MB-231 cells. **(b)** Schematic view of the predicted secondary structure of LacRNA and truncation-mapping of biotinylated LacRNA motifs as indicated. The red boxes represent the three truncation fragments of LacRNA, with the corresponding number labeled. **(c)** Schematic view of the predicted secondary structure of LacRNA 1-300nt motif and deletion-mapping of biotinylated LacRNA motifs as indicated. The red boxes represent the deleted fragments of LacRNA, with the corresponding number labeled. **(d)** The mass spectrometry identification of 30- kDa LacRNA-binding proteins.

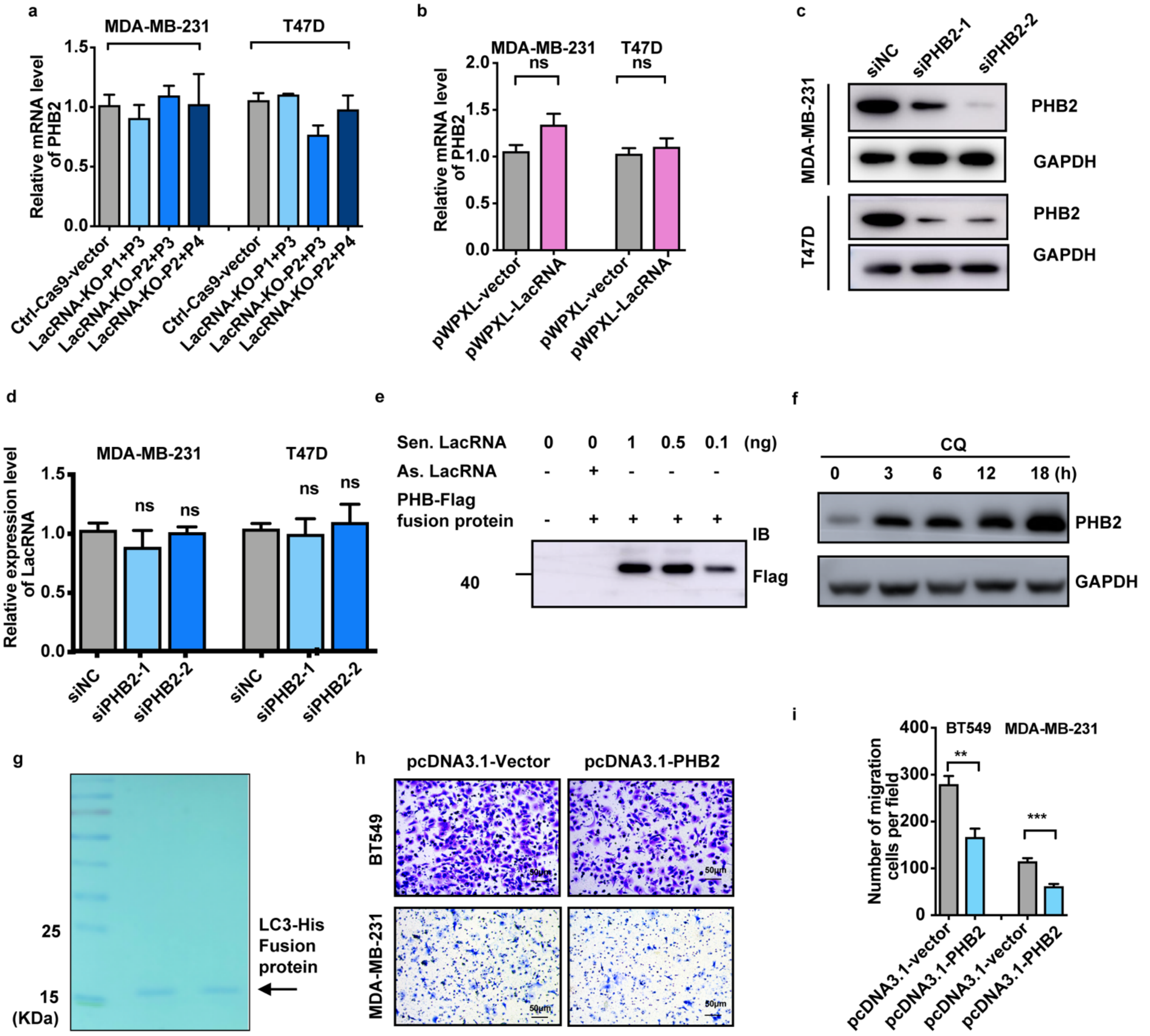

Figure S6. LacRNA physically interacts with PHB2 in breast cancer cells.

(a, b) The mRNA level of PHB2 was quantified by RT-qPCR in MDA-MB-231 and T47D cells with LacRNA knockout **(a)** or overexpression **(b)**, Mean ± SEM; n=3 independent experiments. **(c)** Immunoblotting of the efficiencies of two independent siRNAs for PHB2 in MDA-MB-231 and T47D cells. **(d)** The expression level of LacRNA was quantified by RT-qPCR in MDA-MB-231 and T47D cells with PHB2 knockdown. **(e)** Immunoblotting of PHB2 in pulldown samples by different dencity biotinylated-LacRNA. **(f)** Immunoblotting for PHB2 levels in the breast cancer cells treated with autophagy inhibitors chloroquine (CQ) for indicated times. **(g)** Exogenous synthetic LC3-His protein. **(h)**

Representative images of transwell migration assays for BT-549 and MDA-MB-231 cells overexpressing PHB2. **(i)** Quantitative images of transwell migration assays of BT-549 and MDA-MB-231 cells with PHB2 overexpressed.

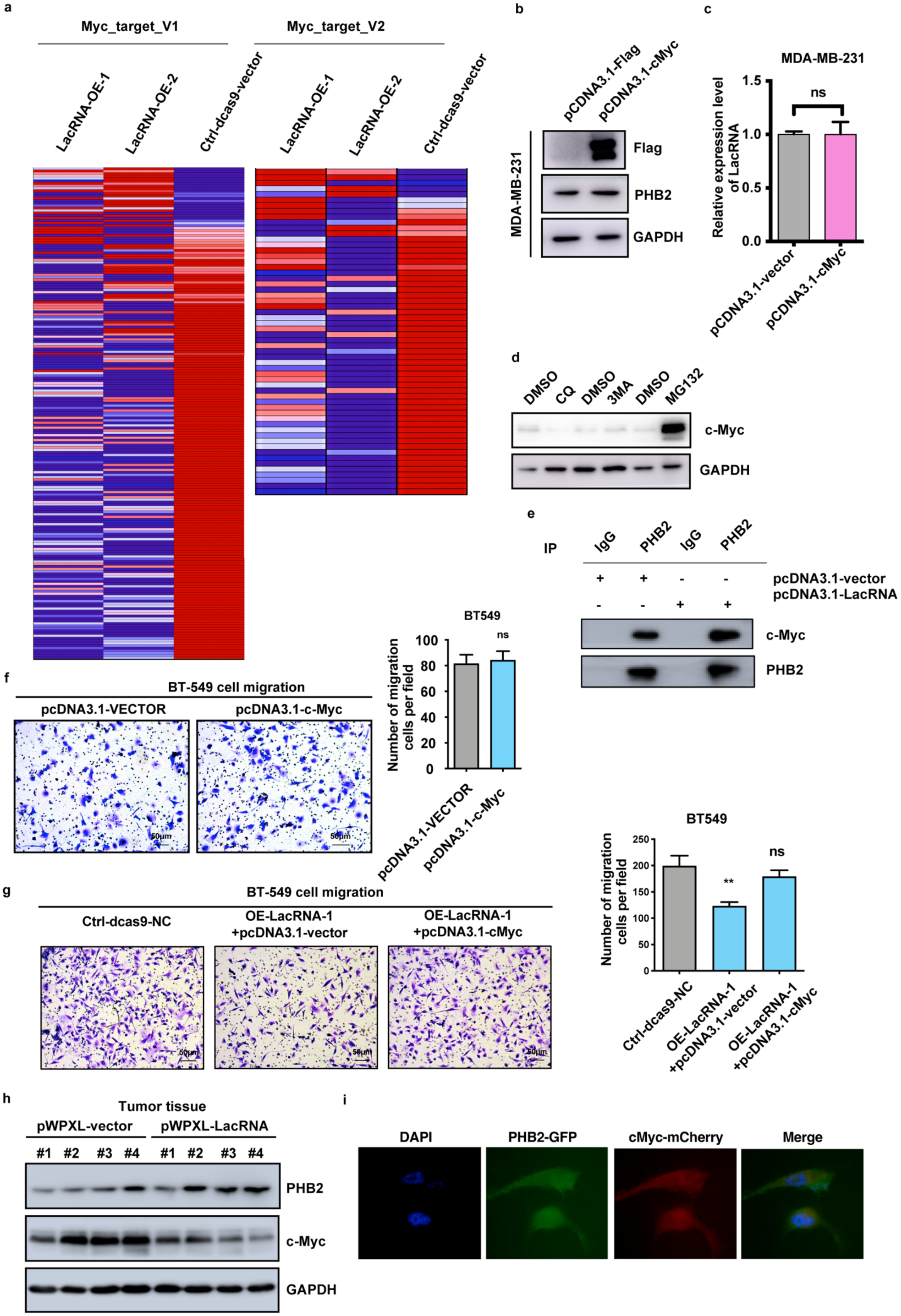

a
Myc_target_V1
Myc_target_V2
LacRNA-OE-1
LacRNA-OE-2
Ctrl-dcas9-vector
b
pCDNA3.1-Flag
pCDNA3.1-cMyc
MDA-MB-231
Flag
PHB2
GAPDH
c
MDA-MB-231
Relative expression level of LacRNA
ns
pCDNA3.1-vector
pCDNA3.1-cMyc
d
DMSO
CQ
DMSO
3MA
DMSO
MG132
c-Myc
GAPDH
e
IP
IgG
PHB2
IgG
PHB2
pcDNA3.1-vector
pcDNA3.1-LacRNA
c-Myc
PHB2
f
BT-549 cell migration
pcDNA3.1-VECTOR
pcDNA3.1-c-Myc
BT549
Number of migration cells per field
ns
g
BT-549 cell migration
Ctrl-dcas9-NC
OE-LacRNA-1 +pcDNA3.1-vector
OE-LacRNA-1 +pcDNA3.1-cMyc
BT549
Number of migration cells per field
**
ns
h
Tumor tissue
pWPXL-vector
pWPXL-LacRNA
#1 #2 #3 #4 #1 #2 #3 #4
PHB2
c-Myc
GAPDH
i
DAPI
PHB2-GFP
cMyc-mCherry
Merge

Figure S7. The LacRNA-PHB2 complex is a negative regulator of MYC signaling.

(**a**) Heat map of RNA-sequencing data after LacRNA activation showing different expression of MYC targets genes in GSEA MYC target gene set v1 and v2. **(b)** Immunoblotting for the protein levels of PHB2 and c-Myc after c-Myc overexpressed. GAPDH served as the internal control. **(c)** The expression level of LacRNA was quantified by RT-qPCR in MDA-MB-231 cells with c-Myc overexpressed. **(d)** Immunoblotting for the protein levels of c-Myc in MDA-MB-231 cells with the proteasome inhibitor MG132 and autophagy inhibitors chloroquine (CQ) and 3-methyladenine (3MA) treated. **(e)** Co-immunoprecipitation of c-Myc and PHB2 with or without the LacRNA overexpressed. **(f)** Representative images and quantitative of transwell migration assay in the BT-549 cells with c-Myc overexpressed, Mean ± SEM; n=3 independent experiments. ns, not significant. **(g)** Representative images and quantitative of the transwell migration rescue assays in LacRNA activated BT549 cells with additional c-Myc overexpressed, Mean ± SEM; n=3 independent experiments. ns, not significant, $^{**}p<0.01$. **(h)** Immunoblotting of the expression of PHB2 and c-Myc in orthotopic transplanted tumors in mice. **(i)** Confocal microscopy imaging of co-immunostaining of PHB2-GFP (green), c-Myc-mCherry (red) and merged with DAPI (blue) in MDA-MB-231 cell overexpressing PHB2-GFP and c-Myc-mCherry.

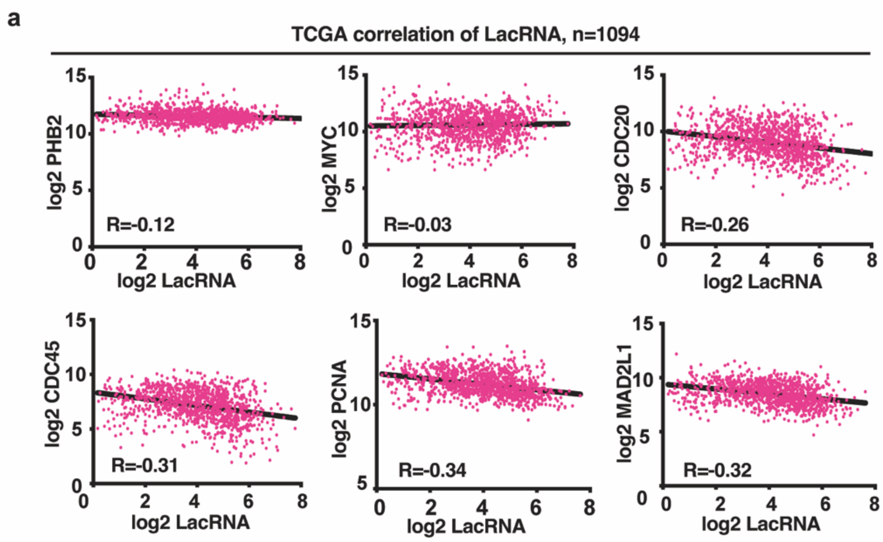

Figure S8. The correlation of LacRNA and PHB2, c-Myc and its downstream pathways in RNA-seq data for 1,094 breast cancer patients from the TCGA database. linear regression analysis.

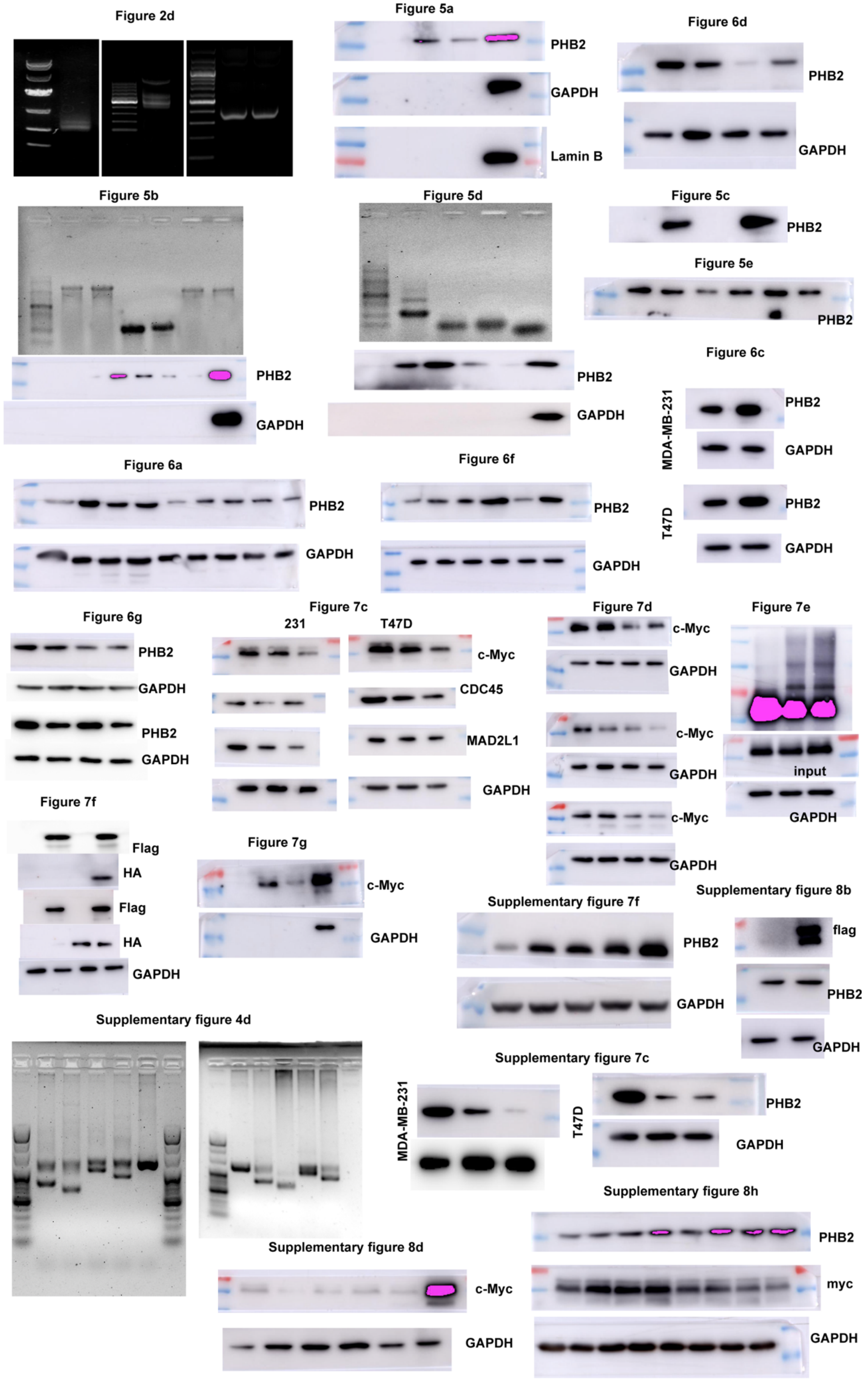

Figure S9. The uncropped original scans of blots.

Additional Tables

Table S1: Long non-coding RNAs differentially expressed in primary BC and positive lymph node.

LincRNA ID	LncRNA	Description	Chromosome Cytoband	Start~ End	Strand	log2 Fold change	*P*_value	style
ENSG00000230838	LINC01614	long intergenic non-protein coding RNA 1614	chr2 q35	216582766~216584147	+	1.9468742	0.0024375	down
ENSG00000249835	VCAN-AS1	VCAN antisense RNA 1 (non-protein coding)	chr5 q14.3	82837296~82877139	-	1.634395	0.0007848	down
ENSG00000231106	LINC01436	long intergenic non-protein coding RNA 1436	chr21 q22.12	37377636~37379899	+	1.363169	0.0148049	down
ENSG00000215386	LINC00478	long intergenic non-protein coding RNA 478	chr21 q21.1	17442842~17982094	+	0.9276386	0.044495	down
ENSG00000214548	MEG3	maternally expressed 3 (non-protein coding)	chr14 q32	101245747~101327368	+	0.9266954	0.0072912	down
ENSG00000261742	LINC00922	long intergenic non-protein coding RNA 922	chr16 q21	65318402~65610203	-	0.6800536	0.0087496	down
ENSG00000247982	LINC00926	long intergenic non-protein coding RNA 926	chr15 q21.3	57592563~57599967	+	-0.7379656	0.043048	up
ENST00000427859	CHRM3-AS2	CHRM3 antisense RNA 2 (non-protein coding)	chr1 q43	239867192~239882419	-	-0.9022458	0.0412862	up
ENST00000499452	LINC02273	long intergenic non-protein coding RNA 2273	chr4 q31.3	153021906~153025872	+	-1.0046252	0.0361784	up
ENSG00000256039	LINC02446	long intergenic non-protein coding RNA 2446	chr12 p13.2	10705962~10710648	+	-1.1876758	0.0428353	up

Table S2: Sequences for the primers, PCR, sgRNAs and shRNA.

Primer name or Sequences of PCR, sgRNAs and shRNA primers	Sequence (5’→3’)	Application
groupA-F (AF)	AAGGCCCAACCAGTTCTTCA TC	qPCR analysis
groupA-R (AR)	TTTCTGCA TCCGTGCGACAA	qPCR analysis
groupB-F (BF)	ACTGAAATGGAACAGGGGCA	qPCR analysis
groupB-R (BR)	AGTTTTCTGCATCCGTGCGA	qPCR analysis
groupC-F (CF)	TACACTGGGCTCTCAACGTG	qPCR analysis
groupC-R (CR)	AAGTTTTCTGCATCCGTGCG	qPCR analysis
NR_136544-45-F	TCTGTGTGCTTAGGGAGGAAAC	qPCR analysis
NR_136544-45-R	CTGAAATGGTTTTCTCCTATGATCACT	qPCR analysis
NR_136545-F	TCTGTGTGCTTAGGGAGGAAAC	qPCR analysis
NR_136545-R	TGTTGACTCATGGCCTAGGGAAA	qPCR analysis
semi-quantitative rt-PCR TSS5-F	TGGGCTCTCAACGTGGTGTGGAAGGTAG	semi-quantitative rt-PCR analysis
semi-quantitative rt-PCR TSS5-R	GGCTGCTGCCTTTGAGGTGCTCTTGT	semi-quantitative rt-PCR analysis
LINC00478-5’ RACE	GGCTGCTGCCTTTGAGGTGCTCTTGT	5'RACE
LINC00478-3’ RACE-1	GAGAGCTTGTACACTGGGCTCTCAACGTGG	3'RACE
LINC00478-3’ RACE-2	GTGGAACACCAGCCAAGTCCTGGGC	3'RACE
pWPXL-LINC00478-F-BamHI	TTAAACTACGGGATCCGATTTCAGCCAGGCAGCTAG	PCR for vector construction
pWPXL-LINC00478-R-MluI	TAGCGCTAGGACGCGTTTTTCTGTTATTTCTTCTTTATTTCTA	PCR for vector construction
pWPXL-LacRNA-R-MluI-1	TAGCGCTAGGACGCGTCTCTTCCCATTCTGACCTCAGCC	PCR for vector construction
pWPXL-LacRNA-R-MluI-2	TAGCGCTAGGACGCGTACATATTAGCTCCCCTTTTTATTTTTTAAAAACTCTTCC CA TTCTG	PCR for vector construction
pWPXL-LINC00478_3'RNA-F-BamHI	TTAAACTACGGGATCCGCTAATATGTGAGGAACTGATTGCTG	PCR for vector construction
pcDNA3.1-LINC00478-F-NotI	AAGGAAAAAAGCGGCCGCGATTTCAGCCAGGCAGCTAG	PCR for vector construction
pcDNA3.1-LINC00478-R-BamHI	TTAAACTACGGGATCCTTTTCTGTTATTTCTTCTTTATTTCTA	PCR for vector construction
pcDNA3.1- LacRNA-R-BamHI	TTAAACTACGGGATCCCATATTAGCTCCCCTTTTTATTTTTTAAAAACTC	PCR for vector construction
pcDNA3.1- LINC00478_3'RNA -F- NotI	AAGGAAAAAAGCGGCCGCGAGCTAATATGTGAGGAACTG	PCR for vector construction
LacRNA-Norhtern Probe-F	AACATCTGACCGCACCCTTT	Northern Blotting
LacRNA-Norhtern Probe-R	GATCACTAATACGACTCACTATAGGGCTTTGCAGCTCAGTAGTAAAATGCTC	Northern Blotting
LINC00478_3'RNA-Northern-F	CCAACCATGCCTGCTAGTGA	Northern Blotting
LINC00478_3'RNA-Northern-R	GATCACTAATACGACTCACTATAGGGGGCTTCACATAGGTTGGGCT	Northern Blotting
LacRNA-sgRNA-1	AACAGTCCAGATTTACCATG	CRISPR/Cas9 and CRISPR/dead-Cas9
LacRNA-sgRNA-2	TAGCTCAACAAATCCCAACC	CRISPR/Cas9 and CRISPR/dead-Cas9

LacRNA-sgRNA-3	TCTGATTTTTGCAAAGCTCG	CRISPR/Cas9 and CRISPR/dead-Cas9
LacRNA-sgRNA-4	TGAACTGCACAGGCCATGGT	CRISPR/Cas9 and CRISPR/dead-Cas9
LINC00478-FL-sense-Pulldown-F	TAATACGACTCACTATAGGGAGAGATTTCAGCCAGGCAGCTAG	*In vitro* transcription
LINC00478-FL-sense-Pulldown-R	TTTTCTGTTATTTCTTCTTTATTTCTA	*In vitro* transcription
LINC00478-FL-antisense-Pulldown-F	TAATACGACTCACTATAGGGAGATTTTCTGTTATTTCTTCTTTATTTCTATTTTTATTTCT	*In vitro* transcription
LINC00478-FL-antisense-Pulldown-R	GGATCCGATTTCAGCCAGGCAGCTAG	*In vitro* transcription
LacRNA-sense-Pulldown-R	ACATATTAGCTCCCCTTTTTAT	*In vitro* transcription
LacRNA-antisense-Pulldown-F	AATACGACTCACTATAGGGAGAACATATTAGCTCCCCTTTTTAT	*In vitro* transcription
LacRNA-#1-Pulldown-R	CTTTTTTTTTTTTTTAATAGCAAAAAGGGTGCG	*In vitro* transcription
LacRNA-#2-Pulldown-F	TAATACGACTCACTATAGGGAGAGCTTCTATTTTTTTCCTCCTG	*In vitro* transcription
LacRNA-#2-Pulldown-R	TTAAGTTTTCCCTTTGCAGCTCAGTAG	*In vitro* transcription
LacRNA-#3-Pulldown-F	TAATACGACTCACTATAGGGAGAATGGATATGAAAAGTGAAGAAAG	*In vitro* transcription
LacRNA-Δ1-Pulldown-R	GGTCTCCCTATAGTGAGTCGTATTAATTTCG	*In vitro* transcription
LacRNA-Δ1-Pulldown-F	GAGCTCAGAGCCGGAAAGCAAACAG	*In vitro* transcription
LacRNA-Δ2-Pulldown-R	TGCACCCACCATGGCCTGTGCAGTTC	*In vitro* transcription
LacRNA-Δ2-Pulldown-F	TTCAGGGGAGATTGGCAGCTGGGTC	*In vitro* transcription
LacRNA-Δ3-Pulldown-R	GCCGTGCGCCAGACTAAACACTCAC	*In vitro* transcription
LacRNA-Δ3-Pulldown-F	GCTTCTGCTAGTGTTTAGGTACTAGTGCAGTGT	*In vitro* transcription
LacRNA-Δ4-Pulldown-R	TGCCTGGGAAGGGAGCCAATGGAAG	*In vitro* transcription
LacRNA-Δ4-Pulldown-F	GCTTCTATTTTTTTCCTCCTGTCGTTAAGA	*In vitro* transcription
PHB2-FL-F-HindIII	CCCAAGCTTATGGCCCAGAACTTGAAGGACT	PCR for vector construction
PHB2-FL-R-KpnI	CGGGGTACCTTTCTTACCCTTGATGAGGCTGTCAC	PCR for vector construction
PHB2-N201-R-KpnI	CGGGGTACCTTGTTTGGCTTCTACAGCAGCTG	PCR for vector construction
PHB2-ΔN39-F- HindIII	CCCAAGCTTATGTCTGTGTTCACCGTGGAAGGCG	PCR for vector construction
PHB2-N39-R-KpnI	CGGGGTACCGTAGGCCACGGCGCCGGCCCCCAGCAACA	PCR for vector construction
PHB2-ΔN188-F- HindIII	CCCAAGCTTATGAGCTTTAGCCGAGAGTACACAG	PCR for vector construction
hsa-miR-125b-5p-F	TCCCTGAGACCCTAACTTGTGA	qPCR analysis
hsa-miR-99a-5p-F	AACCCGTAGATCCGATCTTGTG	qPCR analysis
hsa-let7c-F	TGAGGTAGTAGGTTGTATGGTT	qPCR analysis
c-Myc-CDS-F-HindIII	CCCAAGCTTATGCCCCTCAACGTTAGCTTCAC	PCR for vector construction
c-Myc-CDS-R-KpnI	CGGGGTACCCGCACAAGAGTTCCGTAGCTGTT	PCR for vector construction
c-Myc-CDS-F-EcoRI	CCGGAATTCATGCCCCTCAACGTTAGCTTCAC	PCR for vector construction
c-Myc-RT-F	AATGAAAAGGCCCCCAAGGTAG	qPCR analysis
c-Myc-RT-R	GTCGTTTCCGCAACAAGTCCT	qPCR analysis
CCNA2-RT-F	TGATGTTGGGCAACTCTGCG	qPCR analysis

CCNA2-RT-R	GTGCAACCCGTCTCGTCT	qPCR analysis
CDC20-RT-F	CGCTATATCCCCCATCGCAG	qPCR analysis
CDC20-RT-R	CTGATAACCCTCTGGCGCAT	qPCR analysis
CDC45-RT-F	GTGATTTGGCGGGAGTCTTG	qPCR analysis
CDC45-RT-R	CGAAGAGAAGGACCCTCTGG	qPCR analysis
MAD2L1-RT-F	CGTGGCCGAGTTCTTCTCATT	qPCR analysis
MAD2L1-RT-R	TACAAGCAAGGTGAGTCCGT	qPCR analysis
APEX1-RT-F	TGAAGCCTTTCGCAAGTTCCT	qPCR analysis
APEX1-RT-R	ATGTGCCACATTGAGGTCTCC	qPCR analysis
POLB2-RT-F	CCACCCGCCTCATCCAAAT	qPCR analysis
POLB2-RT-R	CCAAGACCAGCTCGTCATCTG	qPCR analysis
PCNA-RT-F	ACACTAAGGGCCGAAGATAACG	qPCR analysis
PCNA-RT-R	ACAGCATCTCCAATATGGCTGA	qPCR analysis
CDKN1A-RT-F	GTCACTGTCTTGTACCCTTGTG	qPCR analysis
CDKN1A-RT-R	GGGCTTCCTCTTGGAGAAGAT	qPCR analysis
RAN-RT-F	ATGTAGCCACCTTGGGTGTTG	qPCR analysis
RAN-RT-R	GGGGATGTTTTCACACACTCG	qPCR analysis
CCT2-RT-F	GAACGCCTAGCTCTTGTCACA	qPCR analysis
CCT2-RT-R	GCACCACGCAAAACAATGGTA	qPCR analysis
VBP1-RT-F	AGTCCACCAACTCAATGGAGA	qPCR analysis
VBP1-RT-R	CAAGCATTACATTAGCCCCCAA	qPCR analysis
PSMA7-RT-F	ACCGCCGATGCAAGGATAG	qPCR analysis
PSMA7-RT-R	CTGCGTATAACGCTGCTTCAG	qPCR analysis
RFC4-RT-F	CCGCTGACCAAGGATCGAG	qPCR analysis
RFC4-RT-R	AGGGAACGGGTTTGGCTTTC	qPCR analysis
PHB2-siRNA-1	GCGAGTGTTGTCTCGACCCAA	PHB2 knockdown
PHB2-siRNA-2	GCTGAGCTTTAGCCGAGAGTA	PHB2 knockdown
c-Myc-siRNA-1	CCTGAGACAGATCAGCAACAA	c-Myc knockdown
c-Myc-siRNA-2	CAGTTGAAACACAAACTTGAA	c-Myc knockdown

Table S3: Antibodies for Immunoblotting, RIP and IP.

Protein Name	Company	Catalog Number	Dilutions in IB	Dilutions in RIP/IP
PHB2	Proteintech	12295-1-AP	1:1000	5μg
HRP-conjugated GAPDH	Proteintech	HRP-60004	1:10000	N/A
Lamin B1	Proteintech	12987-1-AP	1:1000	5μg
c-Myc	abcam	ab32072	1:1000	5μg
CDC45	Proteintech	15678-1-AP	1:1000	N/A
MAD2L1	Proteintech	10337-1-AP	1:1000	N/A
FLAG	sigma	F7425	1:1000	5μg
HA	sigma	H3663	1:1000	5μg
His	Proteintech	66005-1-Ig	1:5000	5μg
Goat Anti-Rabbit IgG, Fc fragment specific	Jackson			N/A
Goat Anti-Rabbit IgG, F(ab')2 fragment specific	Jackson			N/A
Goat Anti-Mouse IgG, F(ab')2 fragment specific	Jackson			N/A
Goat Anti- Mouse IgG, Fc fragment specific	Jackson			N/A

Abbreviations: IB: Immunoblotting; RIP: RNA immunoprecipitation; IP: immunoprecipitation.

Table S4: RNA-seq analysis of the gene expression profile affected by LacRNA activated with two sgRNA.

Gene ID	Signal Intensity (FPKM)			FC_(OE1/Ctrl)	FC_(OE2/Ctrl)
	Ctrl-dcas9-vector	LacRNA-OE-1	LacRNA-OE-2		
BBX	1.31210123	2.609767006	2.84216971	1.988998216	2.166120757
COL13A1	4.345154438	7.50817572	10.67766634	1.7279422	2.457373264
SPDEF	0.133530069	0.071337505	0.035642441	0.534242999	0.266924455
BIK	0.35757475	0.071636928	0.071584084	0.200341125	0.200193341
FBXO48	0.125328701	0.426844378	0.125449857	3.405799119	1.000966706
HTR1D	0.240401225	0.457541392	0.794090955	1.903240684	3.303190131
BOC	0.933763463	2.435593954	2.096584435	2.608362878	2.24530571
C1S	0.727361231	1.57863731	2.572494128	2.170362184	3.536749029
C1R	13.74067487	33.69966596	38.07607368	2.452548094	2.771048296
BSN	0.069309619	0.132446946	0.153695898	1.910946112	2.217526242
CRISPLD1	0.64942067	1.89244607	1.780738835	2.914052722	2.742042129
IGF1R	2.449922934	5.148423374	5.601003671	2.101463398	2.286195861
ENPP5	0.132251774	0.264954691	0.405964175	2.003411247	3.069631233
CA4	1.528734137	3.730904945	3.283000236	2.440519155	2.14752857
CA6	0.249099954	0.149714895	0.049868152	0.601023374	0.200193341
CA9	3.41804508	1.286546961	0.870888919	0.376398477	0.254791525
CCK	1.20150159	0.275097348	0.824683261	0.228961285	0.68637717
TBX15	0.1894095	0.624009314	0.589843656	3.294498495	3.114118642
BCAM	2.358862576	4.695924835	5.905342672	1.990758123	2.503470415
CFH	0.309639632	1.191044202	1.024864838	3.846549594	3.309863242
CFI	0.076371299	0.183603744	0.244624409	2.404093496	3.20309346
ZNF559-ZNF177	0.14988069	0.360327192	0.288049115	2.404093496	1.921856076
STON1	0.226856517	0.327230567	0.593424812	1.442456098	2.615859659
EDARADD	0.176808407	0.047229327	0.117986219	0.2671215	0.667311138
PLIN4	0.013074307	0.041909142	0.041878227	3.205457995	3.20309346
DIO2	0.040109315	0.085712482	0.096355412	2.136971996	2.402320095
DIO3	2.148042703	9.061526052	16.38958809	4.218503682	7.630010365
COL14A1	0.088476889	0.28360895	0.451668341	3.205457995	5.104930202
CPZ	0.06708046	0.107511798	0.214864983	1.602728997	3.20309346
SAPCD1	0.093004568	0.149061118	0.372377904	1.602728997	4.003866825
MAB21L3	0.026383457	0.084571065	0.04225434	3.205457995	1.60154673
MAB21L1	0.029305877	0.046969379	0.093869463	1.602728997	3.20309346
IL1R2	0.290559973	0.139706668	0.093069075	0.480818699	0.320309346
TCEAL8	3.022494984	0.90125402	1.688604753	0.298182139	0.558679092
LOC101927989	0.119316785	0.286848706	0.286637109	2.404093496	2.402320095
SVOP	0.294443033	0.098315081	0.098242558	0.333901874	0.333655569
C3orf58	0.273866953	0.736782921	0.657916084	2.690295103	2.402320095
LOC101927680	0.033382517	0.053503129	0.133659154	1.602728997	4.003866825
HLA-DMB	0.243928945	0.244344996	0.732494259	1.001705623	3.002900119
TTLL9	0.385009871	0.308533242	0.056055573	0.801364499	0.145595157
HLA-DOB	0.186280286	0.447835224	0.298336583	2.404093496	1.60154673
MB	0.291255331	0.816905887	1.399377067	2.804775745	4.80464019
DSP	0.639160272	1.66289703	1.269039403	2.601690222	1.985479165

SH2D3C	0.027010838	0.303037377	0.194666039	11.21910298	7.206960285
COL15A1	0.942739036	6.232690157	7.222070966	6.611257114	7.660731859
LOC102724752	0.07433873	0.297862094	0.357170848	4.006822493	4.80464019
LINGO1	0.02449459	0.03925819	0.058843846	1.602728997	2.402320095
LINGO3	0.159985322	0.064103279	0.064055993	0.400682249	0.400386683
LOC102724705	0.139202915	0.223104549	0.445879948	1.602728997	3.20309346
EFS	0.109220749	0.240695485	0.2623832	2.203752371	2.402320095
EGF	0.01521289	0.02438214	0.060910386	1.602728997	4.003866825
SWT1	0.132663666	0.194905246	0.371817356	1.469168248	2.802706778
GPR161	0.679478448	1.683030618	1.904378851	2.476944814	2.802706778
SLC16A11	0.047250241	0.113593997	0.113510203	2.404093496	2.402320095
SLC16A14	0.135594654	0.217321484	0.341253275	1.602728997	2.51671629
EN2	0.125467134	0.040217963	0.080376591	0.320545799	0.640618692
GPR155	0.067666548	0.099413544	0.189649493	1.469168248	2.802706778
F10	0.166390985	0.488912703	0.399724406	2.938336495	2.402320095
COL16A1	0.2288472	0.048904006	0.073301897	0.2136972	0.320309346
EPX	0.054909561	0.022001286	0.021985057	0.400682249	0.400386683
F2R	2.057134107	5.053054524	5.980408686	2.456356398	2.907155477
STRA6	10.95524695	15.66129057	28.94099413	1.429569834	2.641747307
ADRA2C	0.043509798	0.069734415	0.104524462	1.602728997	2.402320095
MEST	1.666433942	4.823867697	3.948840975	2.894724822	2.369635468
AKAP9	0.108129061	0.259952373	0.22729054	2.404093496	2.102030083
C17orf104	0.036391364	0.087488243	0.058282471	2.404093496	1.60154673
HIST1H3J	0.178226327	0.285648502	0.570875582	1.602728997	3.20309346
NHLH1	0.033291201	0.053356774	0.079976122	1.602728997	2.402320095
HIST1H3F	0.548447538	0.293004257	0.14639406	0.534242999	0.266924455
HIST1H3G	0.729697509	2.046637875	1.314725242	2.804775745	1.801740071
HIST1H4I	0.460498293	0.184513492	0.184377384	0.400682249	0.400386683
HIST1H4E	0.903895854	3.259575214	0.904769655	3.606140244	1.000966706
ANKRD6	0.029694034	0.095182979	0.083223671	3.205457995	2.802706778
AKAP7	0.116144764	0.255954298	0.395273941	2.203752371	3.403286801
GLTPD2	0.365631692	1.17201703	0.695371786	3.205457995	1.901836742
TGFB2	0.271859506	0.607710711	1.054115913	2.235385181	3.877428925
FES	0.029745874	0.071511863	0.047639408	2.404093496	1.60154673
PALM3	0.492237135	1.577845461	1.632991604	3.205457995	3.317489655
PALMD	0.264366747	0.37074472	0.952640323	1.402387873	3.603480143
DYX1C1	0.085491404	0.205529329	0.376525816	2.404093496	4.404253508
RGS11	0.176381334	0.282691478	0.621462486	1.602728997	3.523402806
FRS3	0.703744523	1.347227928	1.565388527	1.914370747	2.224370458
MFI2	2.579627076	5.52981767	7.38486554	2.143650034	2.86276478
OLR1	0.033860169	0.217074696	0.081342963	6.410915989	2.402320095
FRY	0.02384777	0.08918353	0.044558871	3.739700994	1.868471185
LOC102724549	0.344442793	0.184016151	0.091940205	0.534242999	0.266924455
DLX4	0.974835939	2.037910208	2.172167388	2.090516084	2.228238929
SLC14A1	0.019548459	0.062661764	0.26611605	3.205457995	13.61314721
LOC102724560	0.112341342	0.300087877	0.359839817	2.671214996	3.20309346
COMMD10	4.905729708	1.917696404	2.826515652	0.390909512	0.576166202
FYB	0.123110093	0.394624231	0.492916416	3.205457995	4.003866825
FYN	1.150611088	2.427461126	3.140209081	2.109714701	2.729166367

ATP6V1G2	0.797182011	1.320255615	2.127873737	1.656153297	2.66924455
ZNF585B	0.493867734	0.901471714	1.296282863	1.825330247	2.624757141
GK5	0.351066693	0.850248984	0.737171848	2.421901596	2.099805713
ADPRH	0.123861855	0.238220385	0.376904044	1.923274797	3.042938787
GRTP1	1.068910718	2.455549691	1.883101511	2.297244896	1.761701403
TIMP3	0.124232132	0.186666037	0.348186237	1.502558435	2.802706778
TMSB15B	1.53499431	0.205014991	0.40972752	0.13356075	0.266924455
MGLL	0.422106901	0.147070211	0.146961723	0.348419347	0.348162333
KAL1	0.026985171	0.216249579	0.194481054	8.013644987	7.206960285
RAB38	0.295806195	0.039508097	0.236873722	0.13356075	0.800773365
LOC102724620	0.383748577	0.922567459	1.434046319	2.404093496	3.73694237
RAB17	0.127852202	0.614737296	0.341268794	4.808186992	2.66924455
RALGPS1	0.184827302	0.370285095	0.401727261	2.003411247	2.173527705
VAV3	1.500554303	3.335480841	3.318715585	2.222832478	2.21165977
FAIM3	0.745362597	0.265469833	0.110530836	0.356161999	0.148291364
DNM1	3.857595723	7.381009054	8.191215037	1.913370292	2.123399035
FLJ22184	0.075424687	0.151106667	0.317089923	2.003411247	4.204060166
FIGNL2	0.090745829	0.058176389	0.014533369	0.641091599	0.160154673
SH3BGRL2	0.293386773	0.74941231	0.528606705	2.55434934	1.801740071
SRSF12	0.045302943	0.127064596	0.14510956	2.804775745	3.20309346
LOC102724369	0.121877231	0.586008515	0.4879802	4.808186992	4.003866825
GPR64	0.017364897	0.027831224	0.069526735	1.602728997	4.003866825
SH2D6	0.285879813	0.610917154	0.610466506	2.136971996	2.13539564
SLC12A8	5.655207669	11.20328074	13.70223378	1.981055585	2.422940868
PPP1R3G	0.392590711	0.275282226	0.078594046	0.701193936	0.200193341
PPP1R36	0.060635007	0.388725933	0.097109796	6.410915989	1.60154673
PPP1R1B	0.046199666	0.296182178	0.850895627	6.410915989	18.4177874
HNF4G	0.062320544	0.033294315	0.016634877	0.534242999	0.266924455
KIF1A	0.046119632	0.199576633	0.413631376	4.327368293	8.968661688
DOK6	0.086178449	0.191834304	0.222363643	2.226012496	2.580269732
DOK7	0.099601151	0.026605609	0.053171966	0.2671215	0.53384891
IL7	0.287256883	0.460394936	0.690082983	1.602728997	2.402320095
VCAN	0.102922259	0.599341908	0.642855746	5.82324869	6.246032247
TAS1R3	0.532659221	0.346819029	0.159952244	0.651108655	0.300290012
MAPK13	0.026840638	0.107545671	0.085973071	4.006822493	3.20309346
SERPINA3	3.375539374	8.587420375	23.29764791	2.544014282	6.901903765
SERPINB3	0.143824734	0.345768108	0.268732371	2.404093496	1.868471185
SEMA5B	0.086349264	0.207591705	0.15212162	2.404093496	1.761701403
CHRM4	0.058032823	0.418548997	0.929422779	7.212280488	16.0154673
ANKRD36B	0.02846381	0.091239548	0.034189592	3.205457995	1.201160048
RHOBTB1	0.093146932	0.238862863	0.25360458	2.564366396	2.722629441
SLC37A2	0.548186123	0.113891788	0.162582536	0.207761166	0.296582728
SYCP2	0.045925706	0.085874204	0.159363022	1.869850497	3.470017915
SEMA3A	0.029105632	0.05831055	0.104881566	2.003411247	3.603480143
SEMA3B	0.116382765	0.326427557	1.281448006	2.804775745	11.01063377
SEMA4D	0.655744843	1.780829382	1.225240344	2.715735246	1.868471185
SYCP3	0.057953867	0.278653028	0.092815826	4.808186992	1.60154673
SRPK3	0.085107077	0.204605371	0.272605922	2.404093496	3.20309346
SYDE2	0.108502039	0.173899364	0.275137552	1.602728997	2.535782323

OPN3	4.175452797	1.685999042	1.477400845	0.403788313	0.353830092
S1PR3	3.785025272	6.548568381	9.761205036	1.730125405	2.578900888
S1PR2	0.499172954	0.990524437	1.389518174	1.98433114	2.783640745
S1PR5	1.056383084	2.048099759	2.510482458	1.938785077	2.376488696
ATAD5	0.204624942	0.579393046	0.404183568	2.831487895	1.975240967
KMT2C	0.658586724	1.407381387	1.294475009	2.136971996	1.965534623
SGSM1	0.013794071	0.022108158	0.033137775	1.602728997	2.402320095
KMO	0.016177779	0.051857191	0.025909469	3.205457995	1.60154673
WDR86	0.057620686	0.138525516	0.207634998	2.404093496	3.603480143
WDR52	0.041520706	0.113128947	0.139644436	2.724639295	3.363248133
WDR31	0.161111506	0.272563532	0.43004601	1.691769497	2.66924455
LOC102724232	1.143519251	0.458187866	0.457849879	0.400682249	0.400386683
WDR17	0.045308966	0.108926992	0.099776087	2.404093496	2.202126754
ADTRP	0.917031046	0.404181869	0.367167018	0.440750474	0.400386683
LBH	0.405953226	1.231555335	0.882350584	3.033737031	2.173527705
WDR96	0.03171712	0.139793357	0.101592899	4.407504743	3.20309346
IFITM1	2.613986126	5.808186207	15.6990785	2.221965201	6.005800238
HS6ST3	1.047981764	1.478424993	2.762353116	1.41073542	2.635878993
TMEM74B	0.04362119	0.139825892	0.069861374	3.205457995	1.60154673
DOCK11	0.969833133	2.259986253	1.982415909	2.330283608	2.044079379
GPSM3	0.516316268	0.183892234	0.229695731	0.356161999	0.444874092
LXN	0.30534833	0.734085935	2.200633291	2.404093496	7.206960285
HLA-DPA1	0.576922241	0.115581251	0.346487973	0.200341125	0.600580024
NSUN7	0.069112102	0.239997268	0.092238551	3.472579494	1.334622275
FAM227A	0.033013828	0.152122633	0.072700635	4.607845867	2.202126754
FSCN2	0.206401415	0.330805534	0.557822551	1.602728997	2.702610107
DSC2	0.097455311	0.260324088	0.325165072	2.671214996	3.336555688
DSC3	0.036626047	0.078268836	0.068434713	2.136971996	1.868471185
MDK	91.06001321	176.3641459	189.8663738	1.936790252	2.085068595
ME3	1.73109929	0.47225243	0.796338117	0.272804936	0.460018742
MGP	89.76121512	191.4618716	322.5728017	2.133013366	3.59367686
COL9A3	0.137130276	0.274728338	0.46669366	2.003411247	3.403286801
MKX	0.069810585	0.279718623	0.260878134	4.006822493	3.73694237
MSC	0.206978096	0.497594694	0.43093062	2.404093496	2.082010749
HS3ST3B1	0.109889951	0.264185715	0.263990837	2.404093496	2.402320095
DYNLT3	0.807326655	1.447964631	2.001027112	1.793530068	2.478584225
MMP3	1.677745422	0.597549164	0.522469829	0.356161999	0.311411864
MX1	3.181966611	1.454248443	1.393456154	0.457028191	0.437922934
NKX2-6	0.282093325	1.507063841	0.978868892	5.342429991	3.470017915
FZD1	3.211843266	5.006466079	9.942815333	1.558751677	3.095672643
CDK18	0.476902812	2.223551902	3.298150148	4.662484356	6.915769971
WNT10A	0.179351967	0.02874526	0.057448111	0.1602729	0.320309346
DNAH10	0.024156003	0.062912731	0.058030452	2.604434621	2.402320095
DNAH14	0.08519827	0.151180067	0.214419873	1.774449961	2.51671629
BNC1	0.143542012	0.07189342	0.014368078	0.500852812	0.100096671
DTNA	0.175421471	0.572347337	0.431452298	3.262698316	2.459518193
NMU	0.522010933	0.083664206	0.08360249	0.1602729	0.160154673
NOG	6.979275132	3.500100012	1.58650307	0.501499073	0.22731631
NPY	0.608515601	1.828660499	3.410981601	3.00511687	5.605413555

COL3A1	36.01658643	72.77158344	76.09781903	2.020501959	2.112854842
CDKL5	0.0252222	0.14148523	0.114451174	5.609551491	4.537715735
TBX1	0.693109387	2.149285209	1.206572904	3.100932191	1.740811663
TBX6	0.121356388	0.259335202	0.453501827	2.136971996	3.73694237
HSD17B3	0.217697234	0.581516116	0.929739448	2.671214996	4.27079128
TEKT2	0.046655084	0.22432637	0.410961639	4.808186992	8.808507015
ODF3B	0.242195264	0.58226006	0.533344671	2.404093496	2.202126754
CHI3L1	2.550664197	6.949639902	3.825052857	2.724639295	1.49963012
DHRS3	82.19998505	205.9403872	313.8513828	2.505357965	3.818144037
DHRS9	0.059491749	0.166861014	0.285836447	2.804775745	4.80464019
TRPC6	0.018471853	0.059210748	0.05916707	3.205457995	3.20309346
NPIPB4	0.015355477	0.036916001	0.024592513	2.404093496	1.60154673
NPIPB5	0.669574411	1.609719486	1.090227287	2.404093496	1.628239176
PPL	0.355081242	0.569099003	1.038933158	1.602728997	2.92590268
PSD	0.061069666	0.195756249	0.146708886	3.205457995	2.402320095
PTGER1	0.539570484	1.056959763	1.392237388	1.958890997	2.580269732
PTGER2	0.034351687	0.11011289	0.385110826	3.205457995	11.21082711
MPP7	0.032722176	0.340891068	0.288233513	10.41773848	8.808507015
TRPV2	0.606781939	0.121563376	0.194357926	0.200341125	0.320309346
TRPV4	0.544434631	1.270933443	1.156264949	2.334409627	2.123790229
TDO2	0.100049541	0.280616526	0.600877557	2.804775745	6.005800238
AGAP2	2.716774359	4.535680255	6.345268258	1.669509372	2.335588981
WFIKKN1	0.043733154	0.140184788	0.07004069	3.205457995	1.60154673
TMEM40	9.382572808	3.032817783	4.35645961	0.323239462	0.464313968
TRMT10A	0.160566852	0.441163115	0.385732976	2.747535424	2.402320095
PLXDC2	0.229235329	0.545536466	0.461695161	2.379809723	2.014066342
TMEM56	0.237269349	0.610447011	0.529997141	2.572801812	2.233736229
SESN3	0.063152101	0.144593862	0.245628242	2.289612853	3.88947063
TMEM17	0.840857922	0.425579171	0.177193849	0.506124947	0.210729833
KIF21A	0.064141081	0.123360925	0.154087409	1.923274797	2.402320095
KIF21B	0.068565138	0.151100586	0.130399699	2.203752371	1.901836742
ST6GAL1	1.525359133	2.514988385	4.043476851	1.648784428	2.650835967
RSAD2	0.121287278	0.019439064	0.077698898	0.1602729	0.640618692
RAMP2	0.325575226	1.739362853	1.042847878	5.342429991	3.20309346
STAMBPL1	2.550061441	0.86043314	1.155354146	0.337416631	0.453069141
FRRS1L	0.573202249	0.229671966	0.286878183	0.400682249	0.500483353
SCN9A	0.061088434	0.223790181	0.083859412	3.663380565	1.37275434
SLC30A2	1.205712271	2.125673022	2.951678377	1.763001897	2.448078573
SCN5A	0.180322121	0.345203393	0.441213518	1.914370747	2.446807504
BCL2A1	0.271024976	0.072396598	0.072343194	0.2671215	0.266924455
COL12A1	4.429198971	11.39700272	7.399935902	2.57315212	1.670716522
REL	0.032867355	0.210709852	0.21055442	6.410915989	6.40618692
RET	0.014930281	0.047858389	0.035867314	3.205457995	2.402320095
RHD	0.029046091	0.139659036	0.139556015	4.808186992	4.80464019
MORN3	0.22070514	0.530595793	0.530204394	2.404093496	2.402320095
GCSAM	0.142065899	0.075897712	0.056881294	0.534242999	0.400386683
C4orf36	1.842898773	0.805545628	0.939109979	0.437107908	0.50958305
C4orf19	0.234173129	1.388669436	0.787582338	5.93009729	3.363248133
KLF7	1.505233659	2.882025038	3.154945629	1.91466954	2.095983976

KLF9	0.687033744	1.271541709	2.017247183	1.85077039	2.936169005
OXTR	21.09781218	37.32072022	55.43920565	1.76893793	2.627722968
BCL11B	0.074135416	0.01697414	0.050884857	0.228961285	0.68637717
KLLN	0.139430743	0.271356059	0.382808316	1.946170925	2.74550868
GALNT12	0.685275339	1.897082044	4.065740409	2.768350086	5.933002659
COL4A1	2.373695273	5.915617799	4.282003847	2.492155529	1.803939998
C5AR1	0.218254955	0.670456796	0.34954551	3.071897245	1.60154673
NR4A3	0.059721125	0.13161057	0.143469258	2.203752371	2.402320095
ARAP2	0.01132122	0.036289697	0.045328659	3.205457995	4.003866825
AGAP9	0.285520517	0.486212762	0.657332022	1.70289956	2.302223424
AGAP5	0.175581583	0.337691634	0.393682955	1.923274797	2.242165422
UNC5A	0.087872289	0.0528133	0.017591447	0.601023374	0.200193341
UNC5C	0.008574093	0.013741947	0.04119543	1.602728997	4.80464019
ADAMTS16	0.548079995	0.096077592	0.178298194	0.175298484	0.32531418
ADAMTS13	0.206484793	0.565354407	0.399589846	2.73799537	1.935202299
SHF	0.920075589	1.966175769	2.183028224	2.136971996	2.372661822
MMP24	0.196612472	0.063023302	0.125953625	0.320545799	0.640618692
MMP28	0.138242895	0.138478686	0.498155528	1.001705623	3.603480143
MMP11	1.846270246	3.95549268	3.771540912	2.142423456	2.042789197
BMP8A	0.075712926	0.02426946	0.048503116	0.320545799	0.640618692
BMP8B	0.022579429	0.090471762	0.126567035	4.006822493	5.605413555
SP9	0.117102659	0.328446697	0.56263614	2.804775745	4.80464019
SPDYE5	0.029175406	0.046760268	0.070088663	1.602728997	2.402320095
UNC79	0.009580767	0.046066121	0.030688094	4.808186992	3.20309346
VMO1	1.165564709	0.254738776	0.169700577	0.218553954	0.145595157
RAC2	1.240389181	0.316273954	0.316040651	0.254979613	0.254791525
MST1	0.133488223	0.406496348	0.299302679	3.045185095	2.242165422
GAB2	0.30480209	1.110261701	1.142725988	3.642565903	3.7490753
CNKSR1	0.077612072	0.041463706	0.02071656	0.534242999	0.266924455
ZFP14	0.045478278	0.109334032	0.081940036	2.404093496	1.801740071
TGM2	60.06027264	21.89792196	28.63067677	0.36459911	0.476699081
MSX2	0.1521289	0.030477675	0.030455193	0.200341125	0.200193341
GYLTL1B	1.534393294	2.860175858	3.979923716	1.864043508	2.593809378
SLIT3	1.400323554	2.798410397	2.838396442	1.998402719	2.02695758
TNIP3	0.10431696	0.501575451	0.222757982	4.808186992	2.13539564
BTG2	1.539239316	2.01386407	4.578161145	1.308350202	2.97430107
DENND2D	0.025847143	0.062138949	0.206977039	2.404093496	8.00773365
RARB	1.981862739	3.467185045	5.565756347	1.749457708	2.808346027
SYNE4	0.110567403	0.044302396	0.044269716	0.400682249	0.400386683
TRO	2.423556349	5.170142614	5.05925464	2.133287562	2.087533324
LANCL3	0.025567882	0.040978387	0.122844475	1.602728997	4.80464019
TUB	0.996011507	2.192302159	2.03116909	2.201081156	2.039302836
VNN1	0.177299031	0.390723159	0.319446768	2.203752371	1.801740071
TENM2	16.35888829	37.42895669	31.87343313	2.287989014	1.948386257
GOLGA6L9	0.105126866	0.336979754	0.273594082	3.205457995	2.602513436
HIST1H2AK	0.1852004	0.593652104	0.296607096	3.205457995	1.60154673
GLP2R	0.032994649	0.052881481	0.184948654	1.602728997	5.605413555
RBKS	0.677260042	0.314213352	0.342525349	0.463947868	0.505751599
HIST1H2BN	0.379475208	1.672538779	0.759684098	4.407504743	2.001933413

HIST1H2BL	2.632871035	1.507063841	0.752976071	0.572403213	0.285990488
GBP2	0.26796477	0.330365313	0.775785798	1.23286846	2.895103704
COL1A1	5.18886089	12.23261878	9.990659792	2.357476725	1.92540521
ADSSL1	0.56418665	1.167974476	1.129464106	2.070191622	2.001933413
GAREM	0.024212643	0.009701576	0.00969442	0.400682249	0.400386683
MXRA5	0.182958615	0.353212571	0.419546741	1.930559929	2.293123727
NDRG4	0.239303888	0.019176964	0.134139726	0.08013645	0.560541356
MMRN2	1.356243498	0.590385151	0.616765541	0.43530911	0.454760183
GSDMB	0.186280286	0.721512306	0.944732513	3.873261744	5.071564645
TMEM173	1.410077532	3.295792716	2.57195854	2.337313121	1.823983776
VTN	0.050770074	0.122056005	0.081310646	2.404093496	1.60154673
CDRT4	0.375145727	0.655916656	0.98314922	1.748431633	2.620712831
IFNLR1	0.018670213	0.044884939	0.059802439	2.404093496	3.20309346
WAS	0.046527681	0.074571264	0.111774383	1.602728997	2.402320095
LILRB3	0.390661834	0.111808045	0.022345114	0.286201607	0.057198098
TJP3	0.21823166	0.437207762	0.699016402	2.003411247	3.20309346
AMY2B	0.075996596	0.243603896	0.152140125	3.205457995	2.001933413
LMX1B	0.234648812	0.763909361	0.692883162	3.255543276	2.952851784
SEL1L3	0.093638365	0.300153845	0.599864867	3.205457995	6.40618692
UPK1B	0.041395619	0.099518939	0.165742546	2.404093496	4.003866825
PDE5A	0.134084136	0.429801066	0.819924036	3.205457995	6.114996606
SAMD5	0.161654998	0.4642014	0.183386108	2.87155612	1.134428934
LIMS2	0.109127478	0.116601182	0.436931887	1.068485998	4.003866825
LEAP2	0.361494417	0.193125861	0.0964917	0.534242999	0.266924455
HAND2	0.179882146	0.201811632	0.604988291	1.121910298	3.363248133
KIAA1549L	0.234568051	0.405320035	0.833521575	1.7279422	3.553431807
SYT15	0.030933981	0.099157577	0.099084433	3.205457995	3.20309346
SYT17	0.279135597	0.603961266	0.894097405	2.163684146	3.20309346
SYT12	7.142709502	19.63567787	24.82952181	2.749051724	3.476204906
PIK3AP1	0.017707791	0.198665535	0.042539783	11.21910298	2.402320095
KCTD12	0.573684742	1.28067806	1.137540761	2.232372532	1.98286738
LPAR1	0.827542876	2.258340507	3.779034485	2.728970995	4.566572433
IL11	0.035825141	0.057417992	0.086063455	1.602728997	2.402320095
IL1B	0.704899869	0.928019986	3.749660625	1.316527391	5.319423068
MGAT3	0.015799737	0.139274835	0.265692187	8.815009485	16.81624067
REM2	0.229134438	0.110172123	0.036696951	0.480818699	0.160154673
IL32	0.220895897	0.118012086	0.058962517	0.534242999	0.266924455
CACNA1A	0.029560092	0.126338176	0.11835467	4.273943993	4.003866825
CACNA1H	0.042132633	0.362958662	0.210866815	8.614668361	5.004833531
CACNA1G	0.033897218	0.101865103	0.061073977	3.00511687	1.801740071
SNTG1	0.025071273	0.100456139	0.040152815	4.006822493	1.60154673
TMEM249	0.159935264	0.341777181	0.298834433	2.136971996	1.868471185
RERG	0.109925399	0.322997812	0.381443104	2.938336495	3.470017915
TMEM221	0.044141028	0.106119159	0.600898313	2.404093496	13.61314721
ZNF804A	0.054552092	0.014572037	0.029122575	0.2671215	0.53384891
NYNRIN	0.130114065	0.225915717	0.32125829	1.736289747	2.469051209
PLEKHS1	0.296836879	0.158583024	0.079233022	0.534242999	0.266924455
PCDHA1	0.015732629	0.02521514	0.06299135	1.602728997	4.003866825
FAM211B	0.298770185	1.060305487	1.367126894	3.548899923	4.5758478

FAM211A	0.332427821	0.145306829	0.145199642	0.437107908	0.436785472
FAM214A	0.397925277	0.768218593	0.984911885	1.930559929	2.475117674
FAM212A	2.457466852	1.378528684	0.983937	0.560955149	0.400386683
SALL2	2.938833074	5.66307411	6.420671248	1.926980529	2.18476895
SEC16B	0.02117102	0.067862815	0.084765944	3.205457995	4.003866825
LRFN1	3.80227289	7.841247518	8.154843652	2.062252696	2.144728663
PIK3C2A	0.472731128	0.959165177	0.934294842	2.028986709	1.976376816
PLEKHH2	0.036752452	0.107991072	0.137341796	2.938336495	3.73694237
LOC101928083	0.161830472	0.352002672	0.370255805	2.175132211	2.2879239
P2RY2	0.009751853	0.015629577	0.039045119	1.602728997	4.003866825
P2RX6	0.751143432	1.329283459	2.155359424	1.769679935	2.869437891
TLL2	0.012581921	0.040330818	0.030225801	3.205457995	2.402320095
KRT9	0.409220099	0.119248894	0.148951162	0.291405272	0.363987893
SPTBN2	0.19153668	0.520533663	0.473469587	2.717670909	2.471952562
TLR5	0.019661247	0.063023302	0.062976812	3.205457995	3.20309346
HOXA11	0.096334924	0.257331293	0.179999029	2.671214996	1.868471185
EFNA2	0.039753702	0.127428823	0.063667412	3.205457995	1.60154673
MEGF11	0.086431029	0.230875861	0.219170275	2.671214996	2.535782323
LOC730268	0.038015254	0.761601874	0.395740837	20.03411247	10.41005375
IL22RA1	0.316378807	0.59926394	0.806106388	1.89413427	2.547915252
LYPD6B	0.054159049	0.477412531	0.21684562	8.815009485	4.003866825
TMC3	0.077377097	0.020669086	0.02065384	0.2671215	0.266924455
MYRF	0.430989127	0.874961111	1.046877995	2.030123397	2.429012541
MYT1	0.015391542	0.037002706	0.024650274	2.404093496	1.60154673
EDIL3	1.503391486	3.313102552	5.202463534	2.203752371	3.460484899
KSR2	0.080143165	0.156545725	0.184507471	1.953325966	2.302223424
ATP9A	0.40460668	0.855626549	1.250993252	2.114711872	3.091874937
NOVA1	0.616038654	1.051042564	1.6231403	1.706130868	2.634802685
NOVA2	0.232917051	0.471072722	0.843752772	2.022491354	3.622546175
PCDH10	0.444536183	0.928756879	1.029778269	2.089271729	2.316522949
PCDH18	0.288493682	0.762922362	0.612197849	2.644502846	2.122049417
RGMA	0.871193703	2.221366333	2.304288779	2.549796132	2.644978691
GGT1	0.031847545	0.229693431	0.204021329	7.212280488	6.40618692
TMEM191C	0.651814722	0.156702353	0.260977934	0.24040935	0.400386683
TMEM191B	0.447035449	0.204707622	0.153417463	0.457922571	0.343188585
SYTL5	0.035371469	0.08503632	0.113298123	2.404093496	3.20309346
METTL7B	1.4707814	2.810583999	3.986273318	1.910946112	2.710309851
METTL7A	0.022809152	0.03655689	0.073059847	1.602728997	3.20309346
INHA	0.059533322	0.238539455	0.095345398	4.006822493	1.60154673
TNIK	0.278789442	0.776062603	1.151485349	2.783687206	4.130304725
CEP350	0.251100683	0.477905036	0.573063006	1.903240684	2.28220409
TNNI3	1.717991852	3.563321052	4.774564993	2.074119879	2.77915462
HSF2BP	1.525294504	0.862811905	0.538859653	0.565669058	0.353282367
TNS1	0.911944362	0.451767172	0.398324048	0.495388963	0.436785472
TMEM52B	0.030545781	0.269261352	0.244602481	8.815009485	8.00773365
EBF3	0.199903444	0.026699254	0.053359118	0.13356075	0.266924455
RUNX2	0.245466405	0.614712696	0.565118508	2.504264058	2.302223424
IL17RD	0.400559582	1.401413826	2.167068586	3.498640128	5.410102978
RHOJ	0.047080511	0.282964874	0.207354505	6.01023374	4.404253508

RHOV	0.299796543	0.720738918	0.560161201	2.404093496	1.868471185
KIAA0754	3.478975856	6.710590987	7.380114941	1.928898407	2.121346985
ARHGAP44	3.723154666	5.983248827	10.14638794	1.607037409	2.725212581
ARHGAP42	0.215131778	0.330431358	0.617307276	1.535948622	2.869437891
CMPK2	0.056530978	0.090603838	0.158439756	1.602728997	2.802706778
PBX1	2.383166498	4.514037333	5.185385988	1.89413427	2.175838739
SNX10	0.063128703	0.177061854	0.075827675	2.804775745	1.201160048
SLC18A3	0.105610146	0.02821074	0.028189931	0.2671215	0.266924455
SNX32	0.355894242	1.354704802	0.926219547	3.806481369	2.602513436
SNX22	0.302512536	0.167831174	0.093170762	0.554790807	0.307989756
NOXA1	0.667569858	1.646051645	2.508377066	2.465736919	3.757475021
ZNF891	0.665852938	0.296439392	0.177732433	0.445202499	0.266924455
TRIM74	0.063105322	0.101140729	0.252665304	1.602728997	4.003866825
ZNF878	0.193398829	0.542440345	0.271020104	2.804775745	1.401353389
TM6SF1	0.401639423	0.035762175	0.214414768	0.0890405	0.53384891
TM6SF2	0.114891685	0.046035059	0.0460011	0.400682249	0.400386683
B4GALNT4	6.980135532	15.75309311	14.81318008	2.256846309	2.122190897
ECM2	0.051788562	0.145255302	0.248825406	2.804775745	4.80464019
PCLO	0.075584548	0.188442407	0.131139868	2.493133996	1.735008958
MCF2L2	0.067977007	0.122567311	0.231345251	1.803070122	3.403286801
SPINK6	0.581119947	0.465688895	0.093069075	0.801364499	0.160154673
PCP2	0.085534321	0.205632506	0.136987213	2.404093496	1.60154673
SYNGR3	3.32295209	5.625367452	7.716701427	1.692882503	2.322242759
SARDH	0.050349991	0.100871738	0.161275726	2.003411247	3.20309346
GJA3	0.130913844	0.144250847	0.406225239	1.101876186	3.10299679
ARMC4	0.023849996	0.305800636	0.248279736	12.82183198	10.41005375
AKAP17A	0.312249912	1.021756141	0.687613884	3.27223837	2.202126754
GJB2	0.255503552	0.175501265	0.029228634	0.686883856	0.114396195
LOC101928485	0.095685718	0.230037412	0.153245149	2.404093496	1.60154673
GLT8D2	0.039977562	0.25629279	0.064025933	6.410915989	1.60154673
ZNF732	0.660404529	0.34243954	0.27997113	0.51852997	0.42393884
ZNF813	0.101709867	0.260821364	0.276918277	2.564366396	2.722629441
ZNF805	0.067305696	0.276424024	0.229060585	4.106993056	3.403286801
VWA2	0.029090724	0.011656137	0.011647538	0.400682249	0.400386683
FAM86B2	0.476200023	1.030346441	1.029586397	2.163684146	2.162088086
IQCG	0.565935679	1.239623415	1.208496581	2.190396296	2.13539564
SLC16A9	0.02136749	0.102738889	0.359320861	4.808186992	16.81624067
KIAA0408	0.04194593	0.058824464	0.109164847	1.402387873	2.602513436
LOC101928170	0.433879217	1.060470973	1.007572869	2.444161721	2.322242759
NRARP	0.650073897	1.562838428	1.119208003	2.404093496	1.721662735
IQUB	0.024236752	0.038844946	0.058224437	1.602728997	2.402320095
CKMT2	0.18723557	0.337598861	0.412316457	1.803070122	2.202126754
C2orf88	0.020361421	0.065267679	0.04891465	3.205457995	2.402320095
C2orf72	0.047580108	0.171580544	0.114302651	3.606140244	2.402320095
ZNF610	0.150516226	0.271391311	0.331455809	1.803070122	2.202126754
SRGAP2D	0.026722768	0.17131742	0.149792166	6.410915989	5.605413555
THAP9	0.105488093	0.338137652	0.118260878	3.205457995	1.121082711
ZNF714	0.154754195	0.341039924	0.348533542	2.203752371	2.252175089
ZNF704	0.035359235	0.165291209	0.188764892	4.674626242	5.3384891

C2orf48	0.936177848	0.187554923	0.655958	0.200341125	0.700676694
TMEM150C	0.135368937	0.506239347	0.758798873	3.739700994	5.605413555
ESYT3	0.034462858	0.069043277	0.124186223	2.003411247	3.603480143
HIST4H4	0.413554292	0.994223184	0.827908156	2.404093496	2.001933413
IRF5	0.114775593	0.229942715	0.298705024	2.003411247	2.602513436
MILR1	0.630334886	0.310848	0.271791363	0.493147384	0.431185658
PIH1D2	0.252234446	0.909592787	0.555452222	3.606140244	2.202126754
GLI2	0.176198933	0.292484823	0.423286238	1.659969319	2.402320095
ZNF596	0.394669285	0.27109196	0.112871661	0.686883856	0.285990488
SULF1	0.014904161	0.083605658	0.155153117	5.609551491	10.41005375
SULF2	0.020026371	0.064193693	0.064146339	3.205457995	3.20309346
PHLDA2	11.85282563	4.229771242	4.968168853	0.356857628	0.419154808
C19orf73	0.57252812	1.284650387	1.283702753	2.243820596	2.242165422
SMPDL3A	0.113388	0.666344199	0.696118694	5.87667299	6.139262465
ZNF501	0.075569649	0.141303946	0.221885261	1.869850497	2.936169005
EDNRB	0.019054391	0.32065977	0.534038721	16.82865447	28.02706778
C19orf38	0.058270988	0.233481505	0.18664742	4.006822493	3.20309346
KRT32	0.084979735	0.374548584	0.476346558	4.407504743	5.605413555
CA12	10.50167805	19.61366814	23.92406718	1.867669913	2.278118512
CA11	8.324307798	16.75836314	18.6319586	2.013183984	2.238259211
KRT16	1.239700003	0.119214189	0.119126249	0.09616374	0.096092804
KRT86	0.218534889	0.058375367	0.145830765	0.2671215	0.667311138
ISLR	0.074241555	0.535450917	0.237802639	7.212280488	3.20309346
ZNF493	0.015048964	0.108537348	0.060254047	7.212280488	4.003866825
ZNF492	0.02006883	0.112577136	0.048211754	5.609551491	2.402320095
ZNF483	0.137075115	0.45769638	0.311003954	3.339018744	2.268857868
ISPD	0.200488486	0.024717593	0.07409808	0.123286846	0.369587707
ZNF469	0.109692277	0.305076841	0.366855554	2.781206201	3.344406407
NUDT13	0.409086119	0.163913546	0.136493862	0.400682249	0.333655569
NAAA	0.868841385	0.109935575	0.29294528	0.126531237	0.337167733
ZNF417	0.380403119	0.87097587	0.681761153	2.289612853	1.792207055
ZNF404	0.075059193	0.481198181	0.360632416	6.410915989	4.80464019
EGR3	0.019463602	0.093584636	0.062343735	4.808186992	3.20309346
LOC101060389	0.025560211	0.102415229	0.143275555	4.006822493	5.605413555
MAP1LC3C	0.071469953	0.229093933	0.286156175	3.205457995	4.003866825
AKR7A3	0.068648013	0.275060403	0.164914501	4.006822493	2.402320095
GNG2	0.241151319	0.421636595	0.702209285	1.748431633	2.911903146
ZNF396	0.061113475	0.122435423	0.195752172	2.003411247	3.20309346
RBPMS2	1.36580656	3.42034028	3.93048982	2.504264058	2.877779281
RNLS	0.282093325	0.028257447	0.141183013	0.100170562	0.500483353
TUBB4A	13.72021462	30.63054107	32.55936982	2.232511803	2.37309479
ZNF33B	0.209901899	0.437340619	0.403401241	2.083547697	1.921856076
SERF1B	1.164561929	0.39483171	0.430407773	0.339038826	0.369587707
PHIP	0.505485967	1.095423572	0.99769644	2.167070194	1.973737167
CEACAM19	0.037896879	0.212584495	0.060693623	5.609551491	1.60154673
MAATS1	0.057653181	0.169404447	0.184668528	2.938336495	3.20309346
RAET1E	1.095849916	0.520400129	0.390012189	0.474882666	0.355899273
ROBO1	7.798483902	17.38179139	18.10633148	2.228868023	2.321775835
CBY3	0.071590071	0.172109223	0.171982266	2.404093496	2.402320095

YBX2	0.269084599	1.121301195	0.603332185	4.167095393	2.242165422
THBS2	2.057012439	6.465972714	5.22000245	3.143380463	2.537662072
TCF7L1	1.45977012	3.345650738	2.524921267	2.291902466	1.729670468
ZNF284	0.231500501	0.463790706	0.834207457	2.003411247	3.603480143
FERMT3	0.066765035	0.133757821	0.160390984	2.003411247	2.402320095
LRRC6	0.049907548	0.119982411	0.099911588	2.404093496	2.001933413
ZNF234	0.425960921	0.712382525	1.02329448	1.672412867	2.402320095
ZNF233	0.091506105	0.342205474	0.293102608	3.739700994	3.20309346
ZNF221	0.10534895	0.393973573	0.421803166	3.739700994	4.003866825
ATXN1	0.816999134	1.694554145	2.007591653	2.074119879	2.45727513
HUS1B	0.249099954	0.133079906	0.066490869	0.534242999	0.266924455
C9orf47	0.052201093	0.069720172	0.153271232	1.335607498	2.936169005
CXXC5	0.465107466	0.99392163	0.900077037	2.136971996	1.935202299
ADHFE1	0.674344466	1.116817811	1.763990485	1.656153297	2.615859659
LRRN1	0.021097619	0.084534413	0.033788822	4.006822493	1.60154673
FAM57B	0.201590592	0.581571157	0.839427559	2.884912195	4.164021498
CD37	1.553862202	3.627350882	2.596782186	2.334409627	1.671179197
CD72	0.220134843	0.44102062	0.749182005	2.003411247	3.403286801
RUNDC3A	0.254443124	0.055609551	0.16670559	0.218553954	0.655178208
RASGRP3	0.159602343	0.014211072	0.127805306	0.0890405	0.800773365
ZNF135	0.318901224	0.109524051	0.05472163	0.343441928	0.171594293
ZNF121	0.439890796	0.822530024	0.978480092	1.869850497	2.224370458
ZNF112	0.068427457	0.32901201	0.273974426	4.808186992	4.003866825
ATP6AP1L	0.224781489	0.450329762	1.349992718	2.003411247	6.005800238
GPR4	0.16770115	0.627152157	0.738598378	3.739700994	4.404253508
CDH4	0.379329547	0.041928446	0.125692551	0.110533034	0.331354496
CDH3	14.36472517	27.20581533	30.71159767	1.893932185	2.13798714
CDH1	0.035386162	0.141786068	0.042504444	4.006822493	1.201160048
LOC101060321	0.181646448	0.058226006	0.11636611	0.320545799	0.640618692
LSMEM1	0.079955124	0.032036599	0.032012967	0.400682249	0.400386683
LOC100653515	0.26247592	0.120193648	0.030026246	0.457922571	0.114396195
PTPN13	0.923627084	2.172733482	2.051837848	2.352392561	2.221500303
FAM63B	0.169942518	0.367701932	0.353822149	2.163684146	2.082010749
LOC101928641	0.181517438	0.549521716	0.468363951	3.027376995	2.580269732
RDH10	10.40449587	20.91339144	30.53788221	2.010034094	2.935066012
NEBL	0.044104465	0.070687505	0.155397795	1.602728997	3.523402806
PKIA	0.567812701	1.088809679	1.575173603	1.917550765	2.774107729
KCNC1	1.028679002	2.151344174	2.290395538	2.091365887	2.226540576
KIAA2018	0.186443356	0.512971197	0.527522687	2.751351445	2.829399223
PKLR	0.080092934	0.256734536	0.192408865	3.205457995	2.402320095
NEO1	0.138205517	0.29534132	0.322791283	2.136971996	2.335588981
KCNJ4	0.99012708	1.917510671	2.378602182	1.936630872	2.402320095
COL26A1	0.337061065	1.035416957	0.472341666	3.071897245	1.401353389
KCNK1	0.197112874	0.379102223	0.631370958	1.923274797	3.20309346
SLC23A3	0.0352325	0.084702223	0.084639742	2.404093496	2.402320095
SOAT2	0.32876868	0.065865887	0.098725951	0.200341125	0.300290012
KCNQ2	0.110843132	0.688399572	0.451197826	6.210574865	4.070597939
NTNG2	0.156795431	0.104708577	0.041852535	0.667803749	0.266924455
KCNS2	0.026891472	0.064649614	0.04306795	2.404093496	1.60154673

PLAT	61.29648803	138.756123	248.9489817	2.263687977	4.061390624
PLB1	0.049645795	0.013261459	0.013251677	0.2671215	0.266924455
ADAM12	7.49419861	14.86530869	16.00873783	1.983575492	2.13615073
CARD14	0.061201282	0.245222672	0.147025069	4.006822493	2.402320095
PIBF1	0.355651379	0.789248462	0.722944077	2.219163227	2.032732388
GRM2	0.25362365	0.121946994	0.040619013	0.480818699	0.160154673
ELF3	0.157374725	0.44140081	0.609103857	2.804775745	3.870404598
HS3ST1	0.346838409	1.007546955	1.527564279	2.904946308	4.404253508
ST3GAL6	0.095426698	0.286768379	0.267453052	3.00511687	2.802706778
LRRC37A2	0.049386773	0.13192269	0.171372989	2.671214996	3.470017915
PLLP	0.569086067	1.003299815	1.321555998	1.763001897	2.322242759
PLP1	0.02717454	0.065330136	0.369931019	2.404093496	13.61314721
IL13RA2	3.722892245	1.541420067	1.391223377	0.414038324	0.373694237
APOA1	0.091114636	0.146032068	0.364810866	1.602728997	4.003866825
CEBPA	0.71236338	1.530950029	1.607608204	2.149113883	2.256724938
CEBPE	0.385484996	0.17652228	0.176392067	0.457922571	0.45758478
APOBR	0.06739888	0.01800369	0.035980819	0.2671215	0.53384891
NIPAL1	0.116488857	0.062233356	0.031093725	0.534242999	0.266924455
SLC22A4	1.632331996	0.622901387	0.466831423	0.381602142	0.285990488
FAM43B	0.032994649	0.158644443	0.079263709	4.808186992	2.402320095
FAM46B	8.899604752	4.859995217	3.879380927	0.54609113	0.435904856
PMCH	0.675235278	0.36073972	0.180236808	0.534242999	0.266924455
NGEF	0.535126785	0.879104796	1.671209578	1.642797222	3.123016124
GRIN2A	0.058017015	0.158075438	0.102208656	2.724639295	1.761701403
PRKAR2B	0.442133707	0.988339135	1.024878383	2.235385181	2.318028162
GLYATL2	0.061733467	0.346297061	0.346041612	5.609551491	5.605413555
GRIN3A	0.010962834	0.026355678	0.017557491	2.404093496	1.60154673
AADAT	0.549390698	0.234806507	0.087987488	0.427394399	0.160154673
FAM13C	0.024011326	0.057725472	0.03845526	2.404093496	1.60154673
LAG3	0.042767161	0.137088337	0.205480819	3.205457995	4.80464019
ST6GALNAC2	0.121414039	0.291890702	0.486125644	2.404093496	4.003866825
SLC47A2	0.074382	0.039738063	0.019854375	0.534242999	0.266924455
CECR6	0.016953668	0.095102476	0.027152092	5.609551491	1.60154673
BAIAP3	0.054598708	0.145844888	0.116589843	2.671214996	2.13539564
GREB1L	0.079147309	0.380555059	0.324817665	4.808186992	4.103963496
CRIP3	0.160942413	0.988797113	0.42959466	6.14379449	2.66924455
CRIP1	20.7655949	8.960436447	8.243205535	0.431503961	0.396964574
FAM171B	0.093515021	0.312248408	0.436825306	3.339018744	4.671177963
RTL1	0.040635432	0.048845689	0.16269886	1.202046748	4.003866825
FAM174B	1.392876015	2.777524389	6.873841249	1.994093055	4.934998645
CHD2	2.256264596	4.082784655	5.595117192	1.809532739	2.479814292
CHDH	0.558074273	1.032048254	1.443801736	1.849302689	2.587113949
LOC101928991	0.064686548	0.31102502	0.41439412	4.808186992	6.40618692
RNF125	0.014468781	0.104352909	0.104275932	7.212280488	7.206960285
RNF157	0.980604705	1.792225153	2.617473765	1.827673418	2.66924455
RNF152	0.01894844	0.007592303	0.007586703	0.400682249	0.400386683
LOC102723563	1.959275105	1.046729008	0.813522017	0.534242999	0.415215819
LOC102723565	0.17529256	0.561892938	0.561478453	3.205457995	3.20309346
IQCA1	0.076451257	0.081687098	0.285693942	1.068485998	3.73694237

CHRD	0.169368159	0.329619388	0.484376825	1.946170925	2.859904875
LOC102723588	0.112242667	0.269841866	0.179761876	2.404093496	1.60154673
KCNAB3	0.060960418	0.146554545	0.122038698	2.404093496	2.001933413
B3GNT8	0.136380231	0.072860184	0.036403219	0.534242999	0.266924455
B3GNT7	0.070503877	0.169497911	0.301107341	2.404093496	4.27079128
OVOS2	0.073919466	0.236945742	0.251569142	3.205457995	3.403286801
FAM186B	0.027375381	0.175501265	0.153450329	6.410915989	5.605413555
PTCH2	0.03964271	0.127073042	0.174596545	3.205457995	4.404253508
POLK	0.260925526	0.62728936	0.522355528	2.404093496	2.001933413
RNF207	1.308668657	2.69916614	2.61127778	2.0625283	1.995369696
NRIP1	0.284484561	0.736537288	0.709708476	2.589023765	2.494717022
POLN	0.029386749	0.070648491	0.094128502	2.404093496	3.20309346
LCA5	0.018053016	0.144670464	0.144563747	8.013644987	8.00773365
CCDC169-SOHLH2	0.257183952	0.077286783	0.025743257	0.300511687	0.100096671
ACHE	0.432155145	0.669539996	1.153527765	1.549304697	2.66924455
YPEL3	3.057905856	6.104747394	7.646784949	1.996381734	2.500660684
CRLF1	0.047224049	0.491967791	0.378157605	10.41773848	8.00773365
YPEL2	0.03256582	0.078291275	0.104311364	2.404093496	3.20309346
LCN2	0.207280254	0.747481664	0.580945771	3.606140244	2.802706778
TTC39A	0.087826994	0.258065262	0.117215863	2.938336495	1.334622275
KALRN	0.010609238	0.017003734	0.025486787	1.602728997	2.402320095
EPGN	0.123154585	0.024672928	0.073964184	0.200341125	0.600580024
SCNN1D	0.306848077	0.692982892	1.005200865	2.25839086	3.275891039
SCNN1A	0.04894696	0.176509603	0.137183977	3.606140244	2.802706778
HHIPL2	0.463360753	0.954825063	1.033630788	2.060651568	2.230725803
ADD2	2.419879959	0.928166922	1.093104082	0.383559076	0.451718308
AGPAT4	1.070443741	2.304837907	2.095335823	2.153161178	1.957446003
LDHD	0.287811433	0.626027919	1.28405678	2.175132211	4.461451605
C10orf54	4.389877946	10.43927401	8.988300908	2.378032862	2.047505881
TP53TG5	0.450071917	1.030490445	0.97824378	2.289612853	2.173527705
ICOSLG	0.154754195	0.041338173	0.082615358	0.2671215	0.53384891
MCF2L	0.02447348	0.068642623	0.19597711	2.804775745	8.00773365
PGAP1	0.276282831	0.616338783	0.723514372	2.230825496	2.618745329
NYAP1	0.303175033	0.60738427	0.867051754	2.003411247	2.859904875
RHBDL1	0.473582247	1.349376	1.05342236	2.849295995	2.224370458
VAMP7	0.73690118	1.258078077	2.668236829	1.707254802	3.620888259
LRRC29	0.637926498	1.161844656	2.229096213	1.821282952	3.494283775
LEPR	0.315219377	1.023717504	0.983106674	3.247635074	3.118801527
IFI16	0.079519774	0.488553147	0.169806178	6.14379449	2.13539564
SH3BP1	8.59859002	16.21942356	18.88221958	1.886288743	2.195966959
IFI27	0.116861707	0.374595292	0.842217679	3.205457995	7.206960285
PDZRN3	1.760761317	3.609564584	4.951292686	2.050002206	2.812018096
HIST2H4B	0.215131778	0.517196909	0.344543596	2.404093496	1.60154673
AKR1C3	1.252826238	2.733030725	3.121159635	2.181492246	2.491294913
AKR1C2	0.047250241	0.113593997	0.359448976	2.404093496	7.607346968
CLGN	0.07754143	0.124277898	0.227674743	1.602728997	2.936169005
AFF3	0.036025873	0.129914352	0.07933354	3.606140244	2.202126754
CRMP1	0.320701957	0.624140825	0.917177091	1.946170925	2.859904875
PTHLH	0.360411144	0.902564675	1.406962268	2.504264058	3.903770155

PRTG	0.040525894	0.086602701	0.086538818	2.136971996	2.13539564
CLMN	0.080180879	0.165989784	0.278229087	2.070191622	3.470017915
ACOT6	0.424263866	0.271992	0.067947841	0.641091599	0.160154673
C17orf72	0.027410613	0.043931784	0.15364782	1.602728997	5.605413555
GYG2	0.777896233	2.131552032	1.768285012	2.740149576	2.273163101
C2orf27A	0.298710323	0.095750339	0.047839854	0.320545799	0.160154673
PSD3	0.481105858	0.911279092	1.074049139	1.89413427	2.232459078
NTSR1	1.113353811	0.165222633	0.561342567	0.148400833	0.504190637
DNASE1L2	0.145379154	0.388338976	0.232831509	2.671214996	1.60154673
MYH14	0.024650516	0.029631073	0.069088169	1.202046748	2.802706778
NCAM2	0.11908745	0.310155476	0.238405144	2.604434621	2.001933413
LGI4	0.058997357	0.496423072	0.094487025	8.414327236	1.60154673
LYPD6	0.228397277	0.599005535	0.315908605	2.62264745	1.383153994
JAK3	0.093361298	0.423959768	0.535788891	4.541065492	5.738875783
CCDC7	0.151198907	0.10770261	0.026905791	0.712323999	0.177949637
ARL17A	0.145379154	0.310671181	0.362182347	2.136971996	2.491294913
PTK6	0.033606384	0.053861927	0.134555487	1.602728997	4.003866825
NEK10	0.085499984	0.191846625	0.191705108	2.243820596	2.242165422
SLFN13	0.040237187	0.112856286	0.112773037	2.804775745	2.802706778
C6orf165	0.038461483	0.061643334	0.277190378	1.602728997	7.206960285
PTX3	0.395629338	0.951129919	1.337639843	2.404093496	3.381043097
BNIPL	0.105960428	0.028304308	0.056566859	0.2671215	0.53384891
MRGPRF	0.149263573	0.478458113	0.328697307	3.205457995	2.202126754
UBN2	0.076675325	0.132342826	0.188921717	1.726015843	2.463918046
LHX6	0.165974197	0.475020818	0.360749515	2.862016067	2.173527705
LHX9	0.066582403	0.146731128	0.159952244	2.203752371	2.402320095
IFIT2	0.609386153	1.074348944	1.502979017	1.763001897	2.466381964
GDPD1	0.181535857	0.27017044	0.539942293	1.488248355	2.97430107
GDPD3	0.129373097	0.570212537	0.880587505	4.407504743	6.806573603
GDPD5	0.617334668	1.269038918	1.246609533	2.055674149	2.019341529
ZDHHC19	0.572186312	1.426537146	1.323664502	2.493133996	2.313345277
KISS1R	0.157277879	0.042012303	0.041981312	0.2671215	0.266924455
COLQ	0.081549634	0.174269284	0.261211099	2.136971996	3.20309346
ARHGDIG	0.361750251	0.724734522	0.724199915	2.003411247	2.001933413
LIG4	0.308947178	0.727264194	0.896812932	2.354008215	2.902803448
ZSCAN1	0.285400952	0.781426486	0.876075676	2.73799537	3.069631233
NOS3	0.038374858	0.015376124	0.015364782	0.400682249	0.400386683
SAMD14	0.77424735	1.340981959	1.97998932	1.731981336	2.557308488
SAMD11	8.439163117	18.44412469	16.42720191	2.185539542	1.946543951
NOX5	0.028069912	0.134965388	0.112388191	4.808186992	4.003866825
NOX1	0.168430574	0.107979426	0.053949887	0.641091599	0.320309346
C20orf195	0.912773402	1.706749799	3.228250444	1.869850497	3.536749029
FOXO4	1.119610545	2.120692702	2.934177721	1.89413427	2.620712831
FOXO1	0.66811577	2.117821292	2.544266513	3.169841795	3.808122225
CPA4	7.258120059	30.58456865	29.83549402	4.213841656	4.110636607
C7orf61	0.084852773	0.33999	0.475634885	4.006822493	5.605413555
PVRIG	0.214589885	0.028660786	0.114558576	0.13356075	0.53384891
SLC25A27	0.100432873	0.26157085	0.241271908	2.604434621	2.402320095
SFRP2	2.525798426	7.959114643	5.999213871	3.151128198	2.375175235

C20orf26	0.080275321	0.016082448	0.048211754	0.200341125	0.600580024
PILRA	0.194207107	0.622522723	0.259193131	3.205457995	1.334622275
SBSPON	0.74021645	1.923351538	1.095681294	2.598363678	1.480217432
FOXB1	0.817471267	0.421130862	0.37406241	0.515162892	0.45758478
FOXA1	0.025204788	0.060594667	0.343116492	2.404093496	13.61314721
SAMD9L	0.011943388	0.047855034	0.038255787	4.006822493	3.20309346
RPGRIP1L	0.509885282	0.993901533	1.125590823	1.949264997	2.207537385
NPNT	0.19766168	0.532796816	0.805800336	2.695498768	4.076664404
NPR1	0.060008582	0.176325408	0.080088791	2.938336495	1.334622275
CPS1	0.044370929	0.106671862	0.118436861	2.404093496	2.66924455
CD164L2	0.055355545	0.399239719	0.177308985	7.212280488	3.20309346
LAMC3	0.097793586	0.380646069	0.223744283	3.892341851	2.2879239
ATP2A3	0.592944578	1.649101137	1.145140189	2.781206201	1.931276939
PNLIPRP3	0.036895706	0.20696836	0.206815688	5.609551491	5.605413555
C20orf112	0.204875814	0.608432522	0.772042801	2.969762554	3.768345247
ATP2C2	0.049073839	0.078652064	0.196485115	1.602728997	4.003866825
COL21A1	0.019683961	0.063096111	0.031524784	3.205457995	1.60154673
BEST3	0.120600487	0.03865798	0.057944195	0.320545799	0.480464019
FAM107B	1.282082689	1.824548307	4.248238631	1.423112817	3.313544959
BEST1	0.092025044	0.258109611	0.128959607	2.804775745	1.401353389
NUTM2A	0.203543446	0.118627267	0.07408735	0.582810544	0.363987893
NUTM2G	0.040625744	0.162780143	0.065064027	4.006822493	1.60154673
ACSS1	0.231374753	0.037083103	0.055583622	0.1602729	0.24023201
PARP10	0.605402105	1.319601892	1.105320928	2.179711436	1.825763272
ALOX12B	0.102148902	0.163717007	0.29992644	1.602728997	2.936169005
DISC1	0.011906664	0.076332626	0.066741778	6.410915989	5.605413555
PRELP	0.043913497	0.175953588	0.058607931	4.006822493	1.334622275
SULT1A1	0.422906596	0.112967444	0.3010243	0.2671215	0.711798547
WNT9A	0.206110123	0.330338671	0.49514249	1.602728997	2.402320095
PPARA	0.211941945	0.339685501	0.556673283	1.602728997	2.626536637
BOD1L1	0.301565254	0.674114499	0.660907474	2.235385181	2.191590262
NRG2	0.172759816	0.017305448	0.051878047	0.100170562	0.300290012
CCDC78	0.261593196	0.139754334	0.034912811	0.534242999	0.133462228
CCDC87	0.087676347	0.187361899	0.163820729	2.136971996	1.868471185
ZBED6CL	0.029652692	0.142575688	0.047490172	4.808186992	1.60154673
NRTN	1.080335669	1.917001586	2.471725798	1.774449961	2.2879239
COX6B2	0.256140061	0.04105231	0.041022028	0.1602729	0.160154673
RARRES1	0.609386153	1.85569363	3.96890563	3.045185095	6.512956702
ALPL	0.295350407	1.025627769	0.68324747	3.472579494	2.313345277
FAM154B	0.027209257	0.130827196	0.130730691	4.808186992	4.80464019
MYOCD	0.019791424	0.039650361	0.039621113	2.003411247	2.001933413
ACTA2	26.21670742	58.2508464	54.48257301	2.221897871	2.0781623
ACTA1	0.171183223	0.091453439	0.045692989	0.534242999	0.266924455
ASB16	0.159312172	0.351084578	0.318932361	2.203752371	2.001933413
FAM156B	0.191013866	0.393613022	0.458876449	2.060651568	2.402320095
EYA4	0.927791221	2.122569573	1.677630157	2.287766391	1.808197921
NFATC2	0.045284882	0.136086363	0.126920246	3.00511687	2.802706778
CSF1	114.7297672	239.5254442	289.4903468	2.087735817	2.523236592
GOLGA8B	0.117614612	0.397953627	0.324406902	3.383538994	2.758219368

TSSK3	0.711552032	1.814312619	2.0719706	2.549796132	2.911903146
TSSK4	0.15194801	0.446474383	0.567820956	2.938336495	3.73694237
CCDC33	0.463890817	0.721623874	1.17996801	1.555589909	2.543633042
CCDC18	0.281317064	0.763982666	0.438027357	2.715735246	1.557059321
CCDC19	0.142382481	0.380334217	0.266037562	2.671214996	1.868471185
ZGLP1	0.086052711	0.344797939	0.413452315	4.006822493	4.80464019
MAGIX	0.751619514	0.373854535	0.352824382	0.497398654	0.469418869
CCDC65	0.046937843	0.188071603	0.187932871	4.006822493	4.003866825
SNAP91	0.150916181	0.498872838	0.302124145	3.305628557	2.001933413
CLDN22	0.062918895	0.22689436	0.176343214	3.606140244	2.802706778
LMO2	0.222821755	0.059520481	0.118953151	0.2671215	0.53384891
AMPH	0.137739991	0.082784954	0.027574629	0.601023374	0.200193341
RASL12	0.090277836	0.337612112	0.433752517	3.739700994	4.80464019
CHRNA3	0.051351528	0.205756456	0.164483742	4.006822493	3.20309346
FAM163A	0.10763384	0.43126969	0.344761248	4.006822493	3.20309346
VAT1L	0.045242796	0.126895896	0.12680229	2.804775745	2.802706778
FAM167A	0.45335961	0.252734815	0.189411287	0.557470956	0.417794799
CLDN4	0.41874913	0.671141374	1.341292601	1.602728997	3.20309346
CLDN7	0.212026342	0.067964153	0.135828038	0.320545799	0.640618692
UGT8	0.040879167	0.114656896	0.114572319	2.804775745	2.802706778
COL24A1	0.01310649	0.052515378	0.125943936	4.006822493	9.60928038
HAP1	0.238208954	0.399138235	0.606936228	1.675580315	2.547915252
RBPMS	0.022289949	0.142898989	0.142793578	6.410915989	6.40618692
PTPRD	0.016853053	0.040516316	0.040486428	2.404093496	2.402320095
NTN3	0.085792733	0.240629378	0.206101607	2.804775745	2.402320095
PPP5D1	0.088788102	0.320182349	0.213297442	3.606140244	2.402320095
SPATA25	0.494154201	1.583990533	0.870552149	3.205457995	1.761701403
ANK2	0.125736485	0.293122198	0.329519221	2.331242178	2.620712831
ANK1	4.223506636	8.164181091	9.512571467	1.933033802	2.252292298
ALPK3	0.023410878	0.056281939	0.056240422	2.404093496	2.402320095
KIAA1211L	0.21298046	0.68269992	0.477537424	3.205457995	2.242165422
WNK2	0.485926293	1.458194003	0.885861726	3.000854293	1.823037235
CTSS	0.020743166	0.033245674	0.049831725	1.602728997	2.402320095
ANO4	0.100819153	0.048475734	0.032293317	0.480818699	0.320309346
ANO2	0.114505624	0.073408594	0.036677222	0.641091599	0.320309346
SCARA5	0.020523292	0.148019737	0.723118239	7.212280488	35.23402806
FAM132A	0.081679946	0.458187866	0.196221377	5.609551491	2.402320095
SBK1	0.921365501	2.187702527	2.090447366	2.374413329	2.268857868
ROPN1L	0.187098501	0.549758354	0.299646993	2.938336495	1.60154673
ANXA8	0.039132836	0.094078997	0.062673066	2.404093496	1.60154673
ANXA3	0.157958314	0.042194062	0.042162937	0.2671215	0.266924455
PRSS16	0.178412951	0.09531587	0.07143417	0.534242999	0.400386683
AOC3	0.021129014	0.067728167	0.033839103	3.205457995	1.60154673
PRSS35	0.06533143	0.445011451	0.366209681	6.811598239	5.605413555
C11orf16	0.04425568	0.106394793	0.07087754	2.404093496	1.60154673
TMCC3	0.435914297	0.79180615	1.361367379	1.816426197	3.123016124
BTN3A1	1.05702543	2.00362897	2.278871029	1.895535256	2.15592829
LRRIQ3	0.237883935	0.09531587	0.119056949	0.400682249	0.500483353
SCG2	1.75551546	1.884591536	5.596556126	1.073526027	3.187984529

USP53	0.310024628	0.71427285	0.786154971	2.303922934	2.535782323
AOX1	6.164087912	2.628961994	3.428955967	0.426496512	0.556279536
CAMK1D	0.985486748	1.545862481	2.54375443	1.56862838	2.581216272
RAPGEF3	0.05479478	0.087821183	0.219391002	1.602728997	4.003866825
SRCIN1	0.398771926	0.69722545	1.103125964	1.748431633	2.766307988
PRKAA2	0.12753321	0.248201425	0.320964907	1.946170925	2.51671629
APLN	9.697745534	4.129233386	3.491389356	0.425793126	0.360020723
APLF	0.132801534	0.195107797	0.354479771	1.469168248	2.66924455
CERKL	0.543779899	1.597808323	2.135751397	2.938336495	3.927602695
SDC2	1.760068081	4.760289254	4.169521268	2.704605183	2.368954538
SDC3	6.404801975	12.65147869	13.46808201	1.975311453	2.102810057
APOE	38.56719737	64.29631419	94.47067877	1.667124359	2.449508526
APOB	0.042231095	0.024173214	0.01449323	0.572403213	0.343188585
DYNC1I1	0.111874175	0.067238994	0.022396465	0.601023374	0.200193341
FGF14	0.030092612	0.120575754	0.048194724	4.006822493	1.60154673
CYP26B1	0.037397798	0.209784874	0.239577285	5.609551491	6.40618692
PRKCH	0.121494843	0.194723309	0.486449173	1.602728997	4.003866825
SDPR	0.026204917	0.14699783	0.188857794	5.609551491	7.206960285
PRKD1	0.254719768	0.463916771	0.723176311	1.821282952	2.839105567
FREM2	0.015812445	0.038014597	0.139284035	2.404093496	8.808507015
ASGR1	0.169931218	0.453922819	0.408229181	2.671214996	2.402320095
ASH1L	1.418181425	2.980361464	2.815928415	2.101537512	1.985591099
MAP2K6	0.128494999	0.377561544	0.342984575	2.938336495	2.66924455
AQP3	0.182131874	0.547327568	0.510462238	3.00511687	2.802706778
DYNC2H1	0.236316738	0.563143959	0.478070273	2.383004957	2.023006396
SVEP1	0.227749682	0.536470287	0.375804843	2.355525951	1.650078449
C15orf27	0.03551154	0.085373062	0.085310086	2.404093496	2.402320095
C22orf24	0.06831771	0.218989549	0.164121007	3.205457995	2.402320095
RNASE4	1.167016222	1.834441302	2.839489425	1.571907286	2.433119071
UBE2Q2L	1.881755073	1.096706698	0.684936064	0.582810544	0.363987893
GPRC5B	0.182480199	0.376028108	0.626251212	2.060651568	3.43188585
PFKFB1	0.097029823	0.038878128	0.038849449	0.400682249	0.400386683
CNTNAP3	4.486344973	11.69650537	13.79886098	2.607134636	3.075746752
TAS2R20	0.445100231	0.285350019	0.106927323	0.641091599	0.24023201
ARR3	0.063011971	0.201982225	0.100916615	3.205457995	1.60154673
EPHB3	0.040375443	0.097066339	0.096994737	2.404093496	2.402320095
EPHA5	0.060699811	0.259427591	0.105314715	4.273943993	1.735008958
TAS2R14	0.089299983	0.357809182	0.214527145	4.006822493	2.402320095
PIK3R1	2.308433378	4.580696236	5.829233072	1.98433114	2.525190082
LKAAEAR1	0.294443033	0.078652064	0.078594046	0.2671215	0.266924455
KLHL38	0.155035822	0.34166056	0.310371392	2.203752371	2.001933413
CCND2	0.052249116	0.157014701	0.073219476	3.00511687	1.401353389
C9orf163	0.09933018	0.185732586	0.212109233	1.869850497	2.13539564
C9orf169	0.32842014	0.684279025	0.631176241	2.083547697	1.921856076
MAML2	0.802682652	1.516897586	1.909113595	1.889784937	2.378416413
MAML3	0.011997209	0.048070689	0.038428183	4.006822493	3.20309346
SLC2A12	0.060862428	0.451150152	0.450817357	7.412621613	7.407153627
ANKRD65	0.038340317	0.092173707	0.061403809	2.404093496	1.60154673
CD177	0.180186515	0.548701289	0.259719411	3.045185095	1.441392057

CD180	0.048349707	0.096864347	0.212944368	2.003411247	4.404253508
ANKRD12	0.097654386	0.210691272	0.216551164	2.157519804	2.217526242
EIF4E3	0.013720758	0.032985984	0.12085939	2.404093496	8.808507015
GRASP	0.027375381	0.153563607	0.087685902	5.609551491	3.20309346
SGCG	0.048764845	0.117235247	0.078099178	2.404093496	1.60154673
NLGN3	0.171025715	0.479688777	0.855955232	2.804775745	5.004833531
NLGN1	0.068758825	0.22827508	0.220240943	3.319938637	3.20309346
FAAH	0.569303379	0.228109758	0.293067632	0.400682249	0.514782878
STAG3	0.01945027	0.062347025	0.093451551	3.205457995	4.80464019
MAP4K1	0.03029594	0.097112364	0.097040728	3.205457995	3.20309346
STAC3	0.154333667	0.371032565	0.37075887	2.404093496	2.402320095
STAC2	0.025997005	0.041666153	0.145723962	1.602728997	5.605413555
SOHLH2	0.155389301	0.062261735	0.031107903	0.400682249	0.200193341
LATS1	0.679996149	1.61661016	1.343156251	2.377381346	1.975240967
ARID3C	0.068758825	0.330605288	0.440481885	4.808186992	6.40618692
FAT2	0.017369618	0.060317378	0.03245463	3.472579494	1.868471185
MPV17L	0.294910201	0.679450375	0.531351526	2.303922934	1.801740071
MFSD2B	0.045146891	0.144716464	0.108457285	3.205457995	2.402320095
PCDHB14	0.086256008	0.32257168	0.207214542	3.739700994	2.402320095
ZFAND4	0.28984517	0.125069339	0.160684818	0.431503961	0.55438156
MAN1C1	0.091782142	0.279493611	0.27928744	3.045185095	3.042938787
SMOC1	0.728917084	2.007940941	2.809043671	2.754690464	3.853721819
PPIL6	0.020054657	0.112497633	0.192710825	5.609551491	9.60928038
FCN2	0.078445842	0.565773414	0.125634681	7.212280488	1.60154673
PCDHA11	0.012440447	0.029907999	0.039847916	2.404093496	3.20309346
PRRT4	0.022657496	0.145255302	0.054430557	6.410915989	2.402320095
JPH3	0.021282084	0.068218828	0.136337011	3.205457995	6.40618692
STON1-GTF2A1L	0.06673017	0.124775442	0.142495315	1.869850497	2.13539564
FRMD5	0.536792858	0.784421702	1.403331941	1.461311733	2.614289515
S100A8	0.162891366	1.04428286	0.652195335	6.410915989	4.003866825
PPM1N	0.521584801	0.905622343	1.287819584	1.736289747	2.469051209
PPM1J	0.894667045	1.553401218	2.427886554	1.736289747	2.713731959
PPM1K	0.051709975	0.13467525	0.207039854	2.604434621	4.003866825
PPM1E	0.104131012	0.312925861	0.354387699	3.00511687	3.403286801
TPD52L1	1.837358394	4.417181365	5.836187067	2.404093496	3.176401015
MCOLN2	0.671686078	0.336415861	0.20170062	0.500852812	0.300290012
RASSF5	2.847584057	0.980673918	1.413390166	0.344388049	0.496347127
DCLK1	0.100664285	0.475945826	0.773849069	4.728050542	7.687424304
C5orf42	0.223987338	0.38142794	0.515668897	1.70289956	2.302223424
C5orf54	0.667697917	0.267534703	0.315944145	0.400682249	0.473184261
CRYBB3	0.78973056	0.316431017	0.1580988	0.400682249	0.200193341
CPXM1	0.638941381	1.877424779	2.47296166	2.938336495	3.870404598
CNTFR	0.581233226	1.530418924	1.163590215	2.633054781	2.001933413
SOX15	20.47301414	7.982789593	7.927962974	0.389917652	0.387239657
HOXA7	0.042216147	0.067661043	0.270444527	1.602728997	6.40618692
HOXA4	0.246505163	0.118524292	0.078957907	0.480818699	0.320309346
HOXB9	1.577048948	2.729788646	7.627667111	1.730947317	4.836671125
ELANE	0.066608432	0.320265795	0.106676516	4.808186992	1.60154673
CNTD1	0.103988019	0.06249923	0.020817709	0.601023374	0.200193341

CLSTN3	3.305239259	5.733119918	7.699629277	1.734555192	2.329522516
SERINC2	0.060635007	0.145772225	0.169942144	2.404093496	2.802706778
CCT6B	0.179540957	0.647449871	0.467257754	3.606140244	2.602513436
LOC101929097	0.194354793	0.726828812	1.141317037	3.739700994	5.87233801
STEAP1	0.908370585	0.155986273	0.623484832	0.171720964	0.68637717
DPYSL3	0.031092038	0.087206194	0.074693028	2.804775745	2.402320095
CACNA2D3	0.068262968	0.237048583	0.127547389	3.472579494	1.868471185
FEZ1	0.045900961	0.073566802	0.147025069	1.602728997	3.20309346
TXNIP	6.453516828	2.239495848	2.606972749	0.347019448	0.403961564
BTBD19	0.242614031	0.583266815	0.444065953	2.404093496	1.83033912
BTBD11	0.443709293	0.99560405	2.510861456	2.243820596	5.658798446
LOC101929668	0.162332668	0.182122692	0.493968372	1.121910298	3.042938787
PLSCR4	0.744470005	1.511365974	1.748711802	2.030123397	2.348935204
CASP8AP2	0.352710507	0.686434933	0.756538873	1.946170925	2.144928656
ALDH1L1	0.284923693	0.020757067	0.04148351	0.072851318	0.145595157
TNFRSF14	0.084712149	0.362054979	0.520070115	4.273943993	6.139262465
TNFRSF1B	0.20863392	0.297229897	0.705400277	1.424647998	3.381043097
NBEAL1	0.092691793	0.303872368	0.249666309	3.278309313	2.69351041
GRIK4	0.014800588	0.047442663	0.047407666	3.205457995	3.20309346
GRIP2	0.074758091	0.171167085	0.136832658	2.289612853	1.83033912
CNTN3	0.253246683	0.446474383	0.635418689	1.763001897	2.509089877
CNTN4	0.049092692	0.07868228	0.11793636	1.602728997	2.402320095
TTC36	0.752802806	0.402179629	0.200941479	0.534242999	0.266924455
LOC100507462	0.062826095	0.125866504	0.251547316	2.003411247	4.003866825
TTC16	0.059079185	0.236719806	0.236545187	4.006822493	4.003866825
OPN1SW	0.775885102	1.865300327	1.55327031	2.404093496	2.001933413
LOC101929747	0.12727916	0.509985	0.305765283	4.006822493	2.402320095
SLC4A4	0.594594539	0.114709822	0.246885058	0.192921083	0.415215819
AC022007.5	0.718542408	1.115640354	1.87001627	1.552643716	2.602513436
PLAC9	31.26667087	15.01331176	12.16397599	0.480169821	0.389039692
LOC101929792	0.147774821	0.059210748	0.05916707	0.400682249	0.400386683
MYZAP	0.319737138	0.085408664	0.199139877	0.2671215	0.622823728
ADCY1	0.06134328	0.016386109	0.010916014	0.2671215	0.177949637
ADCY7	0.080827499	0.226703009	0.183386108	2.804775745	2.268857868
FGD6	0.082550566	0.183758592	0.242382413	2.226012496	2.936169005
RSPH4A	0.029704388	0.166628293	0.09514593	5.609551491	3.20309346
NOXRED1	0.096226112	0.051408126	0.025685102	0.534242999	0.266924455
KIAA1407	0.1902083	0.406469811	0.558483715	2.136971996	2.936169005
SLC51A	0.058551329	0.093841913	0.37509076	1.602728997	6.40618692
TIAM2	0.014400302	0.115398905	0.126845158	8.013644987	8.808507015
HMX2	0.156988054	0.167739538	0.50284741	1.068485998	3.20309346
GRHL3	0.089082103	0.166570214	0.190225534	1.869850497	2.13539564
GRHL1	0.115876203	0.130002705	0.371162307	1.121910298	3.20309346
MAGED4B	0.223266675	0.281156837	0.612980595	1.259287069	2.74550868
OAS1	0.804690036	0.252332619	0.308179036	0.313577413	0.382978566
SNCB	0.05875323	0.282496519	0.188192088	4.808186992	3.20309346
SNCA	0.370976852	0.976802374	1.124616018	2.633054781	3.031499168
FGFBP1	0.062687406	0.200941845	0.250992024	3.205457995	4.003866825
RIPK4	3.080295916	6.453206887	11.76929602	2.094995761	3.820832913

FNDC1	1.66457023	3.772513677	3.717662743	2.266358973	2.233406963
CHRDL2	0.341965616	0.71935265	1.06111821	2.103581809	3.10299679
CCDC116	0.186334611	0.089593165	0.059684717	0.480818699	0.320309346
CAPS2	0.036037303	0.043318523	0.115430849	1.202046748	3.20309346
CCDC169	0.852632369	0.170817328	0.398279753	0.200341125	0.467117796
CCDC153	0.219379015	0.937613624	1.229710105	4.273943993	5.605413555
CCDC150	0.092474555	0.277896847	0.203640693	3.00511687	2.202126754
CCDC147	0.231430624	0.061820095	0.144140484	0.2671215	0.622823728
CCDC175	0.065281367	0.026157085	0.02613779	0.400682249	0.400386683
PLCE1	1.339009462	2.278361235	3.47377392	1.70152736	2.594286313
NHLRC4	0.041679151	0.133600767	0.133502215	3.205457995	3.20309346
RIPPLY2	0.384904447	1.439427542	1.130144506	3.739700994	2.936169005
LOC101929571	0.87927713	1.80069933	1.721137505	2.047931497	1.957446003
ENKUR	0.025189883	0.040372556	0.060514162	1.602728997	2.402320095
CSF1R	0.042713554	0.102687378	0.171019383	2.404093496	4.003866825
KIAA1644	0.416618565	1.51756058	1.13227605	3.642565903	2.717776269
1-Mar	0.015483858	0.037224641	0.061995304	2.404093496	4.003866825
DDX60L	0.172553614	0.483974191	0.473747445	2.804775745	2.74550868
SOX7	2.754841832	0.999682616	1.602474578	0.362882037	0.58169386
SOX4	2.147702123	4.78547597	4.642122941	2.228184216	2.16143705
CREBRF	0.085394997	0.205297558	0.264980403	2.404093496	3.10299679
ERO1LB	0.084281939	0.202621662	0.269962928	2.404093496	3.20309346
MAGEB17	0.875048114	2.253961584	2.102145667	2.57581446	2.402320095
KIAA1109	0.27141438	0.712211935	0.677593796	2.624075907	2.496528726
SPATA1	0.199140216	0.095750339	0.031893236	0.480818699	0.160154673
ITGAM	0.017954096	0.02877555	0.115017293	1.602728997	6.40618692
ITGB4	14.64747301	28.79698983	34.77022449	1.966003953	2.373803623
ITGB8	0.165007079	0.707823775	0.792799643	4.289657022	4.80464019
ITGA4	1.876973476	4.063422739	5.462505885	2.164880213	2.910273349
ITGA2	2.55253447	3.883880707	7.494672594	1.521578162	2.936169005
ITGA7	0.832620192	1.698409396	2.727573026	2.039836906	3.275891039
LRRC16B	0.03704008	0.05936521	0.133473193	1.602728997	3.603480143
DDR1	6.626058771	12.38973929	14.23347878	1.869850497	2.148106328
RP11-35G9.3	0.128948816	0.34445001	0.58513307	2.671214996	4.537715735
MAGEA12	0.457530527	1.026616421	2.01508043	2.243820596	4.404253508
LOC101927402	0.070406764	0.02821074	0.028189931	0.400682249	0.400386683
LOC101927562	0.321884827	0.472902967	1.28878398	1.469168248	4.003866825
C1QTNF6	0.124732334	0.359841832	0.199764662	2.884912195	1.60154673
KIAA1024	0.037908121	0.091134667	0.091067441	2.404093496	2.402320095
GRAMD1C	0.1585603	0.290433361	0.435328681	1.831690283	2.74550868
CXorf22	0.188634784	0.037791305	0.094408569	0.200341125	0.500483353
NLRC4	0.02524961	0.101170705	0.060657646	4.006822493	2.402320095
SLC22A31	0.120271319	0.032127055	0.032103356	0.2671215	0.266924455
LRRC37A	0.013954494	0.055913179	0.044697548	4.006822493	3.20309346
IQGAP2	0.043778101	0.175411079	0.233708914	4.006822493	5.3384891
ABCA1	0.67323158	1.209008713	2.104461656	1.795828877	3.125910485
ZBTB8B	0.211973586	0.492617509	0.458305565	2.323957046	2.162088086
SRPX	3.044176839	5.488864305	7.958359594	1.803070122	2.614289515
FABP3	0.232977714	1.369133841	0.559687045	5.87667299	2.402320095

ZBTB20	0.009223926	0.014783454	0.036931373	1.602728997	4.003866825
ITIH4	0.051898985	0.124770013	0.24935595	2.404093496	4.80464019
ZBTB41	0.100533614	0.265861427	0.193211136	2.644502846	1.921856076
IL12A	0.236152971	0.283866911	0.803696288	1.202046748	3.403286801
ST8SIA4	0.282985743	0.129585559	0.151071631	0.457922571	0.53384891
ST8SIA6	0.060142735	0.096392505	0.144482101	1.602728997	2.402320095
ST8SIA1	0.008770042	0.091364	0.105342236	10.41773848	12.01160048
DAPK2	0.030777523	0.086324051	0.110906194	2.804775745	3.603480143
ACE	0.154302608	0.384697077	0.906117068	2.493133996	5.87233801
SSPN	0.018793775	0.090363987	0.150495548	4.808186992	8.00773365
GARNL3	0.227908465	0.566177034	0.693511508	2.484229946	3.042938787
LOC101927371	0.05457539	0.437347803	0.655537784	8.013644987	12.01160048
MOB3B	0.026112547	0.125554008	0.20910232	4.808186992	8.00773365
NOTCH4	0.02523839	0.050562874	0.080840921	2.003411247	3.20309346
AK7	0.051631627	0.206878764	0.124035695	4.006822493	2.402320095
UGT3A2	0.035719993	0.114498938	0.171621716	3.205457995	4.80464019
RCAN2	0.124622856	0.33955233	0.518932251	2.724639295	4.164021498
C16orf45	9.694809492	16.57810737	26.43816384	1.709998261	2.727043153
BAI2	4.200611852	10.26256204	11.98095476	2.443111243	2.852192772
AMN	0.16414679	0.043847137	0.043814793	0.2671215	0.266924455
TRAF5	0.252969026	0.483410176	0.5921302	1.910946112	2.340722144
NFAT5	0.700884409	1.857032838	1.658467188	2.649556496	2.366249223
LOC102723974	0.12205184	0.489040057	0.586415175	4.006822493	4.80464019
MAP7	0.055463662	0.118524292	0.222069115	2.136971996	4.003866825
SLC2A1	63.51112152	32.36778911	24.48099601	0.509639703	0.385459986
SLC2A4	0.081161179	0.151759271	0.368286359	1.869850497	4.537715735
SLC2A5	0.069886944	0.476041782	0.251836213	6.811598239	3.603480143
ABCC6	0.066595415	0.200127004	0.479950509	3.00511687	7.206960285

Table S5: Correlation analysis of the gene expression between c-Myc targets and LacRNA, TCGA database, n=1094

Gene Name	Pearson correlation coefficient	-LOG10(FDR)	*P* value (two-tailed)
CDC20	-0.2353	12.99999218	1.00002E-13
CDC45	-0.2837	13	1E-13
VBP1	-0.2538	12.99999409	1.00001E-13
IFRD1	0.07467	1.869666232	0.0135
CCT2	-0.2463	12.99999661	1.00001E-13
CCT5	-0.223	11.99995093	1.00011E-12
NPM1	-0.1433	4.217443535	6.06117E-05
DUT	-0.2134	11.14532967	7.156E-12
NOP16	-0.1696	8.097040321	7.9976E-09
RAN	-0.2833	13	1E-13
MRPL9	-0.1346	6.807014621	1.5595E-07
CCNA2	-0.243	13	1E-13
PSMA4	-0.1686	8.847011843	1.42229E-09
EEF1B2	0.096415	2.804931004	0.001567
AP3S1	0.086	2.332080315	0.004655
SNRPB2	-0.138956	5.301029996	0.000005
LSM7	-0.106716	3.335358024	0.000462
PCNA	-0.343797	13	1E-13
CCT4	-0.164508	6.224025669	0.000000597
PPIA	-0.179203	7.468859709	3.39735E-08
PSMA7	-0.253531	12.9998541	1.00034E-13
AIMP2	-0.242792	12.99682457	1.00734E-13
EIF4A1	-0.171278	7.783886903	1.6448E-08
SRM	-0.061536	1.357782871	0.043875
TRA2B	-0.118951	4.026872146	0.000094
NME1	-0.21612	12.0308055	9.31525E-13
ODC1	-0.010256	0.132416441	0.737197
RFC4	-0.25069	12.99966546	1.00077E-13
PWP1	-0.099451	2.956244873	0.001106
RPS5	0.012517	0.166133487	0.682129
PSMA2	-0.179313	8.477881936	3.3275E-09
RRP9	-0.182122	7.729093015	1.86598E-08
POLD2	-0.282565	15	1E-13
MAD2L1	-0.329241	15	1E-13
CDKN1A	0.081253	2.110810388	0.007748

Table S6: Univariate and multivariate Cox regression analysis of OS and DFS in patients with breast cancer

Covariates	Univariate analysis		Multivariate analysis	
	HR (95% CI)	*P*-value	HR (95% CI)	*P*-value
DFS				
**Age**	1.213	0.335	——	
≤50 versus ＞50 years	（0.819-1.798）			
**T stage**	1.952	**0.006**	1,620	0.094
T2/3 versus T1	（1.211-3.144）		(0,921-2.847)	
**pN stage**		**0.000**		**0.000**
N1 versus N0	2.307	**0.002**	1.652	0.103
	(1.353-3.935)		(0.903-3.023)	
N2 versus N0	3.535	**0.000**	3.991	**0.000**
	(1.846-6.772)		(2.025-7.869)	
N3 versus N0	5.934	**0.000**	4.16	**0.000**
	(3.491-10.086)		(2.246-7.707)	
**ER**	0.752	0.197	——	
positive versus negative	(0.488-1.159)			
**HER2**	1.181	0.456	——	
positive versus negative	(0.763-1.828)			
**Ki-67**	1.508	0.189	——	
positive versus negative	(0.817-2.784)			
**Subtype**		0.179		0.634
ER^+/HER^+ versus $ER^+/HER2^-$	1.745	**0.047**	1.48	0.202
	(1.008-3.020)		(0.811-2.703)	
ER^-/HER^+ versus $ER^+/HER2^-$	1.451	0.253	1.229	0.549
	(0.766-2.749)		(0.626-2.414)	
ER^-/HER^- versus $ER^+/HER2^-$	1.51	0.192	1.264	0.524
	(0.813-2.805)		(0.615-2.597)	
**Grade**	1.65	**0.016**	1.472	0.119
G3 versus G1+G2	(1.104-2.610)		(0.905-2.393)	
**LacRNA expression level**	0.483	**0.001**	0.483	**0.006**
high versus low	(0.312-0.749)		(0.287-0.811)	
OS				
**Age**	1.413	0.261	——	
≤50 versus ＞50 years	(0.773-2.584）			
**T stage**	2.562	**0.013**	1.787	0.211
T2/3 versus T1	(1.191-5.512）		(0.720-4.435)	
**pN stage**		**0.000**		**0.000**
N1 versus N0	3.972	**0.003**	3.847	**0.008**
	(1.584-9.958)		(1.422-10.411)	

N2 versus N0	6.424 (2.252-18.324)	**0.001**	8.983 (2.916-27.675)	**0.000**
N3 versus N0	12.173 (5.081-29.164)	**0.000**	8.447 (3.095-23.051)	**0.000**
**ER** positive versus negative	0.561 (0.306-1.026)	0.061	——	
**HER2** positive versus negative	1.606 (0.878-2.937)	0.124	——	
**Ki-67** positive versus negative	1.586 (0.657-3.832)	0.305	——	
**Subtype**		0.066		0.154
ER^+/HER^+ versus $ER^+/HER2^-$	2.355 (1.087-5.104)	**0.030**	2.369 (.011-5.547)	**0.047**
ER^-/HER^+ versus $ER^+/HER2^-$	1.904 (0.755-4.802)	0.172	1.737 (0.645-4.674)	0.274
ER^-/HER^- versus $ER^+/HER2^-$	2.488 (1.080-5.733)	**0.032**	2.569 (0.953-6.930)	0.062
**Grade** G3 versus G1+G2	3.124 (1.817-6.502)	**0.000**	1.878 (0.908-3.881)	0.089
**LacRNA expression level** high versus low	0.338 (0.171-0.667）	**0.002**	0.318 (0.135-0.750)	**0.009**

Abbreviations: CI, confidence interval; HR, hazard ratio; DFS, disease-free survival; OS, overall survival; BC, breasr cancer.
